# Construction of a Calibration Curve for Lycopene on a Liquid-Handling Platform—Wider Lessons for the Development of Automated Dilution Protocols

**DOI:** 10.1021/acssynbio.4c00031

**Published:** 2024-08-03

**Authors:** Matthieu Bultelle, Alexis Casas, Richard Kitney

**Affiliations:** Department of Bioengineering, Imperial College London, London SW7 2BX, United Kingdom

**Keywords:** calibration curve, metrology, dilution, liquid handling-automation, lycopene

## Abstract

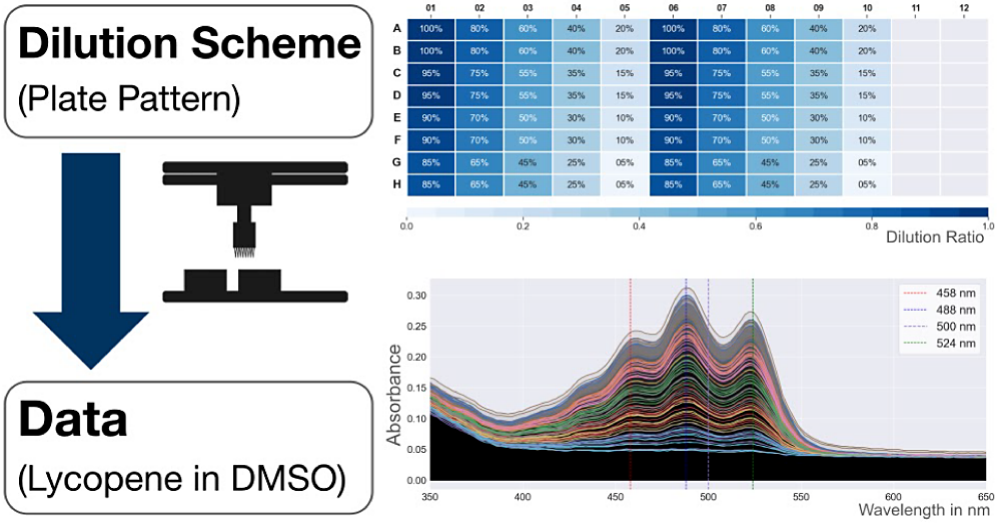

Liquid-handling is a fundamental operation in synthetic biology—all protocols involve one or more liquid-handling operations. It is, therefore, crucial that this step be carefully automated in order to unlock the benefits of automation (e.g., higher throughput, higher replicability). In the paper, we present a study, conducted at the London Biofoundry at SynbiCITE, that approaches liquid-handling and its reliable automation from the standpoint of the construction of the calibration curve for lycopene in dimethyl sulfoxide (DMSO). The study has important practical industrial applications (e.g., lycopene is a carotenoid of industrial interest, DMSO is a popular extractant). The study was also an effective testbed for the automation of liquid-handling. It necessitated the development of flexible liquid-handling methods, which can be generalizable to other automated applications. In addition, because lycopene/DMSO is a difficult mix, it was capable of revealing issues with automated liquid-handling protocols and stress-testing them. An important component of the study is the constraint that, due to the omnipresence of liquid-handling steps, errors should be controlled to a high standard. It is important to avoid such errors propagating to other parts of the protocol. To achieve this, a practical framework based on regression was developed and utilized throughout the study to identify, assess, and monitor transfer errors. The paper concludes with recommendations regarding automation of liquid-handling, which are applicable to a large set of applications (not just to complex liquids such as lycopene in DMSO or calibration curves).

## Introduction

Liquid-handling is a basic and common operation in wet-lab biology. Consequently, almost all protocols in synthetic biology involve one or more liquid handling steps. It is therefore of the utmost importance that such fundamental steps should be reliably automated to achieve the higher throughput and higher replicability promised by automation. This is necessary for the discipline to become a fully fledged form of engineering and for the accurate industrial translation of results. In particular, liquid-handling errors (in automated setups or otherwise) should be controlled in magnitude and prevented from propagating to other parts of the protocols. Errors should be well-understood and clearly communicated to all practitioners.

In the wider context of improving replicability in synthetic biology through automation, we have conducted a study that approached liquid-handling, its automation, and the monitoring of such operations from the standpoint of vulnerability analysis. This approach will be familiar to anyone who has ever reverse engineered the inner workings of a piece of software or hardware: (a) apply stress on the system to generate errors/failures; (b) identify the modes of failure (types and their magnitudes); and (c) reverse engineer the system. To the best of the authors’ knowledge, this is the first time the problem has been approached in this manner.

Depending on the implementation (platform, labware, consumables, and of course protocol, down to the number and types of liquid transfers), some errors are more or less liable to occur. In our study, stress is applied to the system by varying the concentration of a mix that is particularly difficult to pipet, which in turn induces pipetting errors. Several lists of potential vulnerabilities exist already (see section “Automated Liquid-Handling“) and have been used as the basis for the analysis.

To make the exercise less abstract, we have organized the study around an exemplar application: the construction of a calibration curve (see “Testing Liquid-Handling Methods by Building Calibration Curves”) and more specifically, the calibration curve of lycopene in a common solvent, dimethyl sulfoxide (DMSO). Lycopene and DMSO are known to be challenging to pipet and are therefore a good choice to identify potential issues with automated liquid-handling protocols. Also, lycopene is a carotenoid with notable industrial interest, and DMSO is used as a possible extractant for lycopene. Their combination is therefore of practical use in assays for lycopene and carotenoids in general.

### Replicability and Automation

Reproducibility, replicability, and repeatability (the terms are often used interchangeably) are key principles underpinning the scientific method. For the findings of a study to be generally recognized, when the study is replicated by other groups, they should be achieved again with a high degree of reliability using both identical and different methodologies. Concerns about lack of reproducibility and replicability have recently been expressed in both the scientific^1,2^ and popular media.^3^ In 2014, a landmark editorial in the journal Science^4^ bluntly stated “*Recently, the scientific community was shaken by reports that a troubling proportion of peer-reviewed preclinical studies are not reproducible*”. This state of concern is now called the “replication crisis”. In this paper, we adopt the definitions from the committee on the “Reproducibility and Replicability in Science” of the National Academy of Sciences.^5^ “*Replicability is obtaining consistent results across studies aimed at answering the* same *scientific question, each of which has obtained its own data*”. Reproducibility is presented as a purely computational concept—of no relevance to our study. Replicability studies are rare in Synthetic Biology. The most prominent have been conducted in the context of iGEM interlab studies,^6−8^ and DARPA^9^ where identical methodologies were used by participants with markedly different outcomes.

Automation^10^ is an essential component to tackle the replication crisis, as it has been associated with an increase in the rate of data generation and a reduction in human-induced variability.^11^ Automation is a dual concept. Frohm et al.^12^ make the distinction between mechanization (the replacement of human power) and computerization (the replacement of cognitive tasks—mental activity and sensory processes).

High throughput is the feature most associated with (mechanized) automation. Bogue^13^ argues that the history of lab automation in biotechnology parallels the development of modern drug discovery and high throughput screening. The development of drugs routinely involves testing an ever-larger number of compounds (hundreds of thousands being common) against target diseases indeed. This can only be achieved within a reasonable time frame by first miniaturizing the assays and then automating them. High throughput is highly desirable for replicability, as it enables more thorough statistical analyses and helps identify discrepancies between studies. It is also crucial to tackle the complexity and intrinsic stochasticity^14^ of biology, since the behavior of a system can only be captured with a large number of repeats (technical and biological).

The reduction in human-induced variability is highly beneficial to replicability. Human error is significantly affected by stress, tediousness, repetition, and fatigue in the manufacturing environment.^15,16^ Humans also vary considerably in their ability to perform and learn new motor skills,^17^ some of which, like micropipetting, have a major influence on the output of experiments.^18^ All these confounding variables then need to be controlled through a large number of repeats with different experimentalists, by using blocking strategies, or similar design strategies, leading to ever more complex studies. It is important to note that automation does not necessarily lead to more accurate data. On occasions, a highly skilled experimentalist will collect better quality data, but automation can be expected to generate more homogeneous data.

Another, more subtle, benefit of automation is to force protocols to be precisely written before they are implemented (and shared), thus cutting out some of the communication errors between operators. It is worth noting that, even after setting protocol parameters that are consistent with shared/published research, or importing them, a host of potential practical issues^9,19^ may appear. For example, protocol parameters may be wrongly imported or may be suited to a different platform and labware. Practical considerations may also necessitate some parameters to vary throughout the assays. Recent script-sharing initiatives (on platforms such as Opentrons,^20−22^ or with agnostic languages such as LabOP^23^ and PyLabRobot^24,25^) promise to limit errors further.

Despite all the (theoretical) benefits of automation and its proven successes in other fields and industries, the degree of automation in biotechnology remains comparatively low.^26^ Claims were made as early as 2010 that automation was revolutionizing biology,^27^ but the promise remains largely unfulfilled and academic laboratories have only minimally embraced conventional lab automation tools.^16^ Several reasons for the slow adoption of automation have been put forward, ranging from the high costs to a lack of flexibility.^16^ Despite, or maybe because of, the low level of automation in synthetic biology, efforts proceed at a fast pace to unlock the benefits of automation for the domain. High-throughput platforms are in greater use,^28^ and advanced workflows such as the design–build–test–learn workflows have been successfully automated.^29,30^ Many initiatives to spread the benefits of automation have been launched. The most comprehensive is the establishment of a network of biofoundries.^31^ Biofoundries are physical locations where all the mechanized and computerized laboratory processes can be physically integrated to enable the rapid design, construction, and testing of genetically modified organisms for biotechnology applications and research.^31,32^

### Automated Liquid-Handling

Because laboratory experiments combine multistep protocols with many liquid-handling steps, robotic liquid-handling platforms are central to the transformation of research laboratories and their adoption of automation.^33^ It is therefore essential that this fundamental step of experimental workflows should be reliably automated. From a replicability viewpoint, consistency of outputs is not enough. Liquid-handling errors should be well-understood (ideally predictable), controlled in magnitude (and unbiased), and should not propagate to later parts of the experimental pipelines. Furthermore, there should be simple, practical and reliable methodologies for monitoring the quality of the liquid-handling operations.

Achieving robust, repetitive, and high-speed automated liquid-handling operations is not without its challenges.^33^ A wide range of physical factors have been identified that play a role in the dispensation of liquids, including density and viscosity of the pipetted liquid, prewetting, and environmental factors such relative humidity, vapor pressure, temperature, and air pressure.^34^ For instance, combinations of viscous material, dust, particles, or liquid debris can result in clogging of tubes, valves, and pipetting heads. Additionally, air bubbles may form during aspiration or dispensing and cause inaccuracies in volume handling. These factors are, however, very low level, and it is not immediately clear how they relate to protocol actions and parameters.

A wider, more practical view of liquid handling errors was presented by Albert,^35^ who proposed a classification tracing them back to ill-adapted equipment, ill-adapted pipetting-methods, and ill-adapted dispensing-schemes. Some of these errors affect wells independently, while some introduce correlation within and across plates, e.g., errors linked to the evolution of common resources (e.g., the volume of reagent in a reservoir).

Since Albert’s classification introduces classes of errors that are directly related to decisions made by the protocol designers, it was used as the basis for the reverse analysis.

### Testing Liquid-Handling Methods by Building Calibration Curves

The study presented in this paper was designed to better understand liquid-handling errors and help develop methodologies for monitoring liquid-handling operations. From the beginning, we aimed for the study to be conducted in a biofoundry-type location (it was run at the London Biofoundry, SynbiCITE) on industrial automated platforms. This way, our findings could be transported to similarly equipped laboratories. Also, we only considered liquid-handling schemes capable of transferring variable volumes of these solutes and solvents into the wells of the microplates and mixing them in situ. These schemes manage resources more efficiently, are less labor intensive, and can be used for any liquid handling steps in biological protocols.

As part of the study design, we placed some realistic constraints regarding data acquisition and monitoring of the liquid-handling operations. Again, these constraints meant that the methodologies we developed could be more easily adapted to other cases. They were:

- Study size: we expected dozens of plates, given the typical throughput of the equipment we would use. This was substantially more than would be collected in a manual study but would still not be enough for machine learning. Analysis would therefore have to rely on common statistical methods.
- Monitoring: analyses would be run plate by plate, which in the context of the study was the most practical scale—and usually with laboratory workflows.
- Prior knowledge: we decided not to rely on any prior knowledge on the properties of the liquids to estimate the volumes of liquid that were dispensed (which is what hardware manufacturers typically do when they list the errors for their automated platforms). This was, of course, so that our methodologies were more generalizable, and also because the liquids used for such tasks are well-behaved and reveal little on the types and magnitude of transfer errors. On the downside, we would have to rely on our measurements alone to estimate the properties of the content of the wells.

Finally, and as previously mentioned, we organized the study around an exemplar application: the construction of a calibration curve. A calibration curve (also known as a standard curve) is *″a regression of the assay response on the known concentrations in “standard” samples. It is a model that fits data from standards and is used for calculating (calibrating) values of unknown test samples.″*(36) Since it necessitates the generation of measurement microplates with possibly a large number of different concentrations and arranged in possibly complex patterns, and for the process to be repeated over a series of assays, the construction of a calibration curve was deemed a good testbed for the automation of liquid handling. In addition, the link between calibration curve and regression could be exploited to develop metrics, quantifying the reliability of the liquid-handling process (consistency within plates and between plates), which we planned to use for monitoring.

### Dilution Schemes and Plate Patterns

**Figure 1:**
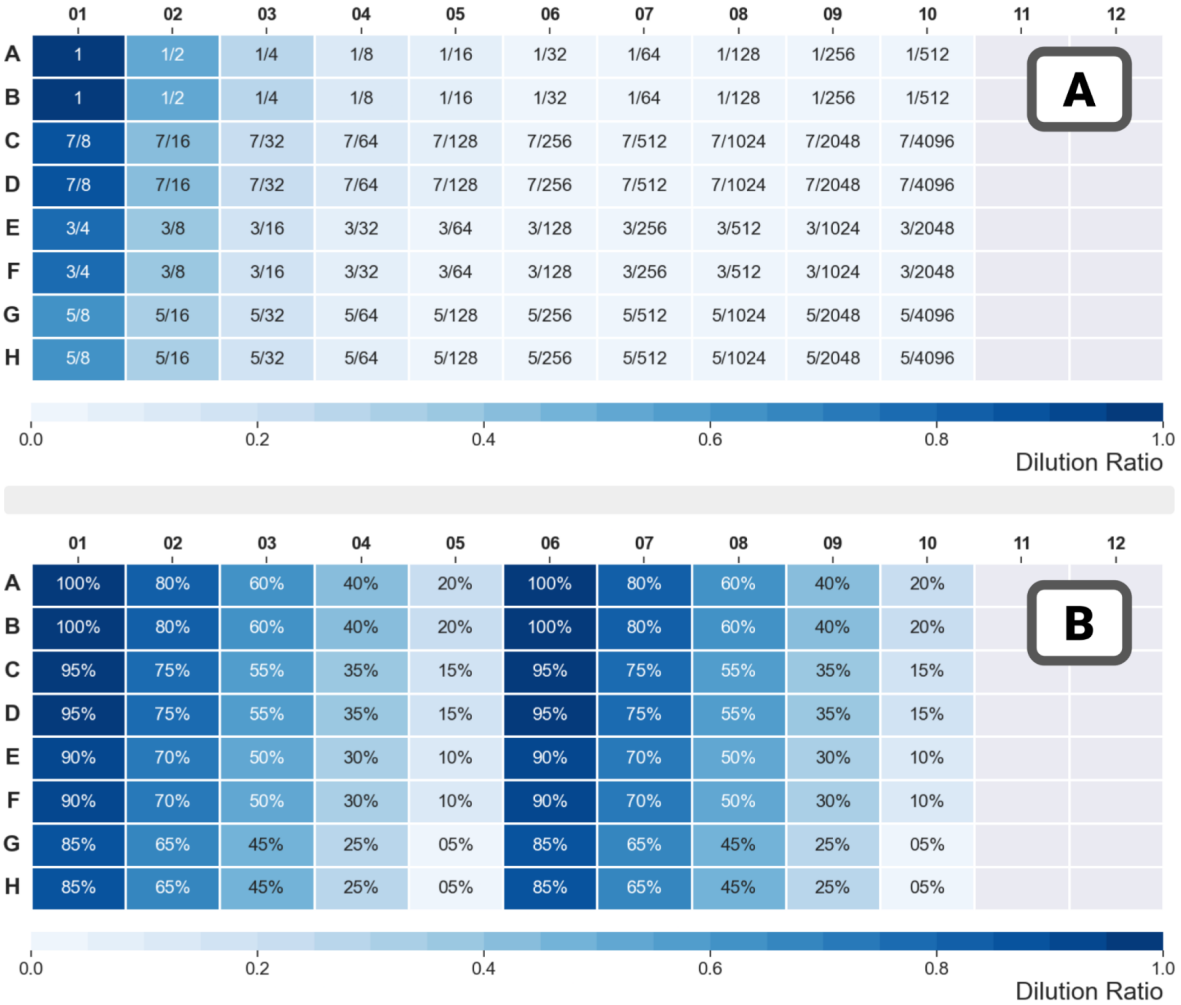
The geometric and linear dilution schemes. (A) Geometric dilution scheme plate map of the 96-well measurement plate. Initial concentration of the standard is in well A1, and fractions of the initial concentration in C01, E01, and G01 are, respectively, 7/8, 3/4, and 5/8 of the initial concentration (with duplicated technical repeats every other rows), the subsequent columns contain a dilution scheme of 1:2. Blanks (containing only the diluent) are located in columns 11 and 12. All wells contain an overall volume of 200 μL. (B) Linear dilution scheme plate map of the 96-well measurement plate. Initial concentration of the standard is in well A1 and each successive dilution has a step of 5% going from top to bottom from rows A to H (with technical repeats every other row). The pattern contains two identical blocks (columns 01–05 and 06–10). The same blanks were used as for the first scheme. Likewise, all wells contained an overall volume of 200 μL.

Two different dilution schemes (Figure 1) were chosen to be stress-tested. These dilution schemes were chosen because they are representative of two common approaches to dilution and were also capable of generating plate patterns suitable for the construction of a calibration curve.

The first dilution scheme is a direct adaptation of a well-known manual protocol (the serial dilution protocol^37,38^) and is based on an iterative transfer workflow (transferring liquid from column to column). In this paper, we henceforth refer to it as the “geometric dilution scheme”. In our implementation (Figure 1A), the first column is created by direct dilution so concentrations vary linearly, before iterative dilution is performed to generate concentrations of lycopene that halve with every column. The second dilution scheme (the “linear dilution scheme”) is based on a direct transfer workflow (that only transfers liquid from reservoirs to wells). Our implementation (Figure 1B) generates a linear range of dilutions between 0 and 1, with an increment of 5%—repeated over two adjacent blocks. Readers interested in a more thorough comparison between the two schemes are referred to Section 1 of the Supporting Information. For both dilution schemes, dilutions were performed on a CyBio Felix,^39^ liquid-handling robotic platform with an 8-channel SELECT Head,^40^ see Methods.

The schemes vary in terms of complexity for their implementation (the cognitive load they exert), which, in turn, determines how amenable they are to manual implementation at even modest scales. As previously stated, the first scheme is a direct adaptation of a well-known manual protocol and is very simple. Conversely, even with the predictable spatial pattern, the large number of different dilution ratios makes the second scheme cumbersome to implement manually at even the most modest scale. The schemes also conspicuously vary in terms of flexibility and what plate pattern they can generate—iterative transfers being much more limited.

Finally, and of most relevance for this study, the schemes differ in the errors and failure modes associated with their implementation (summarized in Table 1 using Albert’s approach). These errors and their detection are the primary focus of our study. Practically, our study will focus on transfer errors due to solubility factors and will use, as previously mentioned, a mix of lycopene in DMSO (at varying concentrations) as a stressor. Special attention will also be paid to column to column transfers with the geometric scheme (where errors may propagate and compound). To simplify matters, we assumed that contamination errors were negligible thanks to our choice of tips.

**Table 1:** Possible Source of Errors (after Albert’s Classification)

	geometric scheme	linear scheme
source of error		
volumes transferred (depends on platform)	only large (safe) volumes	any volume in theory. Increments chosen so only safe volumes transferred
contamination by falling droplets (depends on choice of tips and dispensed liquid)	minimal. Only the first column is constructed by direct transfer. Column by column transfers are short.DMSO was dispatched first to minimize risks	possible. The linear dilution scheme involves a lot of travels between wells and reservoirs—each with its own probability of causing contamination with falling droplets. Same safety measure for DMSO
reservoir lycopene concentration	potentially severe. Same as for the linear scheme will only affect column 1.	potentially severe. At high concentration, the mix is expected to be difficult to dispense, affecting all wells in columns 1–10
liquid homogeneity (between wells)	potentially severe. With the iterative transfers, most wells are used as sources. Pipetting precision may be affected by a one-size-fits-all approach for the aspiration and dispensation parameters.	irrelevant. Only two homogeneous reservoirs are dispensed from. Aspiration and dispensation parameters can be set up globally.
type of error		
independent errors	yes	yes
	contamination	contamination
	direct transfers in first column	all direct transfers
correlated errors	yes	none expected
	transfer column by column propagates errors	

### Testing Mix—Lycopene in DMSO

The second component of our study was the stressor we applied: the mix of the solvent dimethyl sulfoxide (DMSO) and lycopene. Personal communication between members of SynbiCITE’s London Biofoundry described the mix of lycopene and DMSO as difficult to pipet manually—going as far as likening the mix to tomato puree at higher concentrations. Albert^41^ calls such mixes atypical liquids. They offer substantial challenges to automate their dilution (and opportunities to determine practical dos and do nots regarding automation of dilution protocols).

The combination of lycopene and DMSO was also chosen for its significant practical applications. Lycopene is a bright-red pigment, belonging to the family of the carotenoids that is found in many plants and organisms.^42,43^ It has major industrial applications (e.g., as a coloring agent). Thanks to their antioxidant properties, carotenoids are in high demand in the medical and pharmaceutical industries.^44,45^ Consumption of lycopene-rich foods has been linked to a reduction in risk of heart disease^46^ and some types of cancer.^47^ Lycopene is not produced by the human body and must be obtained through diet or supplements. Its chemical synthesis is limited by high cost, low yield, and quality.^48^ Microbial production of lycopene has been shown in various hosts^42,43,49^ but is challenging, due to high metabolic burden^50^ and toxicity.^45,51^

The absorption spectra of carotenoids share a similar distinctive shape, with three separate peaks, in the visible part of the spectrum.^52^ Carotenoids interact to various degrees with solvents, resulting in shifts and changes of intensity of the absorption peaks. The spectrum of lutein is almost independent of solvents at various concentrations, unlike zeaxanthin, lycopene, and β-carotene.^53^ The three peaks of the spectrum of lycopene have been located at 457, 483, 516 nm in hexane,^54^ 448, 474, 505 nm in acetone, and 451, 479, 511 nm in DMSO.^55^ The shape of the absorption spectrum and location of its peaks will be used for validation at several points of our study. Lycopene undergoes two types of changes during processing and storage: isomerization from all*-trans* to mono or poly-*cis* forms and oxidation. Due to its highly unsaturated nature, it is very susceptible to oxidation. Degradation is largely affected by time, reaction medium, and environmental conditions, such as heat, light and oxygen.^55−58^

Lycopene is a hydrophobic molecule. Conventional methods for extraction of lycopene use nonpolar organic solvents,^59^ such as hexane, dichloromethane, and ethyl acetate.^60,61^ However, USFDA regulations state that use of organic solvents, such as hexane and methanol, should be limited because of their toxicity. They are also highly volatile, which makes them cumbersome for high-throughput applications. Previous work at the London Biofoundry^62^ used high-performance liquid chromatography to measure lycopene produced and used acetone for extraction. Researchers reported difficulties in maintaining a constant volume during the processing of the samples on 96-well plates. This has led some researchers to use dimethyl sulfoxide (DMSO) instead, as it offers a better trade-off between high throughput and process consistency.^63^ DMSO is a clear, colorless to yellowish viscous liquid with a characteristic bitter odor and taste.^64,65^ It has a proven ability to enhance the permeability of lipid membranes.^66^ It is extensively used in biochemistry and cell biology,^67^ e.g., to extract lycopene from tomatoes, algae, and bacteria.^68,69^

Few direct mentions of serious solubility issues of lycopene in solvents like DMSO could be found in the literature to back our internal communications. However, a study^53^ of how solvents affect absorbance spectra of carotenoids pointed out the relationship between carotenoid concentration and absorbance could become highly nonlinear and presented “differences in solubility leading to the formation of microcrystals” as a possible explanation.

### Overview of the Results of the Study

Our study was designed to be conducted in two parallel tracks—one for each dilution scheme. Each track comprised three successive phases building on top of each other—detailed below. A data analytics phase aiming at developing methods for the assessment of dilution errors was inserted between the first two phases.

- Phase 1: preliminary investigation: testing runs were conducted using food colorant in water or DMSO, then with two mixes of lycopene in DMSO of low (0.005 mg/mL) and high (0.05 mg/mL) concentrations. All lycopene mixes were derived from an extremely concentrated mix of lycopene in DMSO (concentration 0.5 mg/mL) by one or two 1/10 dilutions, see Methods. The high concentration mix exhibited nonlinearity in the relationship between dilution and measurements. Spectral absorbance data were collected (see Methods) and used (a) to assess their suitability for the construction of the calibration curve; (b) to validate the automated implementations; and (c) to probe the spatial patterns introduced to the dilution schemes. The suitability of the dilution scheme was appraised at the outcome of phase 1.
- Phase 2: analysis of the properties of lycopene in DMSO: further runs were conducted to investigate the properties of lycopene in DMSO, including its stability (variation of the absorbance spectrum and degradation studies).
- Phase 3: final measurement study: a final data acquisition protocol, incorporating all relevant information from the previous phases, was designed. Finally, the calibration curve for lycopene in DMSO was to be constructed.

Main results for both tracks are summarized in Tables 2 (Linear Dilution Scheme) and 3 (Geometric Dilution Scheme).

**Table 2:** Main Results for the Geometric Dilution Scheme

geometric (iterative) dilution scheme
phase 1: preliminary study
exploratory analysis: testing runs—food colorant in water
Figures 2, 3, and S3–S16 (Supporting Information, Section 2)
implementation validation: comparison manual vs automated
Figures S17 and S18 (Supporting Information, Section 2)
exploratory analysis: testing runs—lycopene in DMSO
Figures S23–S28 (Supporting Information, Section 3)
effects of iterative dilution
Figures 4; S19–S22 (Supporting Information, Section 2)
Figures S29–S32 (Supporting Information, Section 3)

**Table 3:** Main Results for the Linear Dilution Scheme

linear (direct) dilution scheme
phase 1: preliminary study
exploratory analysis: testing runs—food colorant in DMSO
Figures 5; S33–S37 (Supporting Information, Section 4)
stratified analysis: testing runs—food colorant in DMSO
Figures S38 and S39 (Supporting Information, Section 4)
analytics: development of error assessment methods
Figures 6;Table 4; Figures S40–S44 (Supporting Information, Section 5)
phase 2: lycopene in DMSO—analysis
development runs—lycopene in DMSO
Figures 7 and 8; Table 5; Figures S45–S49 (Supporting Information, Section 6)
stability study—lycopene in DMSO
Figure 9
degradation study—lycopene in DMSO
Figure S50 (Supporting Information, Section 6)
phase 3: final study—construction of the calibration curve
development of repeat filtration method
Figures 10 and S51 (Supporting Information, Section 6)
calibration curve—lycopene in DMSO
Figure 11; Tables 6 and 7

## Results

### Phase 1: Geometric Scheme—Preliminary Investigation

A preliminary study aimed at validating our implementation was conducted.

**Figure 2:**
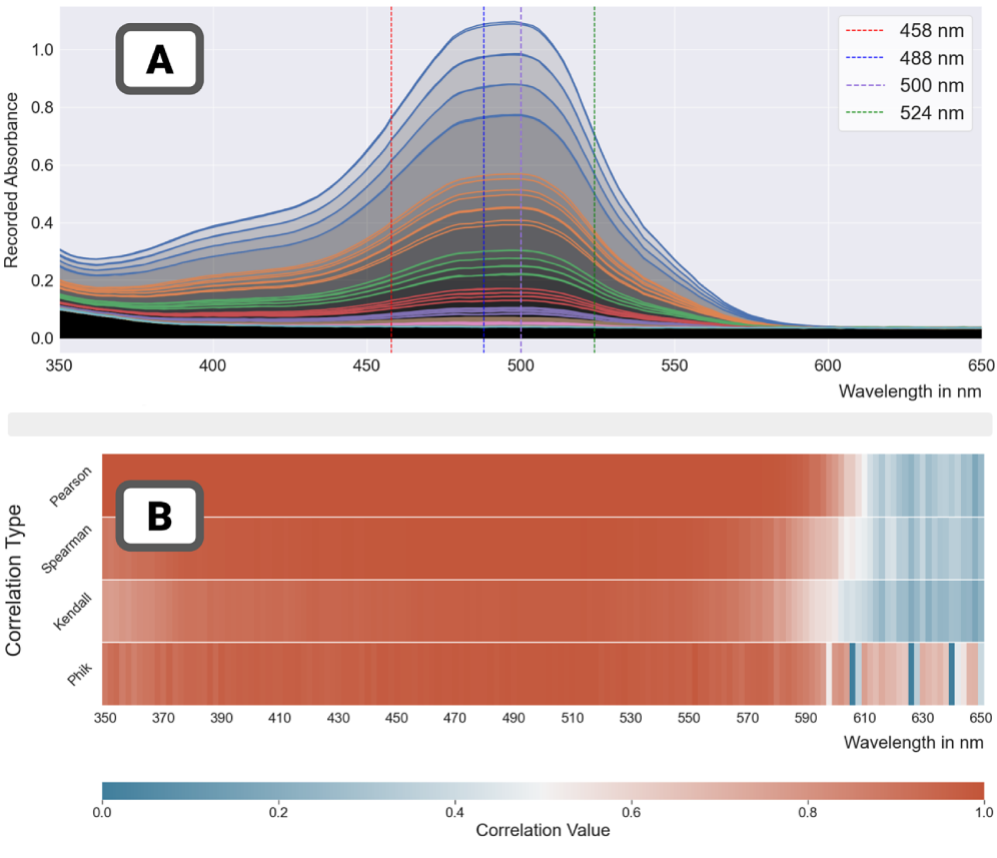
Run 55 – plate 01: food colorant in water—automated implementation of the geometric dilution scheme. (A) Absorbance spectra for all samples (for wavelengths ranging from 350 to 650 nm). Samples are colored according to their rows (blue = column 1, orange = column 2 etc.). (B) Correlations between the target dilution ratios and measurements at all wavelengths. All four common measures of correlation (Person, Spearman, Kendall, and Phi-k) are computed.

**Figure 3:**
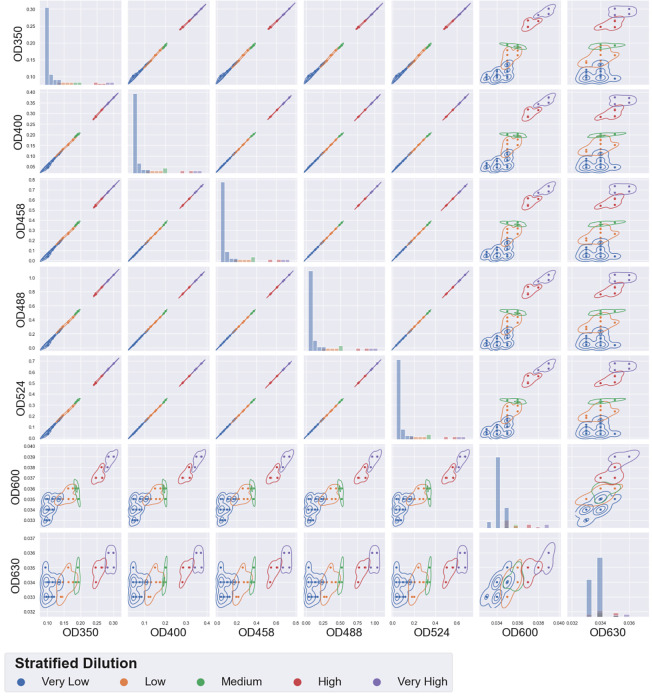
Run 55 – plate 01: food colorant in water—automated implementation of the geometric dilution scheme—pairplot for selected wavelengths. Samples were stratified and assigned colors on the basis of their dilution (very low: dilutions less than 0.2, ... very high: 0.8 and above). Measurements at different wavelengths were highly correlated for wavelengths below 550 nm—measurements from 600 nm and above had low intensity, were noisy, and consequently, their correlations with other measurements were poor.

First, we tested that the collected data were suitable for our application, which we did by looking for a strong positive correlation between the measurements and the dilution ratios and that relation holds over a large range of wavelengths. Figures 2 and 3 show the outcome of this investigation for a development run using a mix of food colorant in water (run 55 – plate 01). The plate was generated with the automated version of the geometric protocol. The analysis proceeded as follows:

- (Subjective) Visual confirmation: the absorbance spectra are plotted for all 80 samples (Figure 2A). Repeats yield very close measurements for the first columns and get more separate as the columns move to the left. Due to the distribution of dilutions generated by the dilution scheme, samples clustered close to a baseline.
- Correlation analysis measurements and dilutions: Figure 2B shows the correlations between the targeted dilution ratios and measurements at wavelengths between 350 and 650 nm. Four correlation metrics (Pearson, Spearman, Kendall, and Phi-k) were computed. All indicate a high degree of correlations between 400 and 550 nm.
- Correlation analysis—between measurements: Figure 3 shows, for selected wavelengths between 400 and 550 nm, a very high degree of correlation. Measurements from 600 nm and above had not only low intensities but also were noisy, indicating that the limits of the instruments were reached.

Altogether, this set of results indicated that the data were highly suitable for the construction of a calibration curve, since they met our expectations.

To further validate the automated implementation of the geometric dilution scheme, a comparison with the manual implementation was conducted. Two of the development runs (runs 55 and 57) contained a plate (plate 01) generated with the automated implementation, and another (plate 02) generated manually—both from the same mix freshly made on the day. Both assays were run by the same experimentalist (A.C., who ran all the assays, manual and automated, in this study), with the same pipetting equipment. Results can be found in the Supporting Information, Section 2. They show measurements for manually and automatically generated plates matching each other to a very high degree.

The process was repeated for mixes of lycopene in DMSO (runs 58–64), using two sets of concentration. Results of the exploratory data analysis for some of these runs can be found in the Supporting Information, Section 3. They were similar to the results for the testing mixes, but correlations between measurements (and with the dilution ratios) were less strong. Absorbance spectra between the runs seem to vary significantly too—this was attributed to the mix age in a later part of our study. These preliminary results confirmed initial assumptions about the difficult nature of the combination of lycopene and DMSO, but also confirmed the data as suitable for our application.

**Figure 4:**
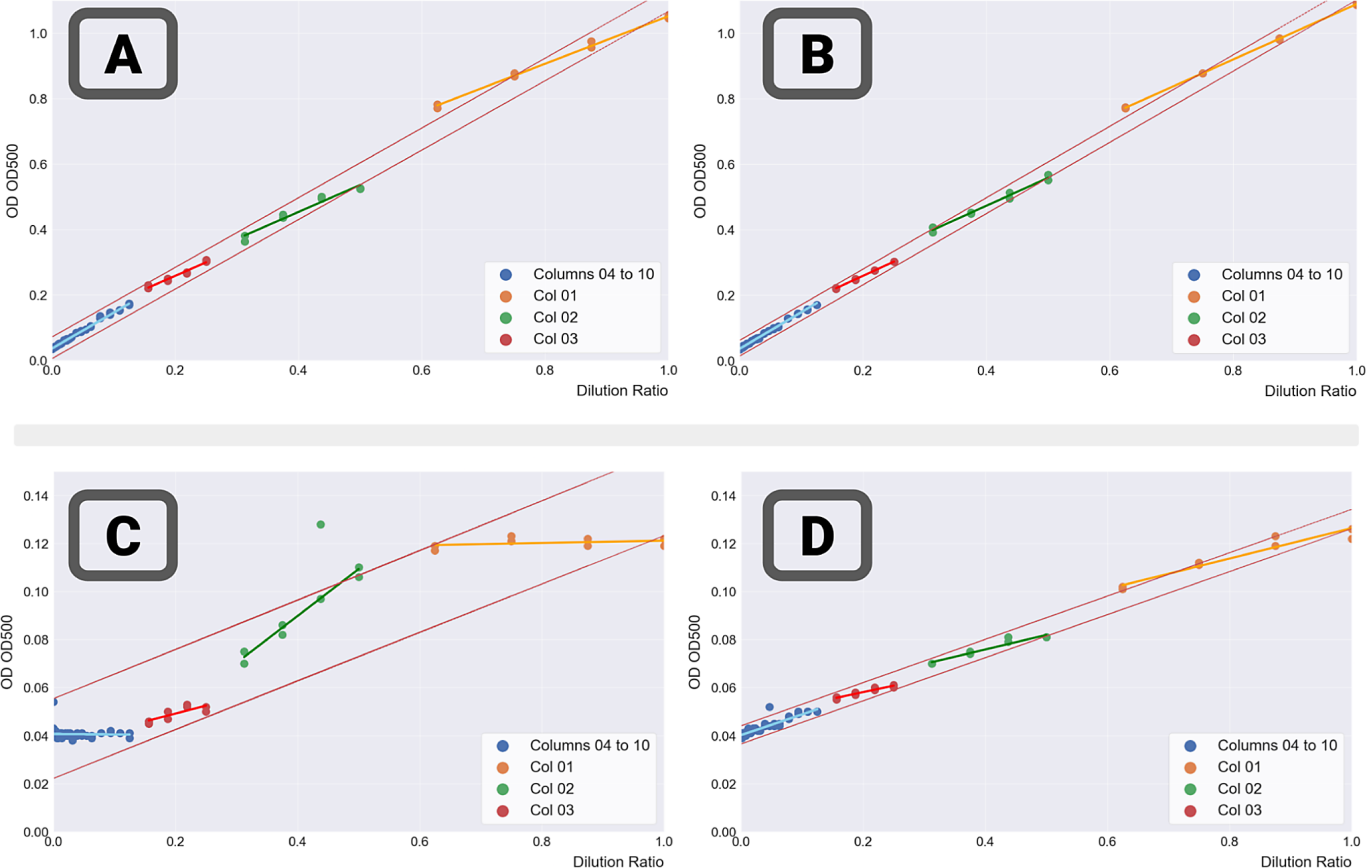
Effects of the iterative dilutions at 500 nm—stratified by columns. (A) Run 55 – plate 01: food colorant and water—automated implementation.(B) Run 55 – plate 02: food colorant and water—manual protocol.(C) Run 62 – plate 01: lycopene and DMSO—automated implementation.(D) Run 63 – plate 01: lycopene and DMSO—manual protocol. In all plots, the samples were colored according to their rows, and the data set trend was indicated by the confidence intervals (in red) obtained with a linear regression for the whole data set. All measurements, at the wavelength 500 nm, were conducted on the same platform (CLARIOstar A in our foundry).

After validating the data, we investigated the effects of the iterative transfers from column 1 to column 2 (2 to 3, etc.), as it was the operation that was identified as the most liable to introduce correlated errors. Accurate transfer from one column to the next would result in measurements that are distributed tightly around the regression curve and columns showing trends that deviate little from the general trend of the data set. Figure 4 shows results for two runs—one run with food colorant in water (run 55 – Figure 4A,B, representative of this mix) and one for lycopene in DMSO (run 63 – Figure 4C,D). For both runs, a plate was generated with automation (Figure 4A,C) and manually (Figure 4B,D). All measurements were conducted on the same measurement platform. For all data sets, the measure of trend is indicated by the confidence intervals (in red) obtained with a linear regression.

For the food colorant in water (Figure 4A,B), stratification by column reveals slightly different trends (slopes and baselines) for columns 1, 2, and 3. A few samples fell outside the confidence interval of the predictions. This was true for both manual and automated implementations and was stronger with automation. Results are consistent with a transfer that is too comparatively large from one column to the next. The difference between automation and manual implementations was very noticeable for lycopene in DMSO (Figure 4C,D). Results for the manual implementation (Figure 4C) were much smoother and closer to the runs with water and food colorant than the automated results (Figure 4D). They still exhibited the same jumps from one column to the next and the slope pattern as in Figure 4A,B, but overall the deviations were smaller. Results for several other automated runs (see Supporting Information, Section 3) with lycopene and DMSO (at high and low concentrations) show similar results—large deviations from the data set trend, very different slopes for each column, but also large deviations in the trends for the same columns—all indicative of large errors in the transfers from adjacent columns, and a general lack of control of the operation.

Taken in conjunction with the results for the runs with food colorant, we concluded that two factors were at play. First, iterative dilution introduces a distinct class of (correlated) errors. Even in the case of a simple liquid mix, the errors were visible, so much so that data had to be analyzed column by column in order to account for them. Second, and as expected, the errors were significantly worse for the mix of lycopene in DMSO. For both reasons, the geometric (iterative) dilution scheme was concluded to be too cumbersome for the construction of the standard of lycopene in DMSO, and further investigations were canceled. The issue of controlling dilution errors with the geometric (iterative) dilution scheme is revisited in the conclusion to this paper.

### Linear Dilution Scheme—Phase 1: Preliminary Investigation

**Figure 5:**
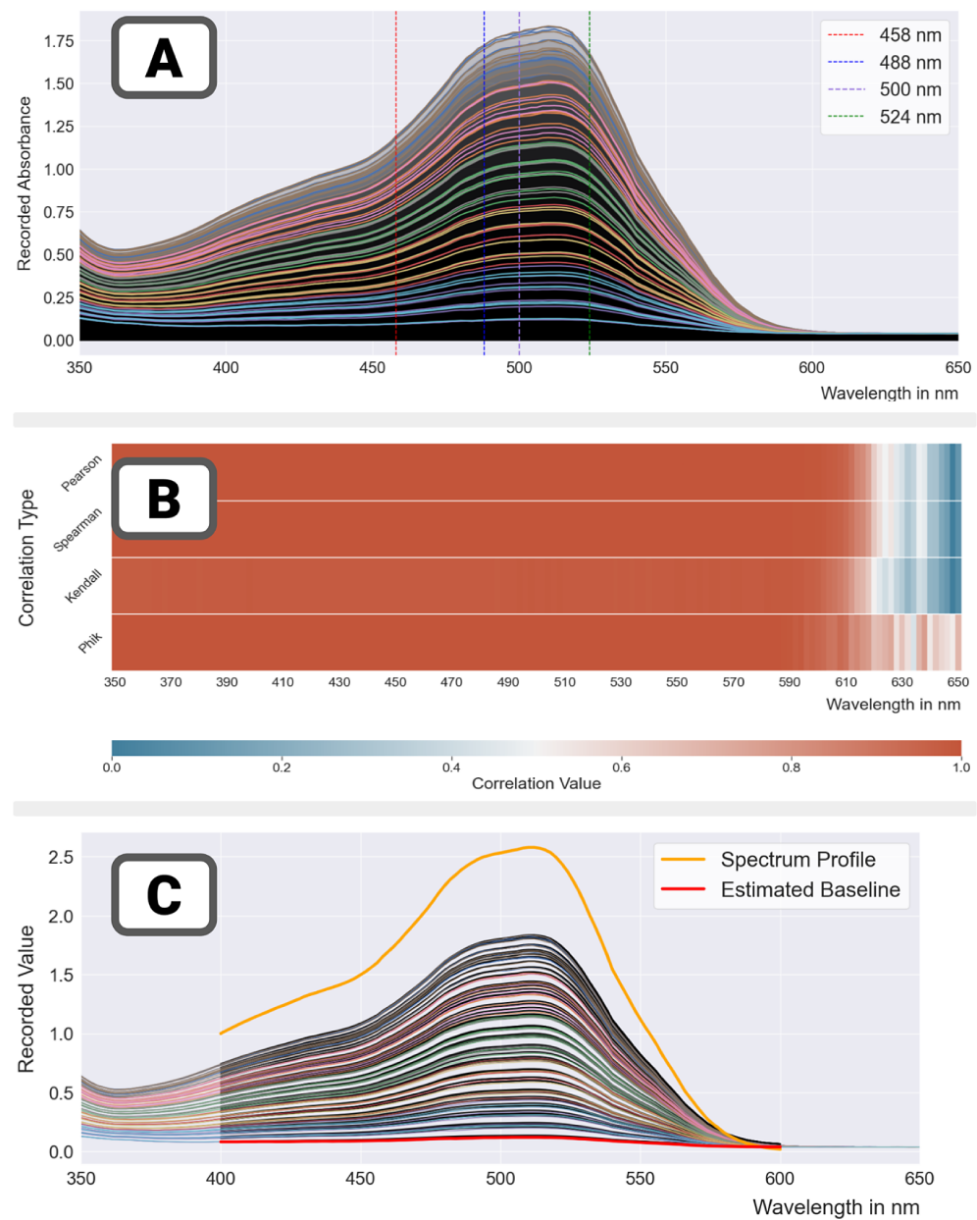
Run 074 – plate 01: food colorant in DMSO—linear dilution scheme. (A) Absorbance spectra for all 80 samples in the plate.(B) Correlations between the target dilution ratios and measurements at all wavelengths.(C) Estimation of the spectrum of the mix and the baseline reading—the spectrum is normalized for legibility purposes.

The same type of preliminary study was run as for the geometric scheme, with the same aim to validate our implementation and assess the general suitability of the collected data for our application. The preliminary study used once again food colorant, but this time in DMSO. Figure 5 shows the main results for run 74 – plate 01. Results are representative of all development runs (see Supporting Information, Section 4 for more runs). Measurements were repeated in two different CLARIOstar platforms (A and B in our naming nomenclature, A being shown in Figure 5) with almost identical results (see Supporting Information, Section 4). Unlike the geometric dilution scheme, stratified analysis by column and by row showed no discernible differences between the trends for the columns, rows, and the general trend of the plate (see Supporting Information, Section 4).

Absorbance spectra for all 80 samples are shown in Figure 5A. For all concentrations, repeats were close to each other and their magnitude was evenly spread, with a slight compression indicating some nonlinearity at the highest concentrations. This was confirmed by the analysis of the correlation between the targeted dilution ratios and measurements (Figure 5B). As a consequence of our choice of sample concentrations (evenly distributed between 0 and the maximum concentration in the solute reservoir), the pattern of correlations was very pronounced (almost perfect correlation at wavelengths below 580 nm). The same applies to correlations between measurements at different wavelengths (Supporting Information, Section 4), which were highly correlated for wavelengths up to 580 nm.

The high degree of linear correlation between measurements at different wavelengths was exploited to construct a model (called SM, for spectrum model), which binds measurements over a range of wavelengths to a vector *S*—the spectrum of the solute and a baseline *B*—the background measurement of the plate over the same range. The coefficient α_*i*_ is well dependent and indicates the quantity of signal producing-solute in the well. The model imposes minimal constraints: all components of *S* and *B* and coefficients α_i_ are to be positive. A penalty function is used so the baseline *B* stays close to blank values.

Figure 5C shows the results of the estimation of the parameters *B*,*S* (α_*i*_ not shown) from the plate data. The baseline *B* was plotted in red and matches the absorbance spectra of the blanks on the plate. The spectrum *S* was plotted in amber; for legibility purposes, its amplitude was normalized so that its value is 1 at wl = 400 nm (Supporting Information, Section 5)

### Analytics—Error Assessment Methods

**Figure 6:**
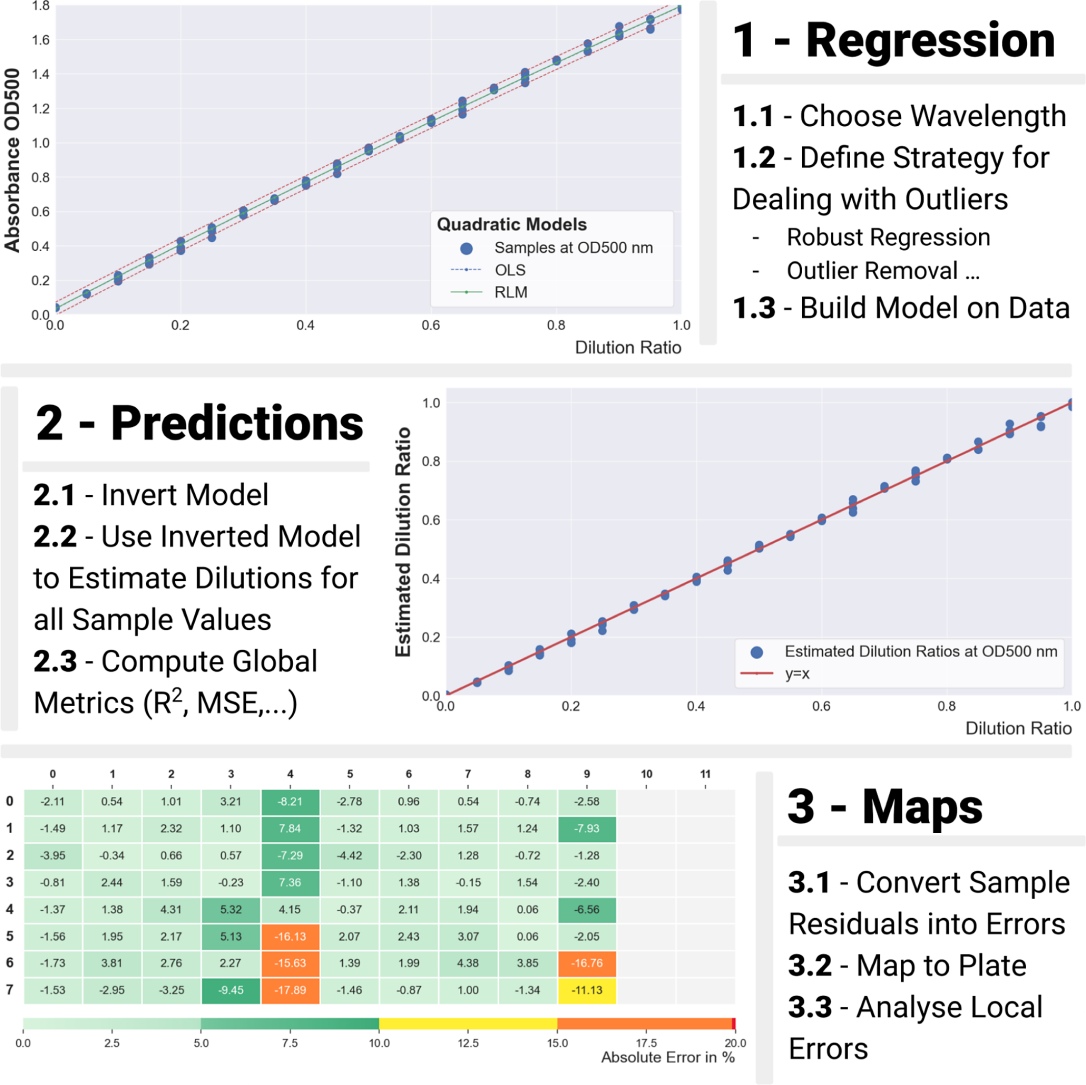
Error quantification workflow. The workflow is illustrated on run 074 – plate 01: food colorant in DMSO (linear dilution scheme).

Ideally, the content of wells postdilution would be known to a sufficient precision—either because liquid-handling makes small errors (which would not be the case in our study) or because the dispensed volumes can be measured accurately (which was not possible). Estimating dilution errors therefore needs an alternative way to estimate the concentration of the mix in the wells. We developed the following workflow (Figure 6), built on regression modeling. Our workflow proceeds as follows:

Step 1 – regression: a wavelength containing information representative of the concentration of solute is selected. Good practices suggest it be located near a peak of the spectrum, where the signal is largest. Then, a regression model relating the independent variable (the targeted dilution ratio or the concentration) and the dependent variable (the absorbance measurement) is built.

Step 2 – inversion: the regression model is inverted and used to estimate the concentration for each sample (or equivalently the dilution ratio). This is where the framework deviates from simple regression to focus on estimation of liquid-handling errors. Without liquid handling errors, the relationship between estimated concentrations and targeted concentrations would perfectly fall on the *Y* = *X* line. In practice it does not, and (global) discrepancy metrics can be computed including the common Pearson correlation and root-mean-square error (RMSE, concerned with the magnitude of errors).

Step 3 – maps (local error analysis): the final step is concerned with the detection of local patterns. For all samples, the residual error between predicted and targeted concentrations is calculated and converted into relative errors (by dividing the residual by the targeted value). Finer-grain analysis (e.g., stratification against the targeted concentration) and analysis of local effects may follow.

The framework is open with regards to which regression model is used in the first step. In our study, all trends could be captured by linear models of degree 1 (affine models) or 2 (quadratic models, to capture a possible curvature indicative of saturation). The framework is also open with regards to how to deal with outliers. For every data set, linear regression was performed with the whole data set to get an insight into its trend and benefit from the set of statistics yielded by linear regression techniques—in particular, the confidence intervals on the predictions and on the parameters. Then, robust regression models were used (which adapt the weight of the sample data to mitigate the influence of outliers) as a refinement.

The error-estimation workflow can also be adapted to use the estimated coefficients α = (α_*i*_) in the spectrum model instead of the measurements at the selected wavelength. The precision of the overall approach will then be very dependent on the validity of the spectral model. For high concentrations of solutes, various solubility effects may occur and only a portion of the spectra may be sufficiently correlated.

Figure 6 illustrates the different steps of the framework on run 074 – plate 01. Results after inversion show an extremely tight match between estimated and targeted concentrations. Table 4 shows nearly perfect global quality metrics (Pearson, Spearman correlations, and RMSE). Mapping relative errors onto the plate shows that the largest relative errors are for the smallest concentrations of solute (5–15%); they are consistent with manufacturers statements of a few μl errors. Results also show nearly identical results between using the spectrum method and measurement data at 500 nm (close to the peak of the spectrum for the testing mixes).

**Table 4:** Global Quality Metric Values^a^

run 074 – plate 01		
	model of order 1	model of order 2
At 500 nm		
Pearson correlation	0.9992	0.9994
Spearman correlation	0.9976	0.9974
RMSE	0.0130	0.0111
Spectrum Method		
Pearson correlation	0.9992	0.9994
Spearman correlation	0.9969	0.9969
RMSE	0.0132	0.0109

a All results are for the development run 074 – plate 01 (food colorant in DMSO—linear dilution scheme). All values have been rounded down to four decimal places

### Linear Dilution Scheme—Phase 2: Lycopene in DMSO

**Figure 7:**
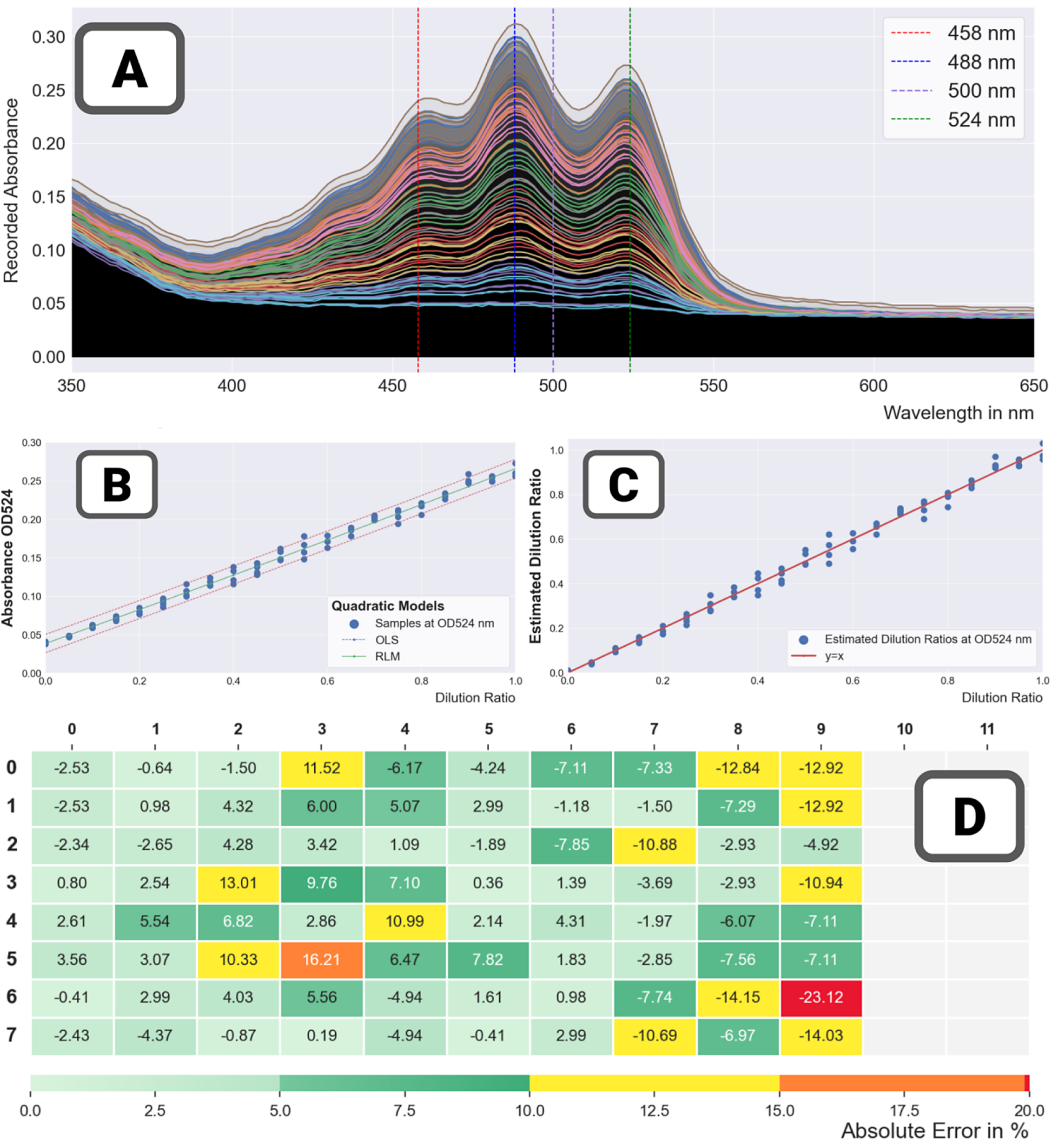
Results for the typical run 081 – plate 01 (low concentration mix). (A) Absorbance spectra for all 80 samples in the plate. (B) Correlations between the target dilution ratios and measurements at 524 nm (peak of spectrum). (C) Estimation of the real dilution ratios (the *Y* = *X* line is plotted in red for comparison). (D) Relative errors (in percent) for all samples mapped onto the plate.

**Figure 8:**
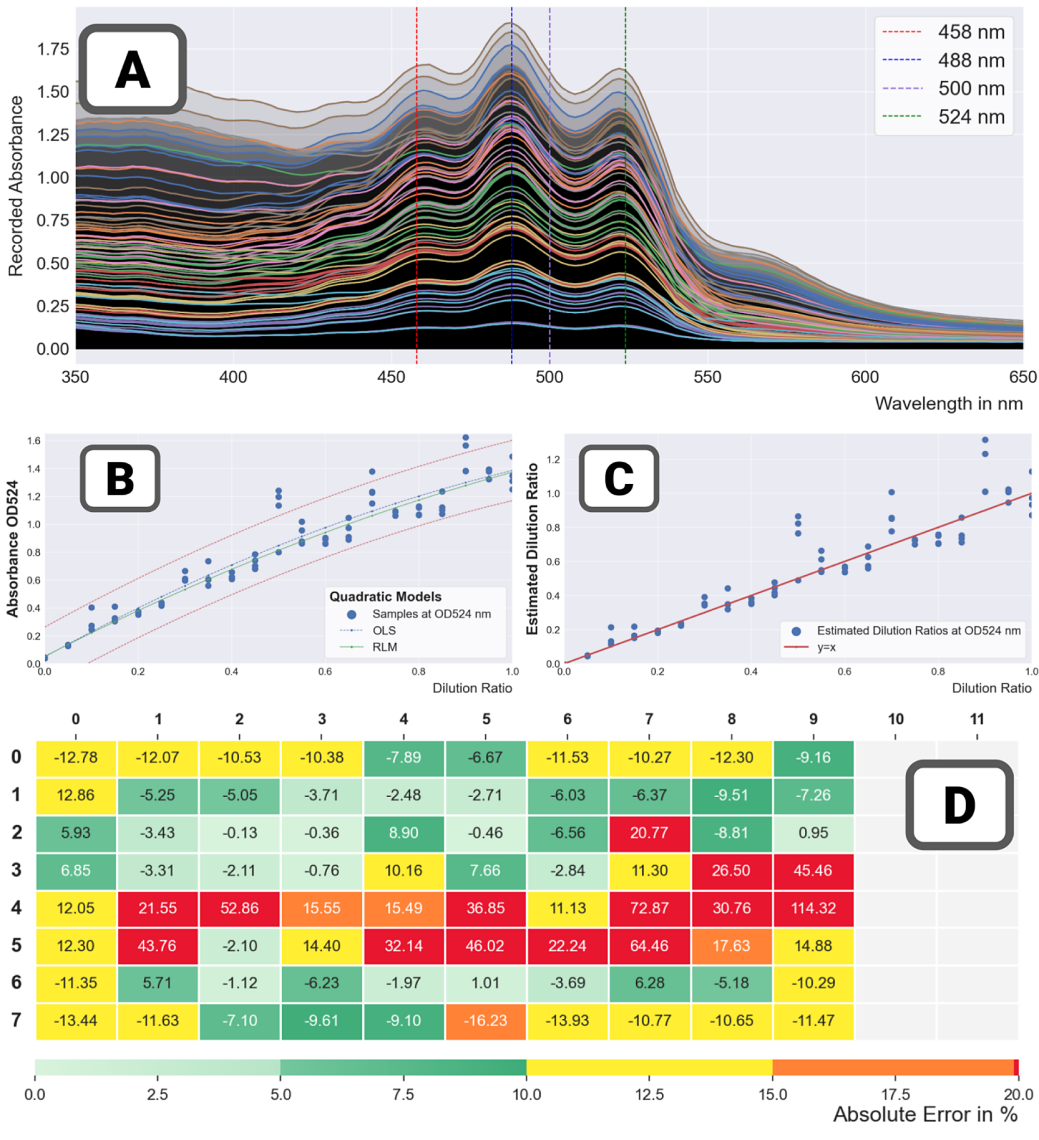
Results for the typical run 079 – pplate 01 (high concentration mix). (A) Absorbance spectra for all 80 samples in the plate. (B) Correlations between the target dilution ratios and measurements at 524 nm (peak of spectrum). (C) Estimation of the real dilution ratios (the *Y* = *X* line is plotted in red for comparison). (D) Relative errors (in percent) for all samples mapped onto the plate.

Several runs for the linear dilution scheme were conducted to tune up the protocol and test the error assessment framework (see Supporting Information, Section 6) using both low concentration and high concentration mixes. Results for the run 81 (low concentration mix) and run 79 (high concentration mix) are shown in Figures 7 and 8, respectively. Both used freshly made mixes (starting from an original mix at 0.5 mg/mL) and were representative of runs under such conditions. Table 5 contains the global quality metrics for both runs.

**Table 5:** Global Quality Metrics Values—Lycopene in DMSO^a^

Run 081 – Plate 01		
	model of order 1	model of order 2
At 524 nm		
Pearson correlation	0.9966	0.9971
Spearman correlation	0.9955	0.9955
RMSE	0.0257	0.0252
Spectrum Method		
Pearson correlation	0.9966	0.9967
Spearman correlation	0.9946	0.9946
RMSE	0.0270	0.0266

Run 079 – Plate 01		
	model of order 1	model of order 2
At 524 nm		
Pearson correlation	0.9686	0.9605
Spearman correlation	0.9682	0.9682
RMSE	0.08589	0.1009
Spectrum Method		
Pearson correlation	0.9515	0.9377
RMSE	0.1111	0.1345

a Top: run 081 – plate 01 (lycopene in DMSO – low concentration mix). Bottom: run 079 – plate 01 (lycopene in DMSO – high concentration mix). All values have been rounded down to four decimal places.

The absorbance spectrum of run 81 (Figure 7A) displayed the characteristic shape of the carotenoids, with three distinct peaks at 458 nm, 488 and 524 nm, which were slightly higher values than reported in the literature. Measurements at the peak wavelength of 524 nm (Figure 7B) were dotted around the regression curve (all remaining in the confidence interval of the regression) but less tightly than for the food colorant (Table 4). Likewise, and as expected, the inverted model did not yield as good some estimations of the concentrations as for the food colorant. Quantitative results (Table 5) showed errors twice as large as with the food colorant mix (RMSE was twice as large as that of run 74 in Table 4), but still very good fits. When mapped onto the plate, some wells displayed hefty relative errors of 10–15% (for the lowest dilution ratios). A clear negative correlation between dilution ratios and relative errors could also be observed, again as expected.

The absorbance spectrum of run 79 (Figure 8A) still displayed the characteristic shape of the carotenoids, with its peaks still at 458 nm, 488 and 524 nm, but was flatter than for the previous mix). Measurements at 524 nm (Figure 8B) were much more dispersed around the regression curve (some samples being outside the already wide confidence interval of the regression). When a robust regression model was used, the regression captured the trend of most of the points in the data set and overlooked the outliers. This dual behavior was reflected in the estimations (Figure 8C) and on the plate map (Figure 8D) where some wells displayed very large relative errors. As with the low concentration mix, a clear negative correlation between dilution ratios and relative errors could be observed but stronger. Quantitative results (Table 5) showed significantly larger errors than with the low concentration mix (RMSE four times as large). Measures of correlation were still high, which is more a consequence of the spread of concentrations afforded by the linear dilution scheme. Overall, the high concentration mix yielded noisier data, indicative of imprecise liquid transfer.

In addition to the analysis of liquid-handling errors these runs (and the runs for lycopene-DMSO mixes using the geometric dilution scheme) were checked for spectrum stability. The absorbance spectrum of the mix is a strong indication of the state of the mix, so replicability requires such stability.

**Figure 9:**
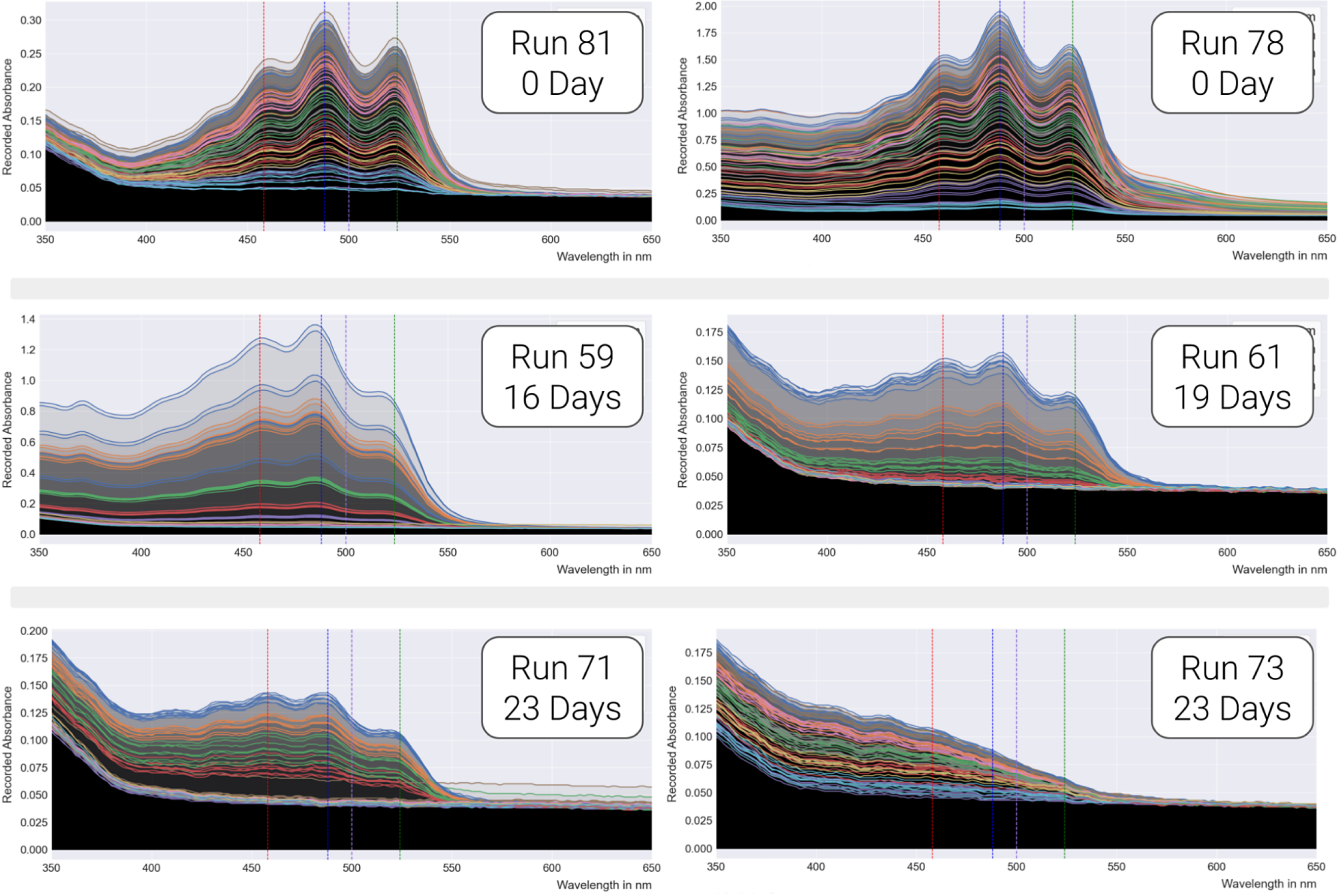
Spectral jungle of lycopene in DMSO.

For each assay, the absorbance spectra for all 80 samples in the plate are plotted. The age of the mix used was estimated as the number of days between the creation of the original mix and the date of the assay. The variety present in the absorbance spectrum (Figure 9) was a surprise. It was expected that mix concentration would affect the spectrum (as in Figures 7 and 8). But the influence of the age of the mix was not expected to be so pronounced after we took the precaution to store all mixes in the dark in a fridge. Older mixes (2 weeks and older) lost their distinctive peaks. Conversely, fresh mixes yielded similar absorbance spectra (albeit subject to the effects of mix concentration). Fresh mix preparation was therefore concluded to be imperative to the collection of replicable results.

Before conducting the final study, a degradation study was run. Its purpose was 2-fold: (a) to determine a safe time scale for the processing of the plates and their measurements, which in turn helps decide the number of technical repeats (the more repeats the better in general, provided the mix retains its properties while the measurements are conducted) and (b) to give us further insight into the aging effect of the mix. The degradation study was conducted on several plates with both low concentration mixes and high concentration mixes, since concentration was a factor in the appearance of the absorbance spectrum. The study consisted of monitoring a plate over 12 h and measuring every hour the entire absorbance spectrum (350 to 650 nm). All assays used fresh preparations—less than an hour old to remove all prior aging effects—and were run at room temperature, see Supporting Information Section 6 for the detailed results.

### Phase 3: Lycopene in DMSO—Final Study

Findings from all previous phases were incorporated into a final protocol (see Supporting Information, Section 6). The protocol comprises three steps—each step was designed to account for an identified source of variability (variation between batches, differences in the treatment of the plates, and finally, variability between measurements).

To generate the final data for the construction of the standard curve, a final series of runs were conducted. Given the previous results with lycopene in DMSO, we decided to change the concentration of the higher concentration mix (instead of a 1/10 dilution, it was obtained by a 4/100 dilution). This way it would overlap better with the lower concentration mix.

Also for each plate, a high number of technical repeats were run. This was used for a final investigation of the outliers observed with high concentration mixes in the previous phase and to assess whether the outliers could be explained as simple instrument issues, by temporary phenomena, which would disappear over a practical time scale (e.g., temporary solubility issues), or by more permanent issues. Results (Supporting Information, Section 6) showed a more complex phenomenon. Successive readings for some samples went on a “journey”—many regressing toward the trend of the data set from the outset, some deviating (over a few measurements) before returning to the trend. A few did not return to the trend over the time scale of the measurements. The phenomenon was more marked for high concentration mixes. Since the traveling samples were not located in identical positions, instrument malfunctions could be ruled out. We believe that such behavior is indicative of solubility issues in the wells—inhomogeneities agglomerating, moving, and interfering with the absorbance measurements.

A possible way to control these transient effects is to mix more thoroughly in the wells after dispensation. Instead of further protocol alterations (which were not sure to yield the desired benefits for the high concentration mixes, introduced more parameters to tune, and made the automated protocol less transferable), we decided to rely on data processing instead and developed a methodology that aggregated measurements for the same sample (same well, over several technical repeats) into a composite value less influenced by the fluctuations.

Instead of using statistics such as the median or other estimates of the center of the distribution, which are adapted to nontransient signals, our method selected on the basis of how much the recorded values deviated from the trendline of the data set, and for each well, selected the value that deviated the least. We called the method “repeat filtration”.

**Figure 10:**
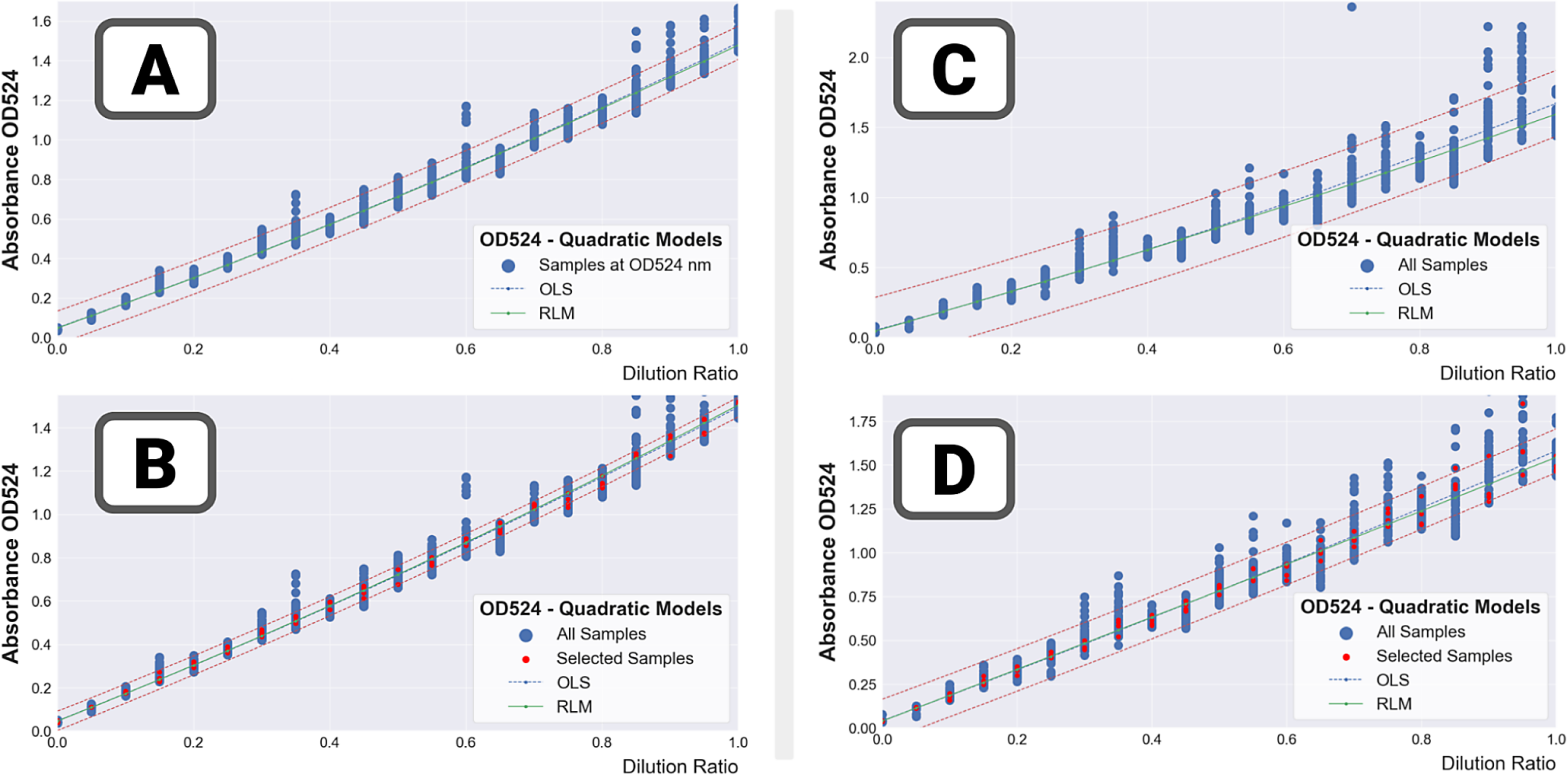
Combining multiple repeat measurements: repeat filtration in practice. The regression curves were computed for two high-concentration mixes (run 90 – left and run 85 – right) with and without the repeat filtration method. (A) Run 90 – 20 repeats – without repeat filtration. (B) Run 90 – 20 repeats—with repeat filtration. (C) Run 85 – 12 repeats—without repeat filtration. (D) Run 85 – 12 repeats—with repeat filtration.

Results in Figure 10 (two runs with high-concentration mixes – 20 technical repeats, spread over two CLARIOstar platform) show the strategy to be highly effective as it returns values, which are close to the trendline, leading to much better quality metrics (RMSE being reduced by a factor of 2 or 3). It is worth noting that not all samples are brought back to the trendline—in the significantly noisier run 85 (Figure 10C,D), some of the samples for the highest dilution ratios remain very far off trend (either due to very high solubility issues, or failed liquid transfer).

**Figure 11:**
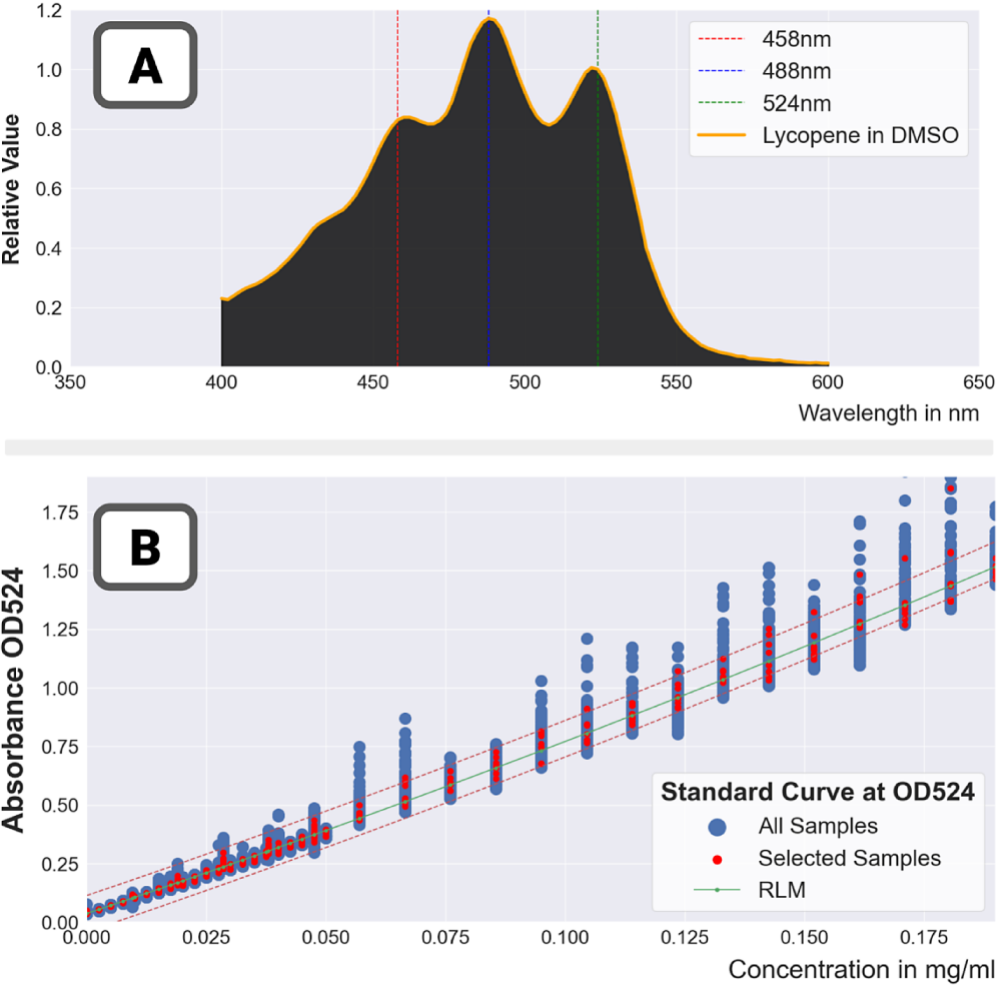
Spectrum of lycopene in DMSO and calibration curve at 524 nm. (A) The spectrum of lycopene in DMSO—its magnitude was normalized to be 1 at the 524 nm peak.(B) The final calibration curve generated from several runs at low and high concentrations. For each run, the repeat measurements (plotted in blue) were first combined (the combinations are plotted in red), and then the calibration curve was generated by running a final regression with all the combined data.

Finally all the results for the assays with a high number of repeats were aggregated to estimate the absorbance spectrum of lycopene and construct the final calibration curve (Figure 11). The absorbance spectrum exhibits the well-known three peaks (located at 458 nm, 488 nm, and 524 nm). Tables 6 and 7 show the equations of the calibration curve at the 524 nm peak. Both linear and quadratic models were used (showing no significant difference). In Table 6, repeat filtration was used; in Table 7, it was not. The returned trend lines were very similar,which was to be expected as robust linear models (designed to exclude outliers) were used. However, the RMSE was reduced by more than a third with repeat filtration.

**Table 6:** Construction of the Calibration Curve at 524 nm with Repeat Filtration

calibration curve (with filtration)		
at 524 nm	model of order 1	model of order 2
Pearson correlation	0.9956	0.96065
Spearman correlation	0.9966	0.9966
RMSE	0.0058	0.0051
model	OD_524 = *f*([lycopene in mg/mL])	
linear model:*f*(*X*) = *bX* + *c*	*b* = 7.2277; *c* = 0.0309	
quadratic model:*f*(*X*) = *aX*^2^+ *bX* + *c*	*a* = 4.3060; *b* = 6.6802; *c* = 0.0389	

**Table 7:** Construction of the Calibration Curve at 524 nm Without Repeat Filtration

calibration curve (without filtration)		
at 524 nm	model of order 1	model of order 2
Pearson correlation	0.9888	0.9911
Spearman correlation	0.9953	0.9953
RMSE	0.0094	0.0081
model	OD_524 = *f*([lycopene in mg/mL])	
linear model*f*(*X*) = *bX* + *c*	*b* = 7.0929; *c* = 0.0312	
quadratic model*f*(*X*) = *aX*^2^+ *bX* + *c*	*a* = 3.7774; *b* = 6.5082; *c* = 0.0400	

## Discussion

The use of automation to construct a calibration curve of lycopene in DMSO is a good example of a deceptively simple problem. No biological variation was involved in the study. Measurements were conducted on well-calibrated devices. Industry-grade liquid-handling platforms were used. Development assays on development mixes such as food colorant with water or DMSO helped validate the implementation of the dilution protocols on our platform. But lycopene and DMSO combine into what Albert et al.^41^ euphemistically refer to as a complex liquid, for which there are few established recommendations for pipetting, and the mix proved to be very challenging to pipet and, unexpectedly, also challenging to measure. In the context of our study, it therefore was an excellent stressor and in particular was very useful to reveal flaws with the dilution schemes we tested and develop methodologies to monitor them. We now discuss the main findings of our study in ascending order of importance and generalizability

### Lycopene in DMSO

The first set of findings is related to the specific mix of lycopene in DMSO. Our study confirmed that the absorbance spectrum of lycopene in DMSO had the well-known three-peak shape that is distinctive of carotenoids—although the peaks were shifted by a few nanometers from previous reports.^54,55^ The degradation study also yielded results consistent with previous studies and confirmed that there was little degradation in the first hour at room temperature. By combining mixes of lycopene and DMSO, we could construct a calibration curve exhibiting a clear linear phase and then saturation—as was our initial goal.

At the same time, important sources of variability were identified that are largely ignored in the published literature, where much is made of denaturation by light and heat.^55−58^ In our study, great care was taken in minimizing these effects when storing the mixes. But even with such precautions, the mixes exhibited a large range of absorbance spectra—some being so flat that their distinctive three peaks would disappear. We postulate that the main driver for the transformation is an aggregation process where molecules of lycopene bind to other molecules of lycopene. Such a process would be concentration-dependent (the more concentrated, the more likely molecules of lycopene are to find other molecules to bind to) and would be compatible with the mentions of microcrystals.^53^ Furthermore, the older the mix, the bigger the clumps of lycopene are—the more denatured the spectrum. Some degradation by oxidation could not be ruled out as a secondary driver but failed to explain the concentration-dependence. Overall such lack of knowledge is not rare, as few negative results or warnings are published—a common complaint in the scientific community.^70,71^

As a consequence, the study introduced a protocol to ensure data quality and consistency. The protocol comprised three levels—each dealing with an identified, confounding source of variation in the data: variation between batches of lycopene mixes (preparation, chemical state), variation between plate treatment and sample processing, and finally variation during measurements. With this protocol, all measurement repeats can be considered either technical repeats or measurements carried out on similar objects (the study equivalent to biological repeats). Even with such precautions, some wells still exhibited transient fluctuations (which we attributed to severe solubility issues), and that led us to develop a novel data set aggregation method (which we called repeat filtration). We believe that this approach is valid beyond our choice of solute and solvent.

### Methodologies for Error Assessment and Quality Monitoring

Quality assessment and monitoring have been at the heart of our approach, and the development of practical monitoring methodologies was an initial deliverable of our study. It is worth restating the context of our study and the related constraints. The study was conducted at the London Biofoundry, SynbiCITE on industrial automated platforms. The amount of data collected was large (several dozens of plates) compared to what a manual study would have collected but still too small for any machine learning approach to be attempted. Only simple statistical techniques (mostly based on regression) were used. This was sufficient to identify the type of dilution errors, estimate their magnitude, and, in the context of a settled workflow, for quality monitoring. Most importantly, the analyses would be conducted plate by plate, which in the context of an environment such as ours is the most practical scale to operate.

We developed a quality assessment methodology based on a regression-error estimation workflow. It builds a regression model for measurements, at a given wavelength or over a range of them, against the targeted dilution ratios (the independent source of variability). Dilution ratios are then estimated, for all wells, by inverting the regression model. An error metric (quantifying how much a data point deviates from the trend of the data set) returns a measure of the overall data set consistency. Individual residuals are used to detect outliers. In the study, the overall error metric was used to quantify the severity of the dilution errors (it was significantly worse for the higher concentration). The maps revealed an expected pattern where relative errors decreased with the dilution ratio but no other localized effect. The regression-error assessment workflow was also adapted to develop the filtering methodology we called repeat filtration. The methodology returns composite statistics from a set of technical repeats and was utilized to deal with transient phenomena (caused by poor solubility of lycopene in DMSO at high concentration in the case of our study). The practical result of its application was a significant reduction of the confidence interval around the calibration curve.

Error metrics, based on internal data set consistency, are popular in process control (variance control especially) but are not without their limitations. Their use is blind to systematic biases in the process (e.g., a consistent 5% bias in volume transfer cannot be detected). Also, they fail if too many samples in the same data set are unreliable (a case never encountered even with the highest concentration mixes). The approach can be applied to other types of assays where the response is driven by a (known, measurable) independent variable. For instance, it could be applied to assays measuring the response of a promoter to the concentration of potential inductor—although in that case the regression model would be a Hill function (and the benefits of linear regression would have to be abandoned). As previously, plate design and a wise choice of value for the independent variable will be important to efficient assessment and monitoring.

Workflow monitoring of course extends beyond consistency within plates, and the relating of measurements to the intended dilution ratio (or any other independent variable): consistency between plates needs monitoring too. In similar contexts to our study, approaches such as meta analysis and quantification of agreement between measures become possible. These complementary approaches are now being pursued at the London Biofoundry, SynbiCITE and will be reported upon in future studies.

### Automation of Liquid Handling

Our study approached liquid-handling and its reliable automation from an unusual standpoint in synthetic biology: vulnerability analysis. It is worth repeating that, to the best of the authors’ knowledge, this is the first time the problem has been approached in this manner.

Our study gave us two important insights into the automation of liquid handling: (a) the general awkwardness of the iterative transfer method; (b) the power of the direct transfer method. Both results are worth discussing in further detail. We believe that they do not only apply beyond our choice of solute and solvent and our exemplar application, but more importantly, that they are evidence that in liquid-handling automation, the KISS principle (“Keep It Simple, Stupid” and variants thereof) should be followed.

The geometric dilution scheme (built on iterative transfers) was abandoned early due to the combination of two factors: (a) iterative dilution introducing non-negligible, correlated errors (between column); and (b) the mix of lycopene in DMSO, making these errors significantly worse. The first factor was unexpected—the second was not.

Initially, we expected the mix of lycopene and DMSO to reveal whether iterative liquid transfers from adjacent columns were error prone. The underlying reasons for these errors would then be (a) the one-size-fits-all nature of the protocol in its assignment of aspiration and dispensation parameters, which would cause issues when dealing with columns of liquid with varying viscosity; and (b) the successive transfers, which would propagate the errors, and compound them. Our data indeed confirmed the presence of large errors (especially at higher concentrations). At the same time, no evidence of contamination by leakage could be observed, which was attributed to a good choice of tips. We did not expect to find any issues with common liquids. Yet, the data showed clear jumps from column to column. If replicated, this result would imply that the popular sequential transfer method should only be used for coarse assays.

Comparison with manual implementation was revealing. One of the greatest advantages of the iterative transfer method is how easy it is to use manually—which we exploited to validate automated and manual protocol implementations. With the training mixes, the data generated with the manual protocols had comparable errors to automation data. But with the atypical lycopene in DMSO mixes, the differences were noticeable—in favor of the manual protocol. We believe that such results will generalize and ill-adapted automated protocols can be expected to generate worse data than manual protocols. We postulate that the cause for the differences is the existence of a control feedback mechanism in liquid-handling among experienced experimentalists when using laboratory micropipettes. This helps them adapt to the liquid being handled (probably through some form of hand-eye coordination). Such feedback is absent in automated applications where the robotic platform rigidly implements a series of instructions—without such a feedback loop. A possible solution would be to adapt the aspiration/dispensation parameters to the content of the wells (ironically for an application such as ours, this would necessitate new calibration curves for these parameters). Another avenue would be to create a feedback loop either with computer vision, or more simply tip sensors (e.g., by using conductive, or liquid sensing, tips). All these potential solutions were rejected for similar reasons: substantial additional complexity (in marked contrast to the initial simplicity of the protocol), reduced portability (the more specialized the solution, the less likely it is to be ported to another setup), and parameter explosion (and its corollary, increased development time and possibly fragility).

Unlike the iterative schemes, the schemes built on the direct transfer approach (such as the linear dilution scheme) proved well-suited to automation. Minor variations related to parameters, such as the aspiration and dispensation speeds, were observed. Mixing “in the well” proved an issue with the high-concentration mixes of lycopene and DMSO, which had outliers that required removal. Mixing in the wells is, in general, more difficult with direct transfer as the wells vary in content and are therefore liable to exhibit separate mixing issues.

The suitability of the direct transfer method is of great importance in the context of automation of liquid handling. Direct transfer is conceptually the simplest approach to liquid-handling, as it relies on one set of instructions only: go to reservoir R, aspire volume *V*_A_, go to well W_ij_, dispense volume *V*_D_. Few parameters need assigning and tuning. For each of the reservoirs, aspiration and dispensation parameters can be set up globally, although mixing parameters may need to be well or concentration-specific. Direct reservoir-to-well transfer is the simplest and most programmatically friendly operation in liquid-handling—it can be expressed in a single line, using a handful of parameters. Better still, direct transfer can form the foundation of a framework seamlessly bridging plate design (and associated analytics) and programmatic implementation.

It is also extremely flexible. It is capable of generating any plate pattern. Our study implemented a 2-block plate pattern, with each block containing 20 different concentrations—a pattern already too complicated for a manual operator to implement reliably even at a modest scale. Randomization was not pursued in our study, since our results did not indicate the need to protect against local factors and we wished to keep results easier to visualize when mapped back onto the plates. The approach nonetheless should be implemented when possible in production. Our study was also restricted to the transfer of safe volumes (multiples of 10 μL) from one reservoir of solvent and one reservoir of solute. This was enough to sample the space of possible dilutions with sufficient precision. The direct transfer method is only limited by the minimal, safe volumes that can be transferred. Very fine rates of sampling can be achieved by adding reservoirs of different concentrations and dispatching from these reservoirs combinations of safe volumes to achieve the target dilutions—the same way one would pay with coins of different denominations. The approach can be further extended to more than one solute and/or solvent.

## Conclusion

In the paper, we have presented a study, conducted at the London Biofoundry, SynbiCITE, that approaches liquid-handling and its reliable automation from the unusual standpoint of vulnerability analysis. The study was centered around a very practical application (the construction of a calibration curve) and involved a mix of lycopene (a carotenoid of industrial interest) and the popular extractant DMSO. The study provided further evidence for the most desirable aspects of mechanized automation. Once the protocols are settled, they can be reliably repeated over dozens of plates, thus achieving a scale that is very difficult to reach with human operators. Automation also removes to a large extent the influence of the human operators. But the study also found evidence that automation is not a panacea and that automation does not, necessarily, produce better data and introduces new classes of errors to be controlled.

The control, quantification, and monitoring of automation-related errors will become central issues for the successful deployment of automation in synthetic biology. It is in this context that practical recommendations regarding automation and quality monitoring of liquid-handling have been shared in earlier sections of the paper.

## Methods

### Preparation of the Lycopene Standard

10 mg of standard pharma-grade lycopene powder (Sigma-Aldrich Lycopene ≥98% (HPLC) from Tomato SMB00706, REF: PHR1770-5X100MG 5 X 100 MG) was weighted on a precision scale (REF: OHAUS Pioneer PA214 Analytical Balance) in 20 mL pure DMSO (Fisher Scientific UK Ltd., DMSO, dimethyl sulfoxide, GC Headspace grade, Fisher Chemical, quantity: 1 L, packaging: glass bottle, CAS: 67-68-5, colorless, grade: GC Headspace, molecular formula: C_2_H_6_OS, molecular weight: 78.129, percent purity: >−99.9%, REF: 15572393) in a 50 mL Falcon tube, wrapped in aluminum foil to avoid exposure to light and vortexed (REF: Vortex IR StarLab Smart Instruments) for 5 min. This gives us an initial lycopene standard concentration C_0_^.^

### Automated Dilution Liquid-Handler Setup

Automation was carried out on an automated liquid handler (Analytik Jena CyBio Felix) with an 8-channel pipet head (CyBi-FeliX Head 8/250 μL, CyBi-FeliX SELECT Head 8/250 μL, REF: OL318-14-100).

DMSO was poured into a reservoir (Thermo Scientific Nalgene Robotic Reservoirs Flat Bottom, Sterile, Size 300 mL, REF: 1200–1301) and placed on deck position 11. A volume of 10 mL of lycopene standard at initial concentration C_0_ was manually pipetted into a 12-channel reservoir plate (4titude 12 channel reservoir plate, 21 mL square channels, pyramid bottom, REF: 4ti-0131) and placed on deck position 8. Two boxes of 250 μL tips (AJ CyBio RoboTipTray 96/250 μL, REF: OL3810-25-669) were placed on deck positions 1 and 2. For the serial dilution protocol, a mixing deep-well plate (Greiner Masterblock 96 well, 2 mL, PP, V-Bottom, Natural, REF: 780285) was placed on deck position 9. A measurement plate (Greiner Flat-bottom plate REF: 655161) was placed on deck position 12.

All versions of the automated protocols referred in this paper were named according to a common practice in software engineering known as semantic versioning: V X.Y.Z, where the first digit (X) corresponds to the general approach to dilution: 1 was used for the sequential dilution approach; 2 for the linear dilution approach. As is commonly used in software-engineering, the second digit (Y) was incremented when a major revision was introduced, while the final digit (Z) was incremented for every minor revision.

Further details on the two main protocols can be found in the Supporting Information (Section 7). This includes the following pieces of information:

- Protocol history: in addition to the nomenclature, the changes from one version to the next and their purpose are listed.
- Values of the protocol variables: the values of the most important parameters (aspiration, dispense, Z-drive speed) are listed for all the protocol versions.
- Step-by-step representation of the protocols and deck layout.

For the sake of completeness, a zip archive (named LycopeneCalibration_Protocols.zip) containing the execution logs for the various protocol versions (parameter assignments, and sequence of operations) has also been shared.

### Food Colorant

Sunset Red (E110), Royal Blue (E110, E123, E133), Fruit Green (E102, E133) (ingredients: water, sodium carboxymethylcellulose, sorbitol, propylene, glycol, glycerin) (REF: Ktdorns Food coloring).

### Spectral Measurements

UV/vis absorbance (full spectrum: wavelength: 350–650 nm, wavelength step width: 2 nm) measurements were done in two different microplate readers of the same model (REF: BMG LABTECH CLARIOstar Plus).

### Data Analysis

All the data analysis was conducted in Python (3.11) on the Anaconda platform using the following scientific libraries: SciPy, NumPy, Pandas, Scikit-Learn, MatplotLib, and Seaborn.

## Supporting Information Available

Section 1: implemented dilution schemes (Figures S1 and S2; Tables S1 and S2); Section 2: geometric dilution scheme—food colorant + water (Figures S3–S22); Section 3: geometric dilution scheme—lycopene in DMSO (Figures S23–S32; Tables S3–S5); Section 4: linear dilution scheme—preliminary studies (Figures S33–S39); Section 5: main analytical methods (Figures S40–S44; Table S6); Section 6: linear dilution scheme—lycopene in DMSO (Figures S45–S51, Table S7); Section 7: automated protocols (Figures S52–S57, Table S8–S11) (PDF)
Experimental details on all the runs, mixes, and protocols (Obsidian Vault – Markup files) (ZIP)
All the measurement data for the runs (ZIP)
Execution logs for the various protocol versions (txt files) (ZIP)
