# Supplementary material for: Construction of a Calibration Curve for Lycopene on a Liquid-Handling Platform—Wider Lessons for the Development of Automated Dilution Protocols
Source: ACS Synth Biol. 2024 Aug 3;13(8):2357–75. doi: 10.1021/acssynbio.4c00031 (PMC11334188; doi:10.1021/acssynbio.4c00031)
Supplement: Supplementary file 1 — sb4c00031_si_001.pdf [file sb4c00031_si_001.pdf]

Supporting Information

Construction of a Calibration Curve for Lycopene on a Liquid-Handling Platform - Wider Lessons for the Development of Automated Dilution Protocols

Authors:

Matthieu Bultelle, Alexis Casas, and Richard Kitney ∗

Department of Bioengineering, Imperial College London

Exhibition Road, London, SW7 2BX, United Kingdom

Corresponding Author: Richard Kitney

E-mail: r.kitney@imperial.ac.uk

1 - Implemented Dilution Schemes

1.1 - Geometric Dilution Scheme

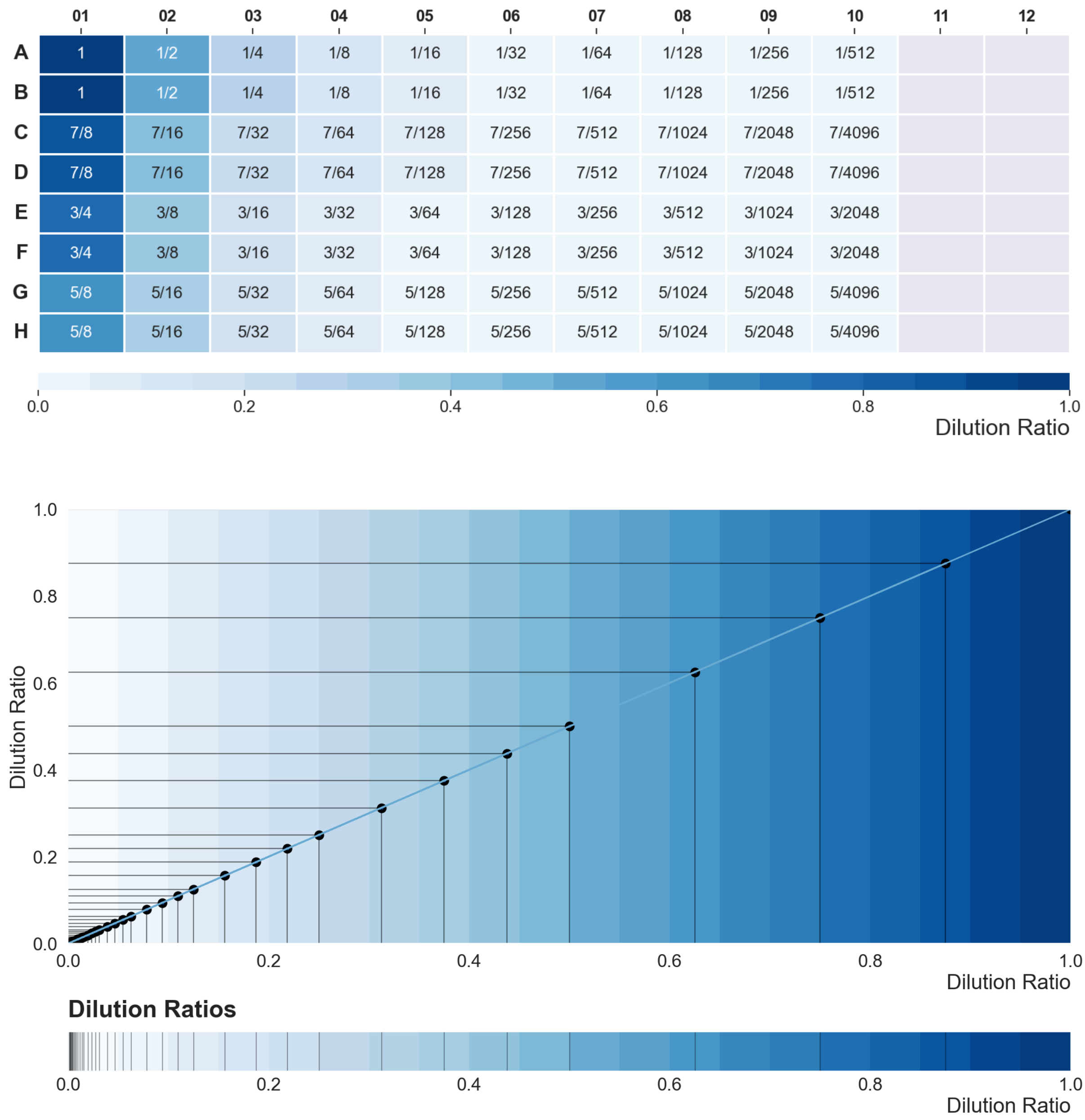

Figure S1 - Plate Map for the Geometric Scheme and Corresponding Dilution Density

1.2 - Linear Dilution Scheme

	01	02	03	04	05	06	07	08	09	10	11	12
A	100%	80%	60%	40%	20%	100%	80%	60%	40%	20%		
B	100%	80%	60%	40%	20%	100%	80%	60%	40%	20%		
C	95%	75%	55%	35%	15%	95%	75%	55%	35%	15%		
D	95%	75%	55%	35%	15%	95%	75%	55%	35%	15%		
E	90%	70%	50%	30%	10%	90%	70%	50%	30%	10%		
F	90%	70%	50%	30%	10%	90%	70%	50%	30%	10%		
G	85%	65%	45%	25%	05%	85%	65%	45%	25%	05%		
H	85%	65%	45%	25%	05%	85%	65%	45%	25%	05%		

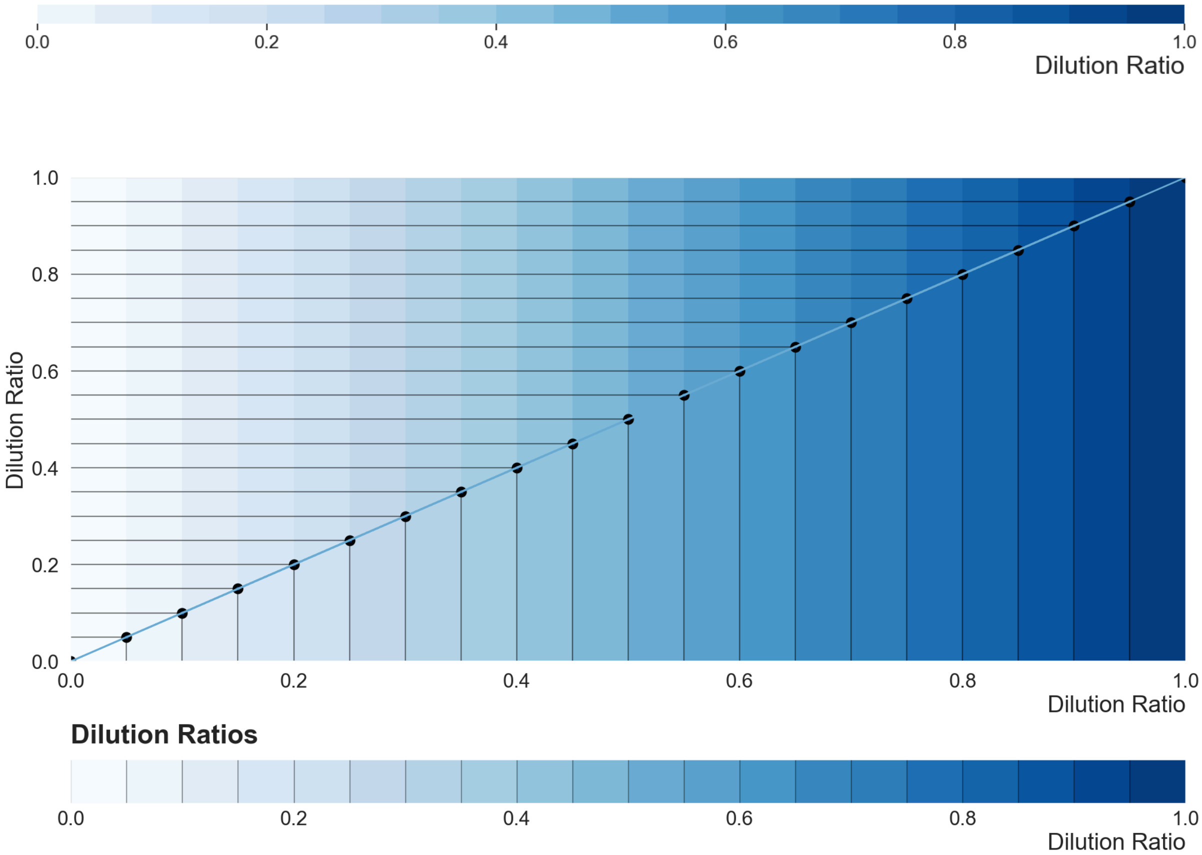

Figure S2 - Plate Map for the Linear Scheme and Corresponding Dilution Density

1.3 - Comparison between Schemes

Geometric Dilution Scheme	
**Transfer Method**	**Iterative Transfer Workflow.** Transfers liquid from column to column. Direct adaptation of the serial dilution protocol.
**Flexibility**	**Low.** Iterative transfer only allows for coarse sampling of dilution space (E.G halving)
**Complexity**	**Very Simple.** Mostly relying on column-wise operations. Muscle memory can be developed for manual implementations. Little possibility of errors even when repeated many times manually
**Transfers**	**Large Volumes.** Well within specifications of platforms
**Pros**	**Simplicity // Very Friendly to Manual Implementation**
**Cons**	**Lack of Flexibility**
**Linear Dilution Scheme**	
**Transfer Method**	**Direct Transfer Workflow.** Transfers from reservoirs to wells.
**Flexibility**	**Low.** Iterative transfer only allows for coarse sampling of dilution space (E.G halving)
**Complexity**	**Middle to High.** The content of all the wells in the linear dilution scheme is generated with simple operations, but the number of combinations is high (21). The spatial distribution, although not random, requires a lot of care if implemented manually
**Transfers**	**Any Volume in Theory.** Direct transfer grants greater freedom in terms of achievable concentration distribution. Our implementation generates a linear range of dilutions between 0 and 1, with an increment of 5% (corresponding to 10µl out of a total volume of 200µl - a quantity well within the capacity of the CyBio Felix automated platform to deliver reliably).
**Pros**	**Flexibility.** A linear spread is better suited to the construction of a standard curve: it has fewer points clustered close to the origin and will be less sensitive to samples for the larger concentrations. It will also offer a better localization of any inflection point in the curve if saturation effects are present.
**Cons**	**Relative Complexity - Automation Only**

Table S1 : Comparisons of the Two Dilution Schemes

Source of Error	Geometric Scheme	Linear Scheme
**Volumes Transferred** (Depends on Platform)	**Only Large (Safe) Volumes**	**Any Volume in Theory.** Increments chosen so only safe volumes transferred
**Contamination by Falling Droplets** (Depends on Choice of Tips & Dispensed Liquid)	**Minimal.** Only the first column is constructed by direct transfer. Column by column transfers are short.<br><br>DMSO was dispatched first to minimize risks	**Possible.** The linear dilution scheme involves a lot of travels between wells and reservoirs - each with its own probability of causing contamination with falling droplets. Same safety measure for DMSO
**Reservoir Lycopene Concentration**	**Potentially Severe.** Same as for the linear scheme will only affect column 1.	**Potentially Severe.** At high concentration, the mix is expected to be difficult to dispense - affecting all wells in columns 1-10
**Liquid Homogeneity** (Between Wells)	**Potentially Severe.** With the iterative transfers, most wells are used as sources. Pipetting precision may be affected by a one-size-fits-all approach for the aspiration and dispensation parameters.	**Irrelevant.** Only two homogenous reservoirs are dispensed from. Aspiration and dispensation parameters can be set up globally.
**Type of Error**	**Geometric Scheme**	**Linear Scheme**
**Independent Errors**	**Yes.**<br>- Contamination<br>- Direct Transfers in First Column	**Yes.**<br>- Contamination<br>- All Direct Transfers
**Correlated Errors**	**Yes.** Transfer column by column propagates errors	**None Expected**

Table S2 - Possible Source of Errors (after Albert's Classification).

2 - Geometric Dilution Scheme - Food Colorant + Water

In this section we present the main results for the preliminary study of the Geometric Dilution Scheme. These results correspond to the training runs 55 and 57. Each run comprises a plate generated with an automated version of the protocol, and one generated manually. Both runs are for a development mix of food colorant in water.

2.1- Run 55 - Plate 01 - Automated

Mix: Food colorant in Water - Used same preparation mix (Tube 01) as Run 055 - Plate 02
Protocol: Automated Protocol V1.0.0

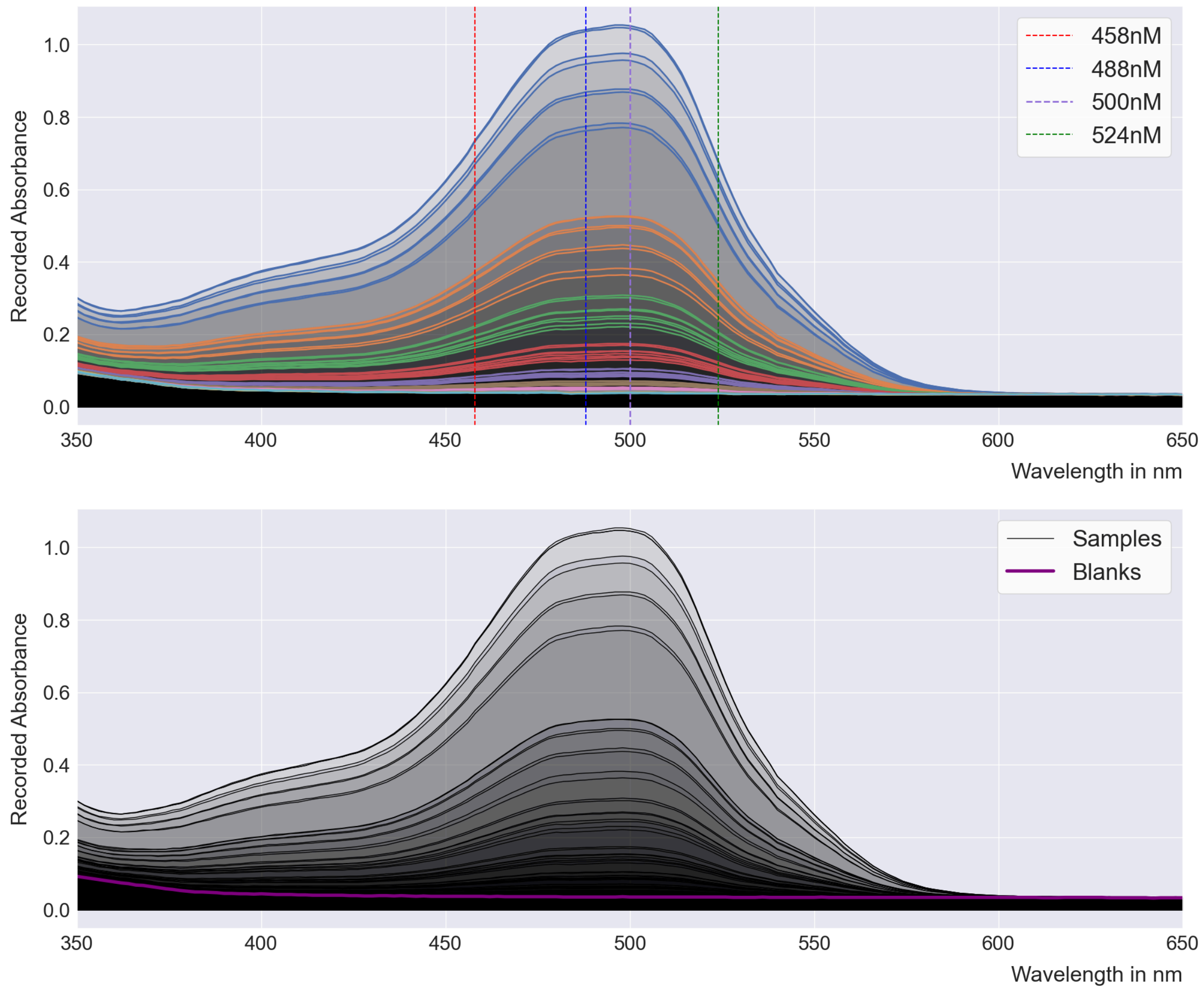

Figure S3 - Run 55 - Plate 01 - Absorbance Spectrum for Samples and Blanks

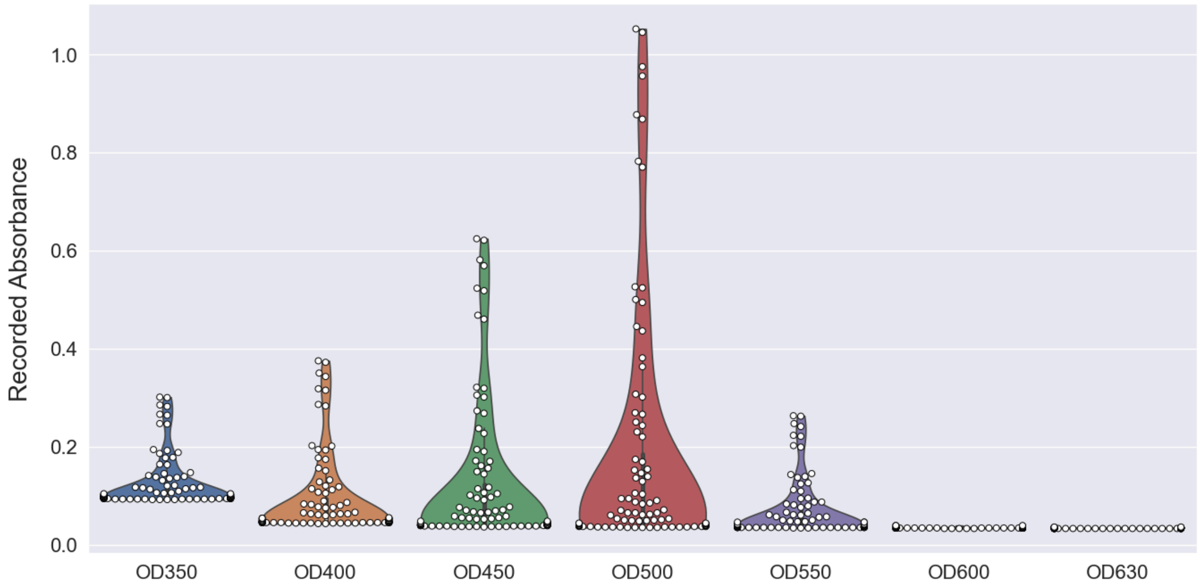

Figure S4 - Run 55 - Plate 01 - Corresponding Violin Plot at Selected Wavelengths

The violin plots display similar shapes, albeit stretched out, for wavelengths from 350 to 550 nm. All distributions for these wavelengths are very asymmetrical due to the lopsided dilution scheme (in particular an accumulation at small concentrations). No information is to be found above 600 nm (flat spectrum).

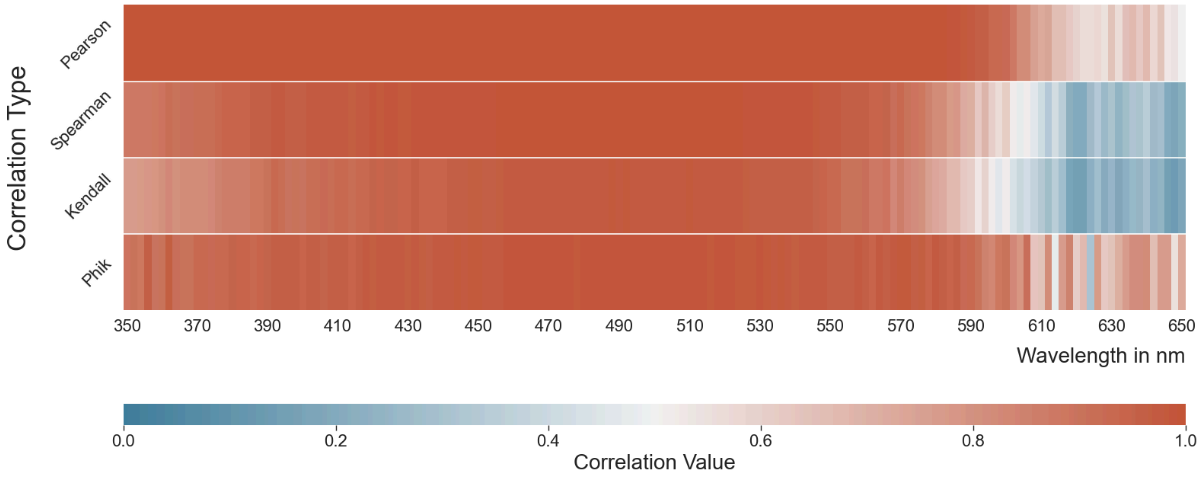

Figure S5 - Run 55 - Plate 01 - Correlations Between Dilution Ratio and Measurements

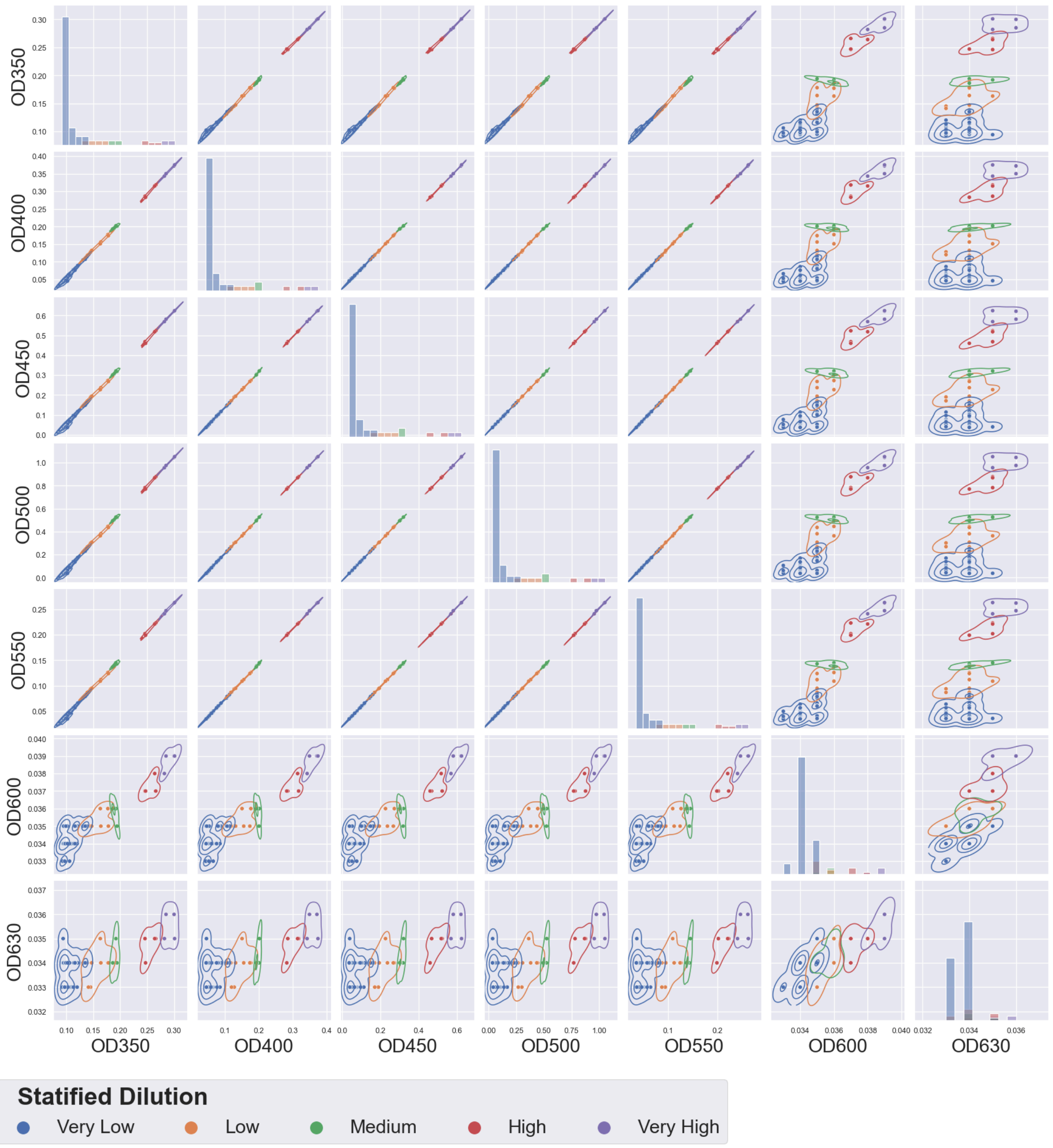

Figure S6 - Run 55 - Plate 01 - Pairplot Between Measurements at Selected Wavelengths
Very high correlation between measurements for wavelengths between 400 and 550 nm.

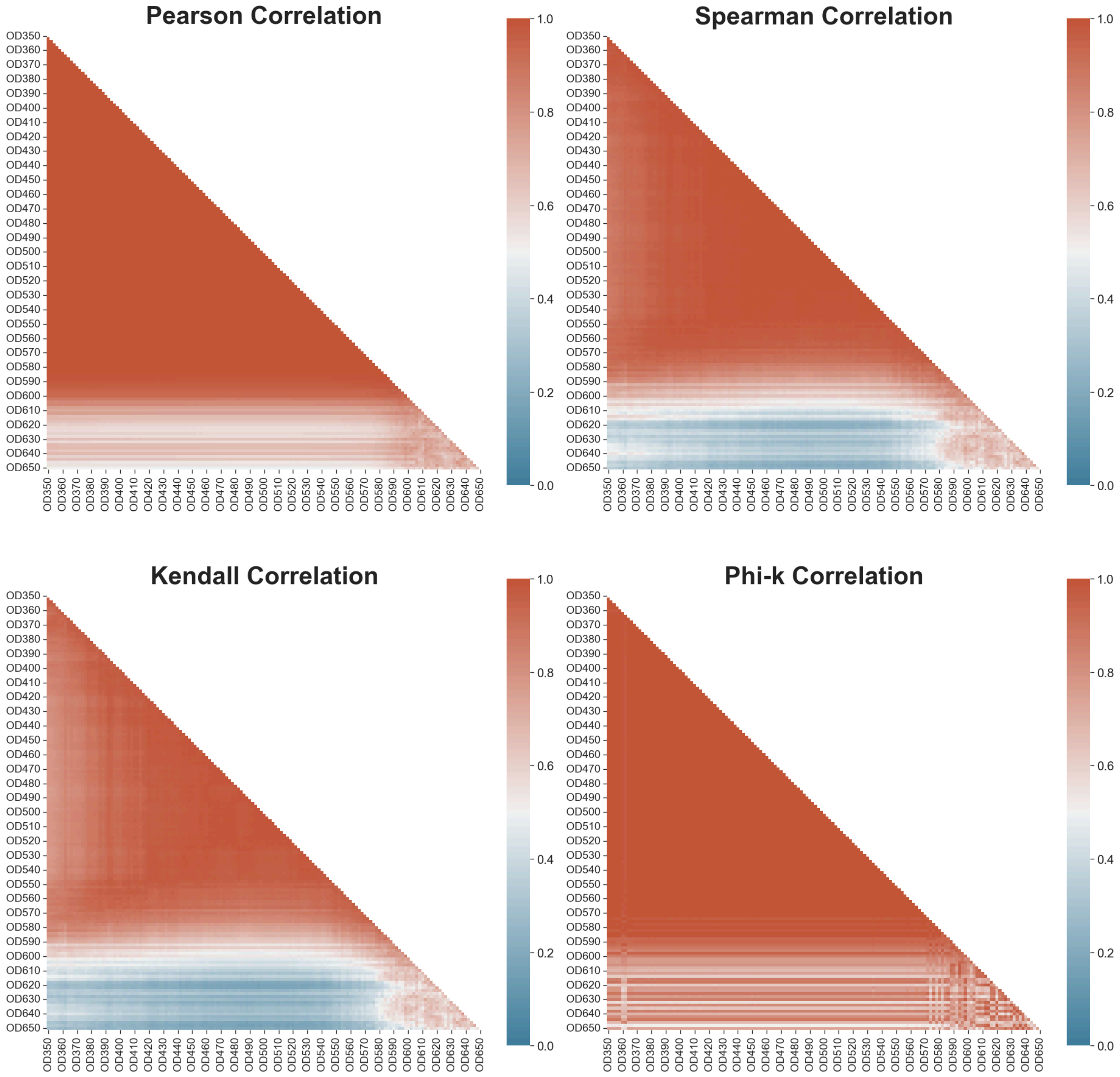

Figure S7 - Run 55 - Plate 01 - Correlation Matrices Between Measurements at Different Wavelengths

2.2 - Run 55 - Plate 02 - Manual

Mix: Food colorant in Water - Used same preparation mix (Tube 01) as Run 055 - Plate 01
Protocol: Manual Protocol

Findings:

- Tighter repeats (noticeable at larger concentrations)
- Sharper correlations matrices
- Lack of correlation at wavelengths larger than 600 nm is more pronounced.

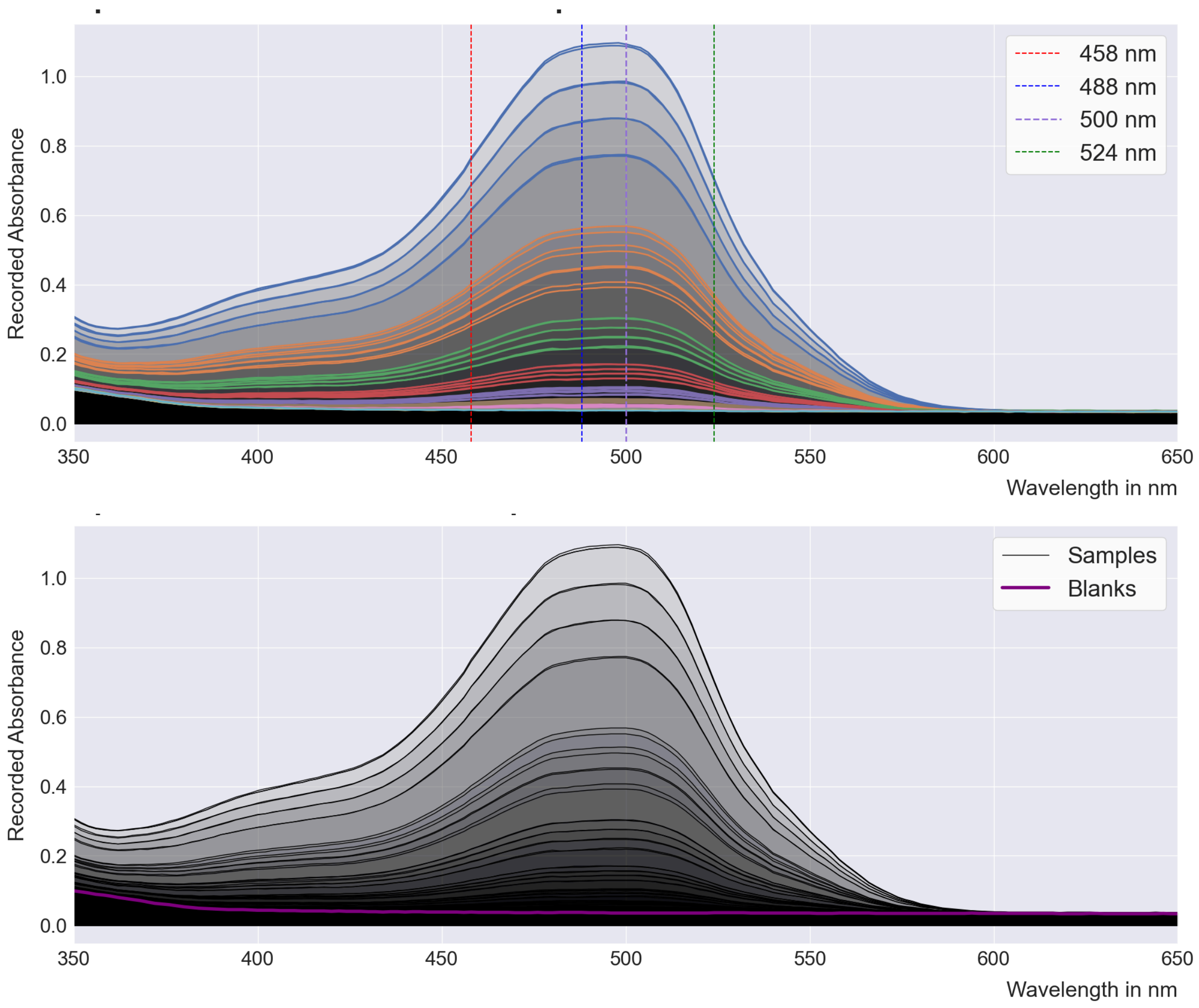

Figure S8 - Run 55 - Plate 02 - Absorbance Spectrum for Samples and Blanks

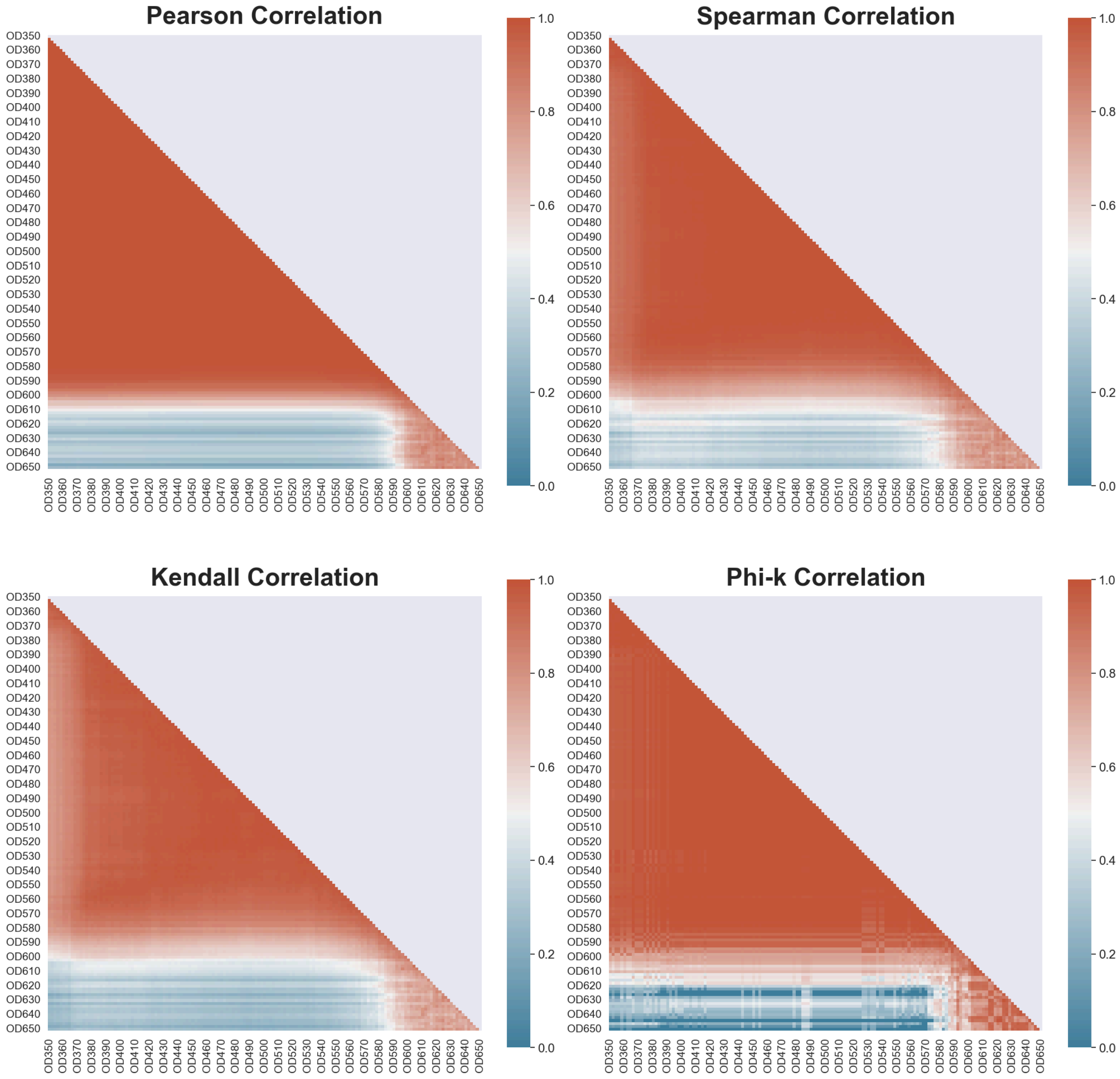

Figure S9 - Run 55 - Plate 02 - Correlation Matrices Between Measurements

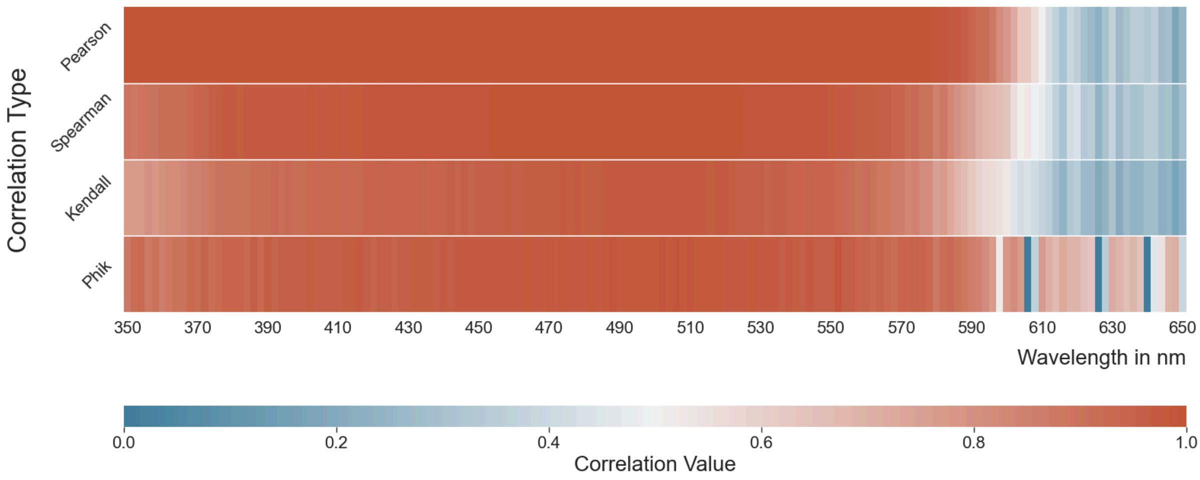

Figure S10 - Run 55 - Plate 02 - Correlations Between Dilution Ratio and Measurements

2.3 - Run 57 - Plate 01

Mix: Food colorant in Water - Used same preparation mix (Tube 01) as Run 057 - Plate 02 - but different mix than Run 055

Protocol: Automated Protocol V1.0.0

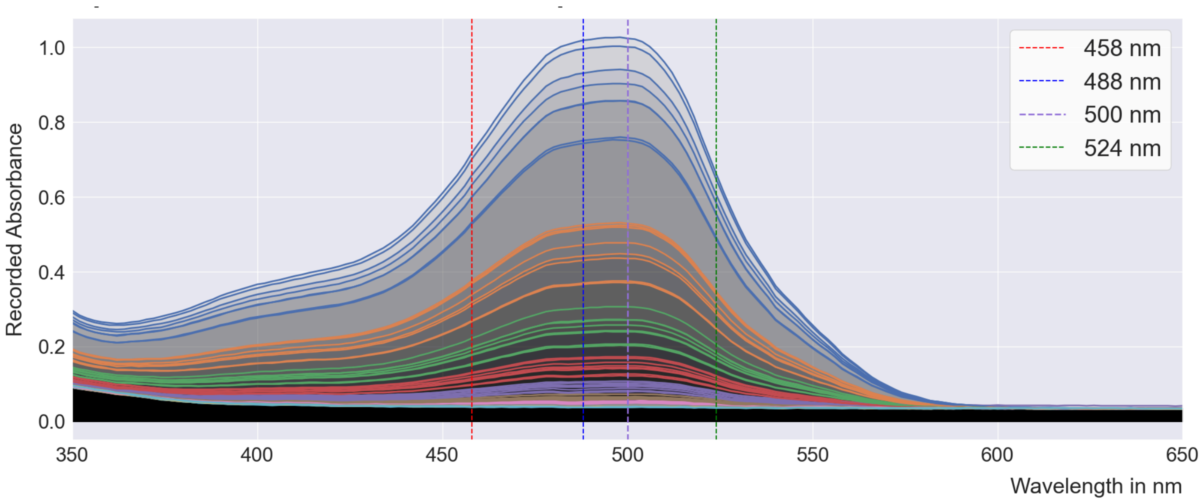

Figure S11 - Run 57 - Plate 01 - Absorbance Spectrum for All Samples

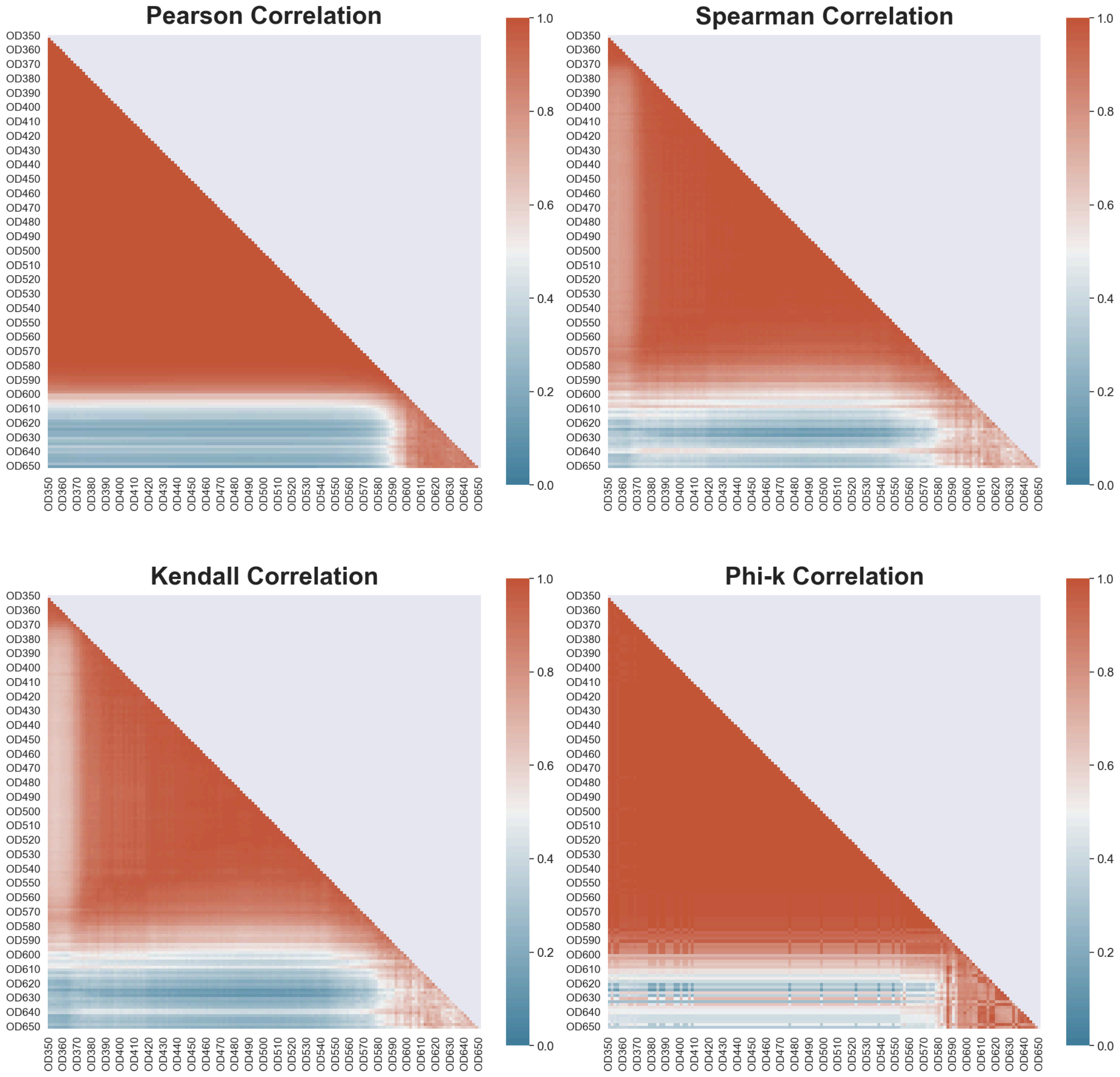

Figure S12 - Run 57 - Plate 01 - Correlation Matrices Between Measurements

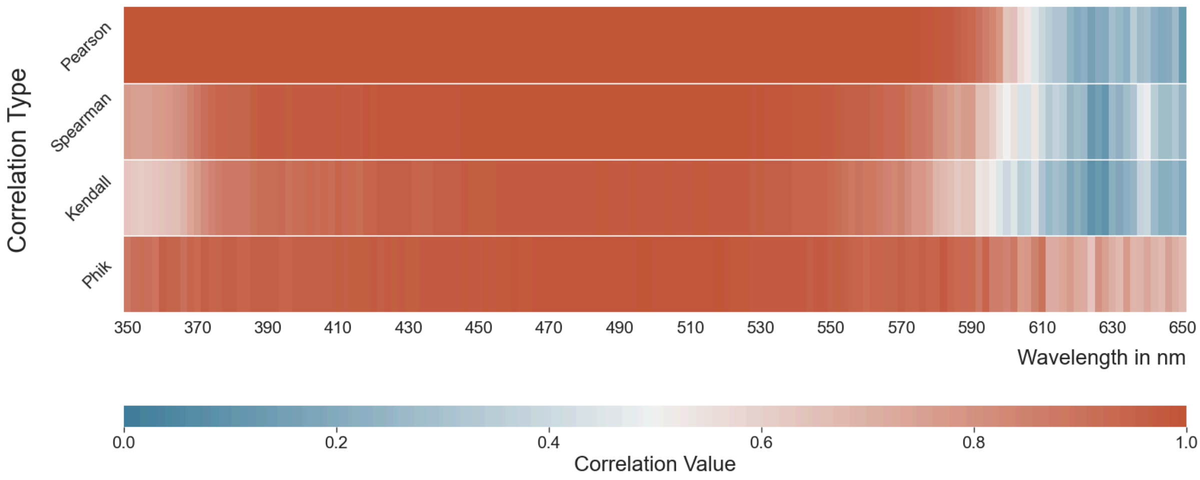

Figure S13 - Run 57 - Plate 01 - Correlations Between Dilution Ratio and Measurements

2.4 - Run 57 - Plate 02

Mix: Food colorant in Water - Used same preparation mix (Tube 01) as Run 057 - Plate 01 - but different mix than Run 055
Protocol: Manual

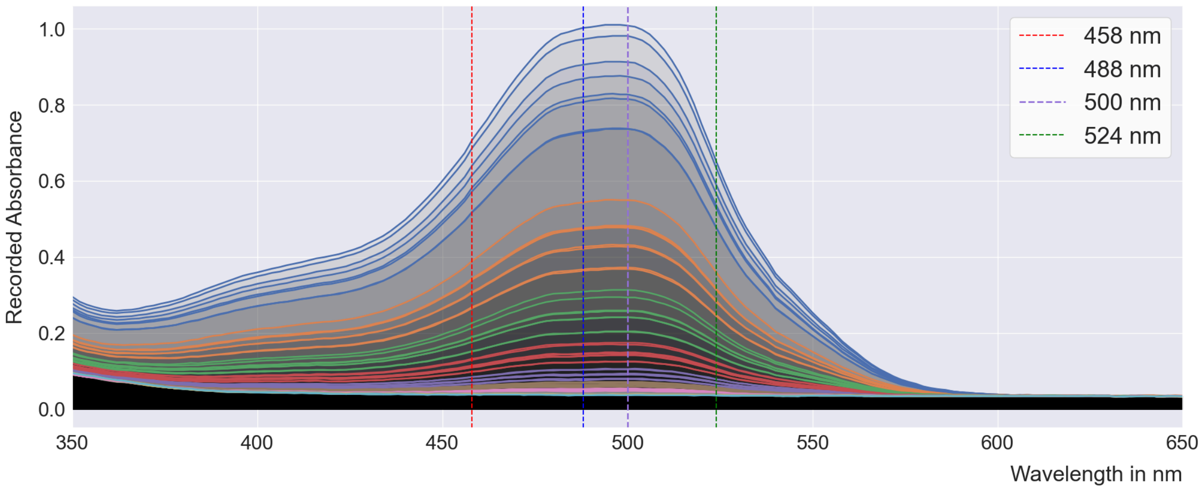

Figure S14 - Run 57 - Plate 02 - Absorbance Spectrum for All Samples

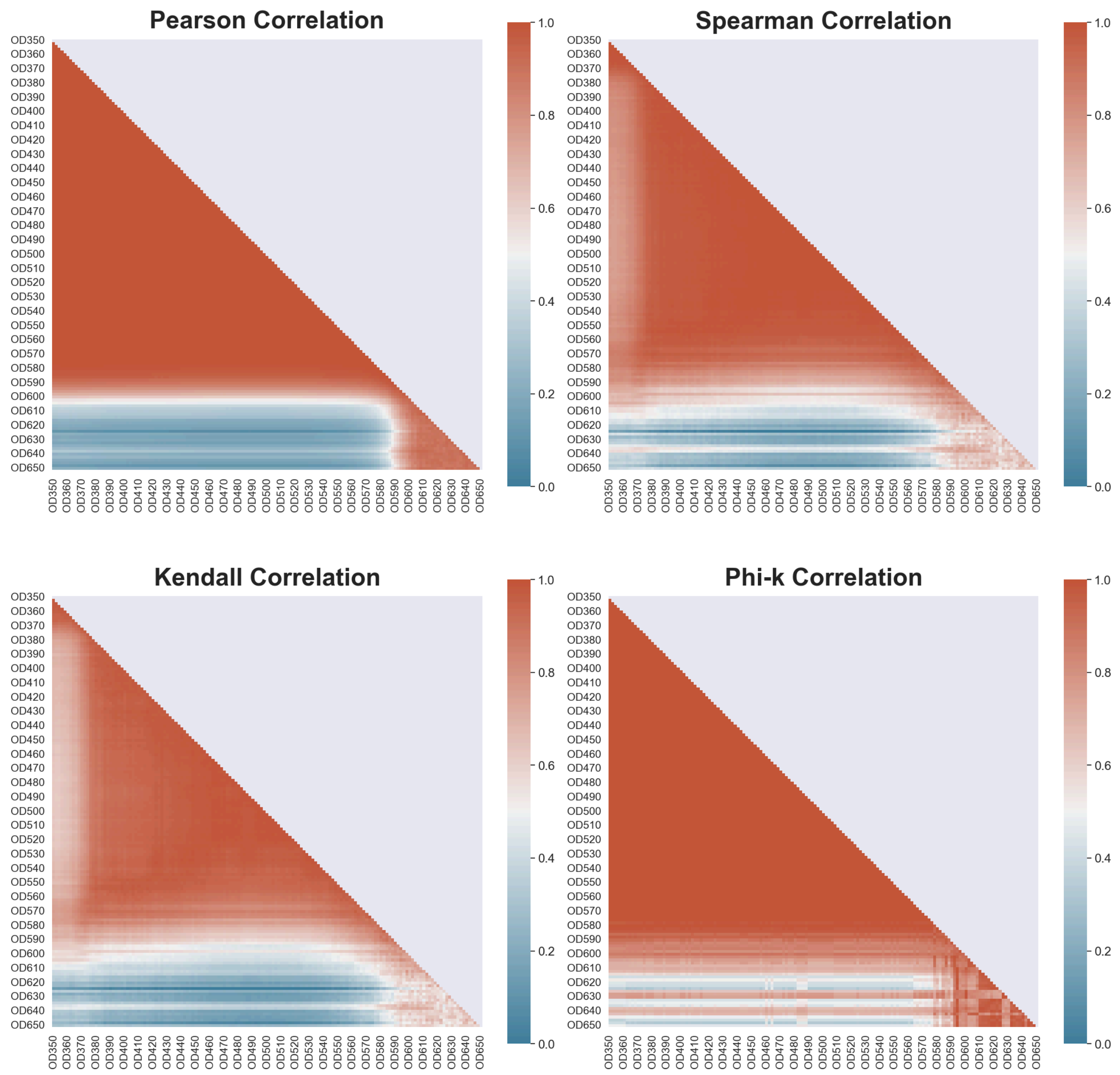

Figure S15 - Run 57 - Plate 02 - Correlation Matrices Between Measurements

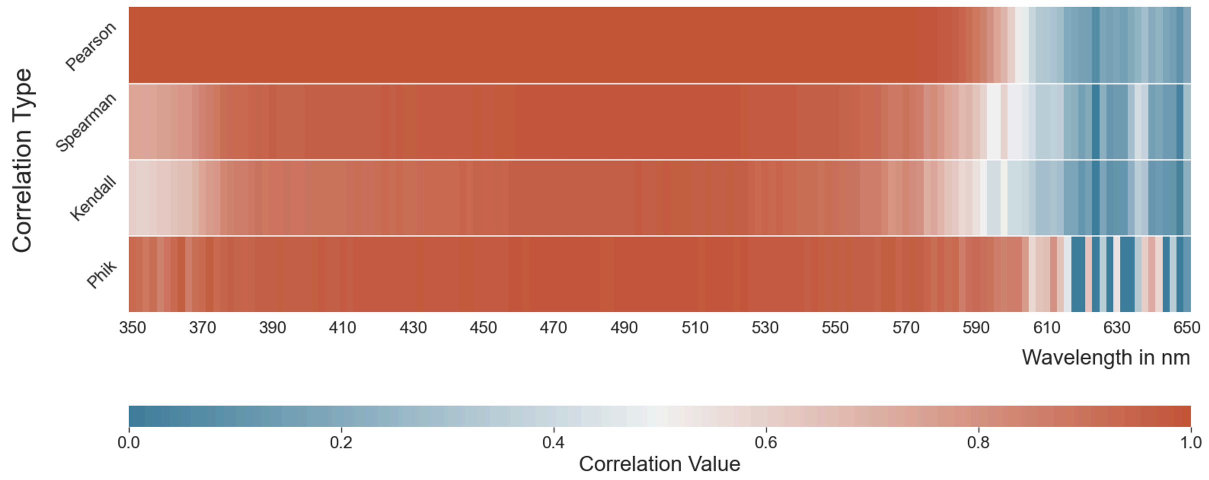

Figure S16 - Run 57 - Plate 02 - Correlations Between Dilution Ratio and Measurements

2.5 - Run 55 & 57 - Comparison Automated Vs Manual

Runs 55 and 57 were conducted with mixes of food colorant in water. In both runs a plate (Plate 01) was generated with the automated implementation, and another (Plate 02) was generated manually from the same mix freshly made on the day. Every time the assays were run by the same experimentalist (AC - who ran all the assays, manual and automated, in this study), with the same pipetting equipment.
In Run 55, the measurements for the manually-generated plate were always a bit higher. In Run 57, measurements for manually and automatically-generated plates matched each other. Analysis of the variations between repeats (not included here) on the same plate showed that the plates generated with automation had similar errors - while the manually-generated plates had either tighter repeats in the first run, and equally, as the automation-generated ones in the second run. We attributed the variations for manual plates to the experimentalist and day-to-day variations.

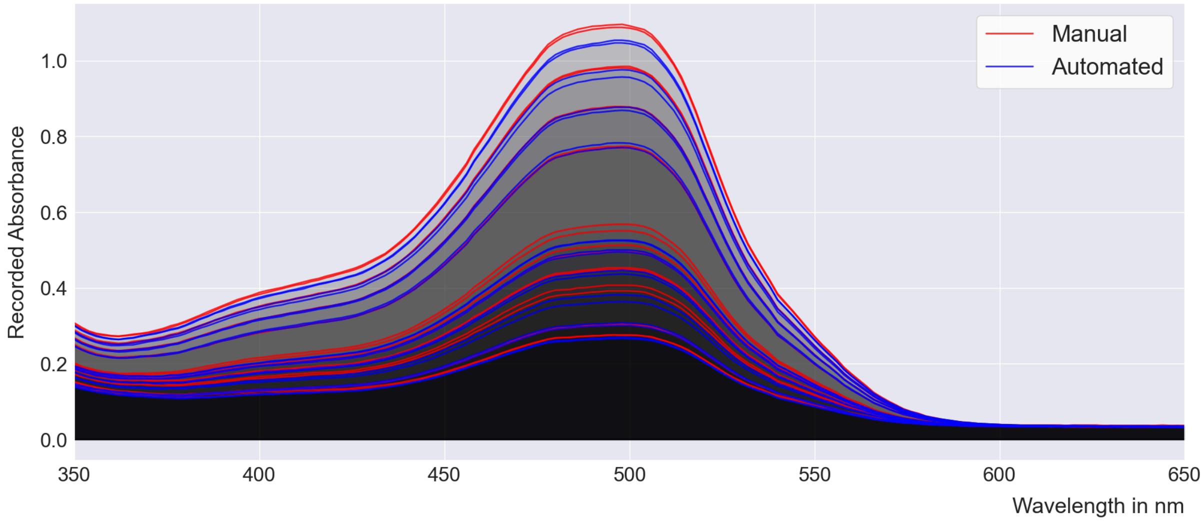

Figure S17 - Run 55 - Comparison between Automation-Generated Data (Plate 01, in Blue) and Manually Generated Data (Plate 02 in Red)

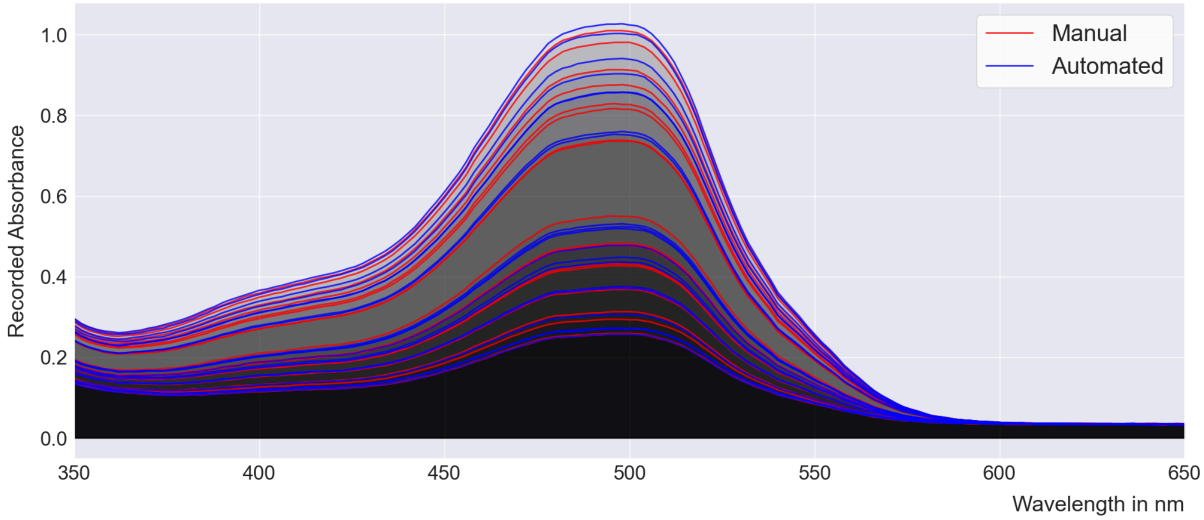

Figure S18 - Run 57 - Comparison between Automation-Generated Data (Plate 01, in Blue) and Manually Generated Data (Plate 02 in Red)

2.6 - Assessing the Effects of the Iterative Transfers

Since the protocol uses iterative transfers from one column to the next we stratified the data by column and checked for they exhibited similar patterns as the first column (since they inherited them by iterative transfer). In practice we could only observe the first 3 columns - the dilution ratios were too small afterwards). And due to the limited size of the data from a column, linear regression was used on the 8 sample values in a column
The following points are used to explain the data:

1. If the transfer between columns are perfectly implemented, then the trendline in the first also appears in the subsequent columns – that is the trendlines are parallel
2. Also, if the transfers in the first columns (dilution ratios of 100%,87.5%,75% and 62.5 %) are perfectly implemented, the trendlines are not only parallel, but also line up
3. Conversely, if the volume transferred from one column to the next is off by a factor α (instead of transferring a volume V as programmed, (1- α)V is transferred) then the trendlines do not stay parallel and instead their slope increases (the ratio of the column 2 and column 1 is 2+ α for instance).

Figures S19-S22 show results that are consistent with the third scenario: imperfect transfer from one column to the next. Automated protocol performs worse than the manual protocol – although even data for the manual protocol show jumps between columns, and trend lines that do not stay parallel.

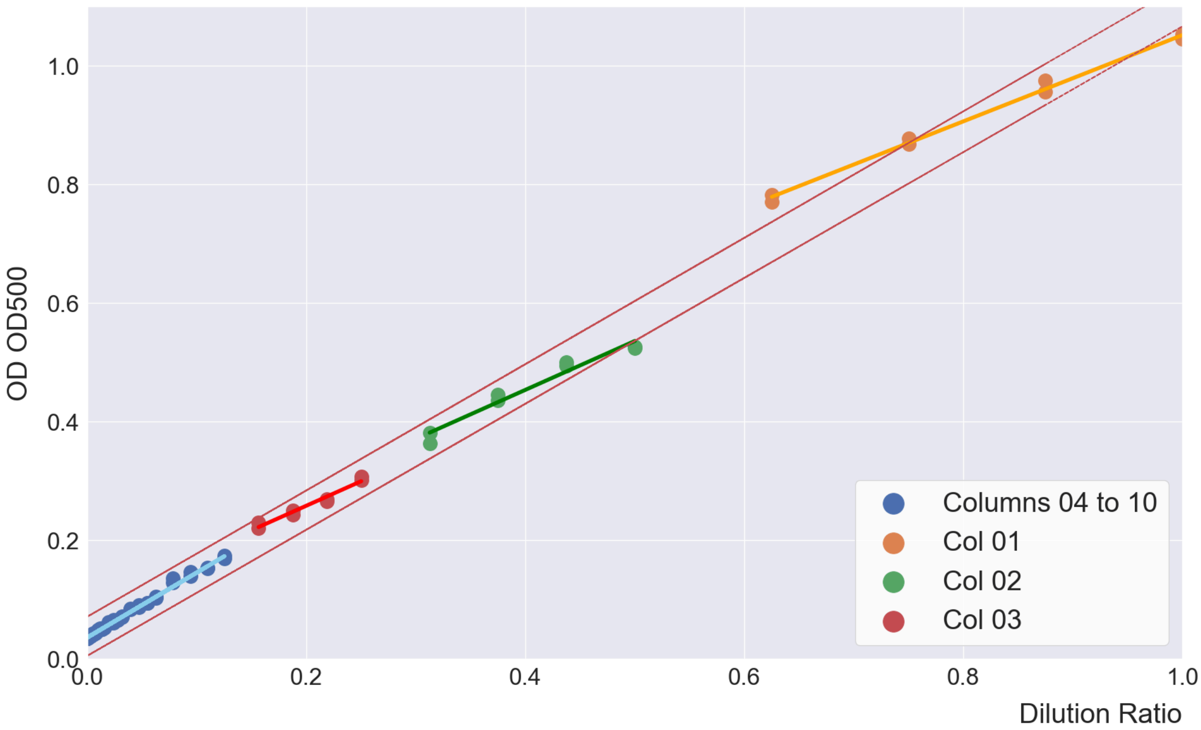

Figure S19 - Run 55 - Plate 01 (Automated) - Effects of the Iterative Dilutions at 500 nm

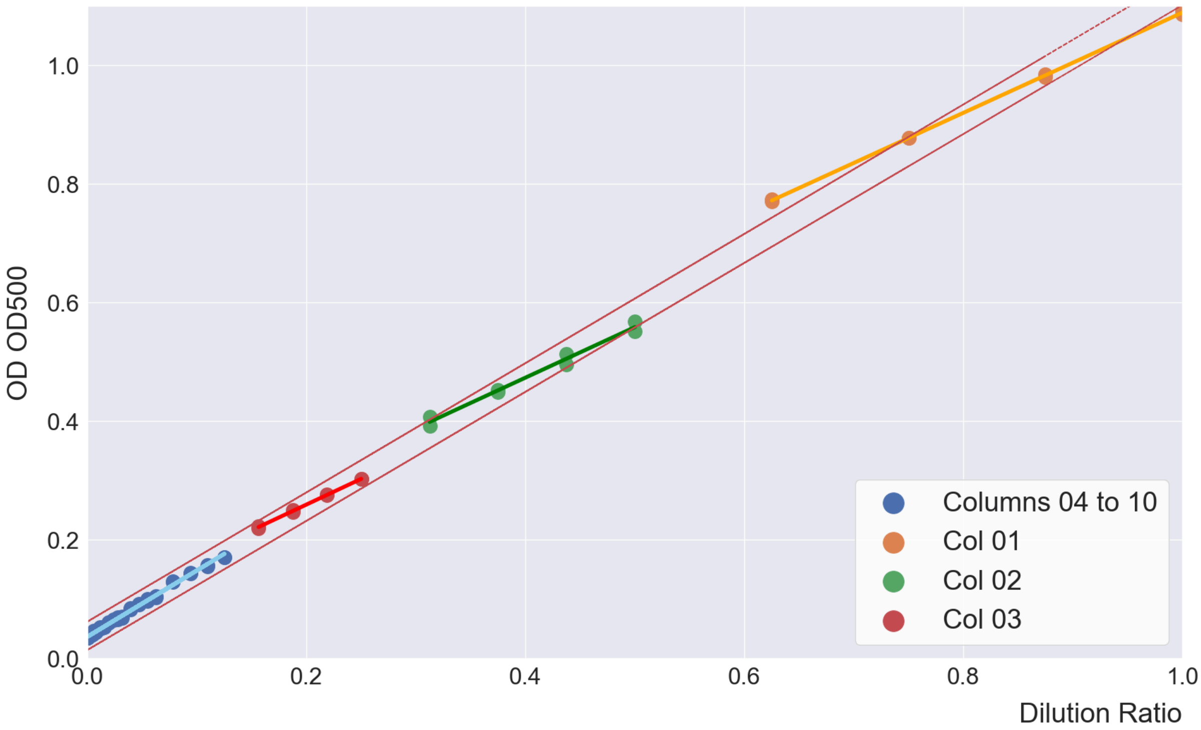

Figure S20 - Run 55 - Plate 02 (Manual) - Effects of the Iterative Dilutions at 500 nm

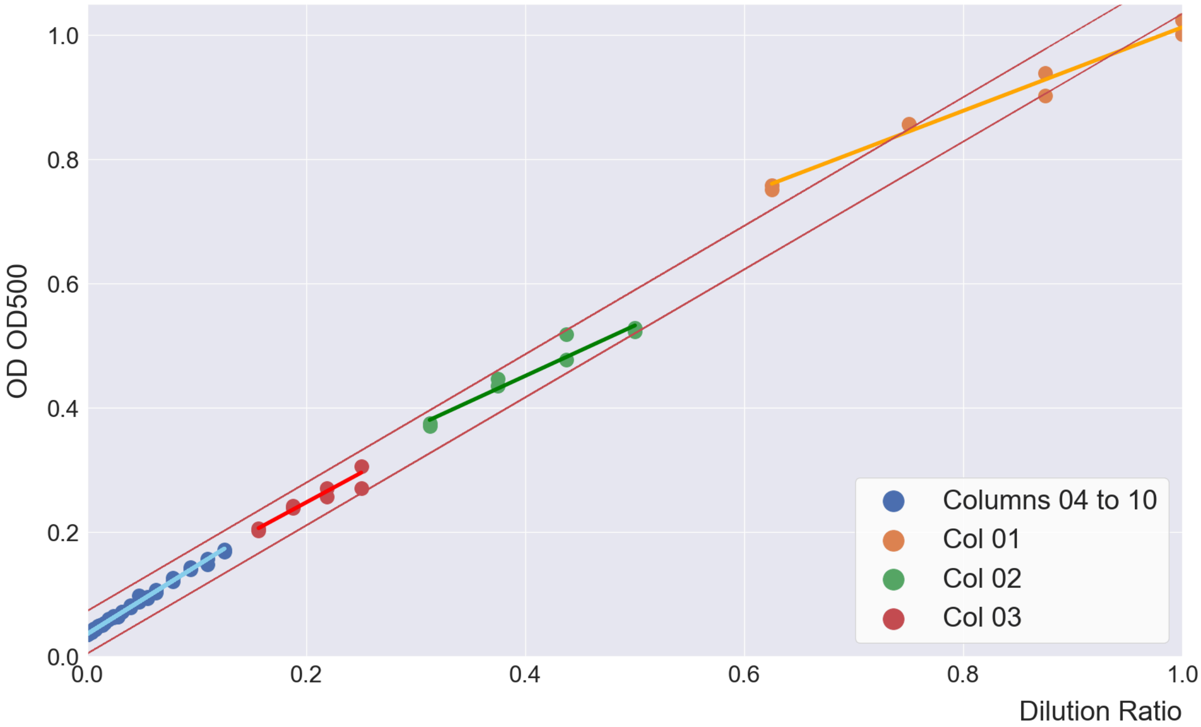

Figure S21 - Run 57 - Plate 01 (Automated) - Effects of the Iterative Dilutions at 500 nm

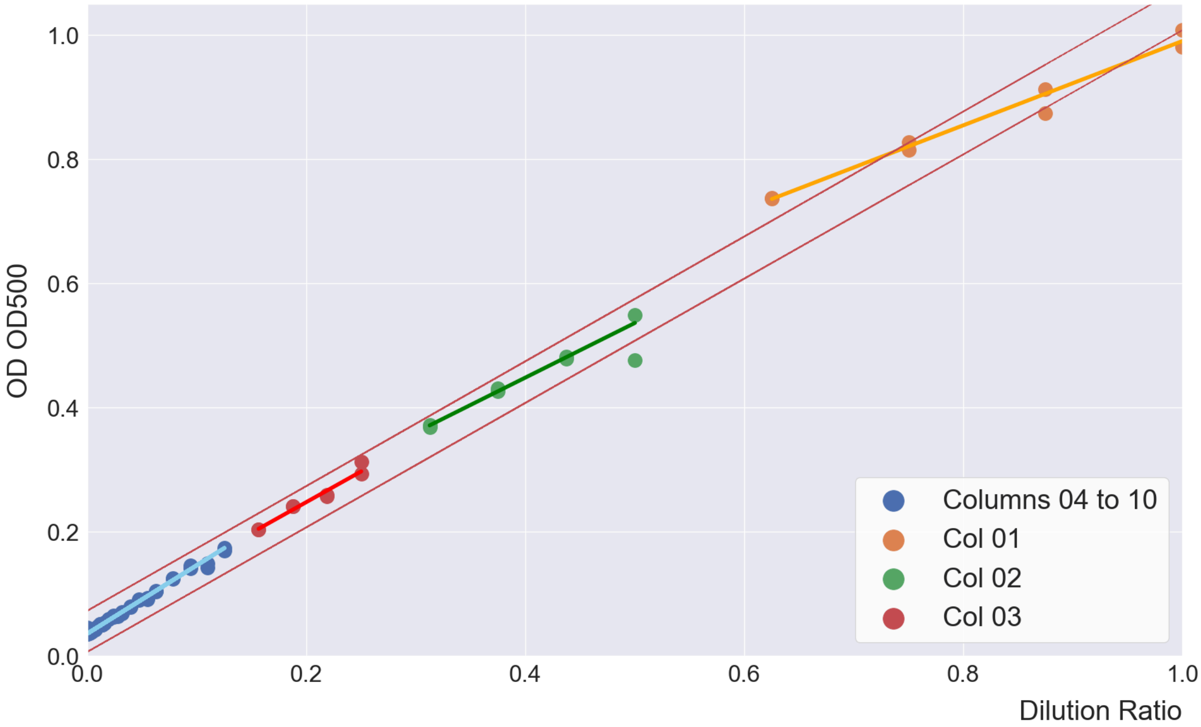

Figure S22 - Run 57 - Plate 02 (Manual) - Effects of the Iterative Dilutions at 500 nm

3 - Geometric Dilution Scheme - Lycopene in DMSO

3.1 - Run 62 - Plate 01 - Automated

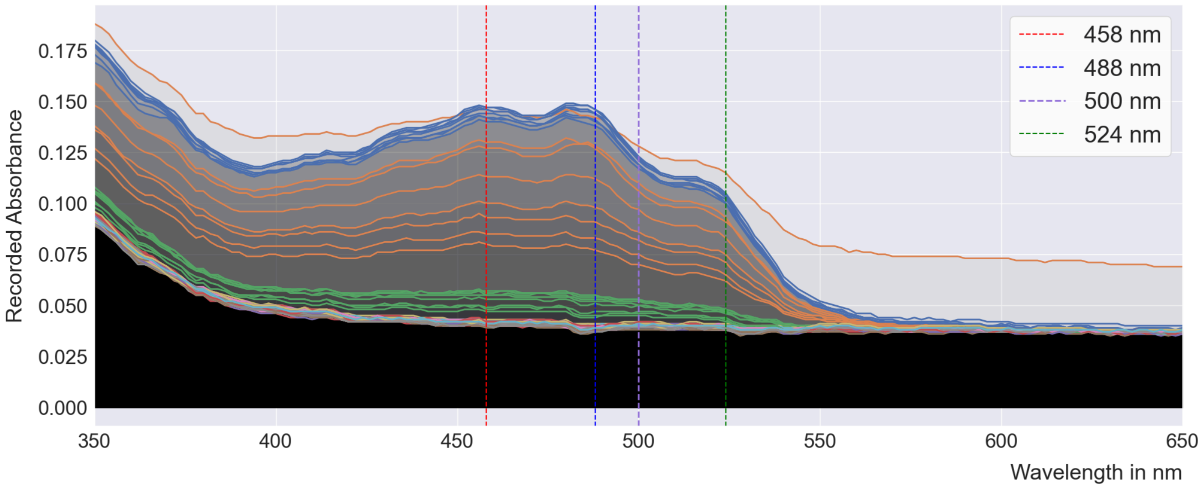

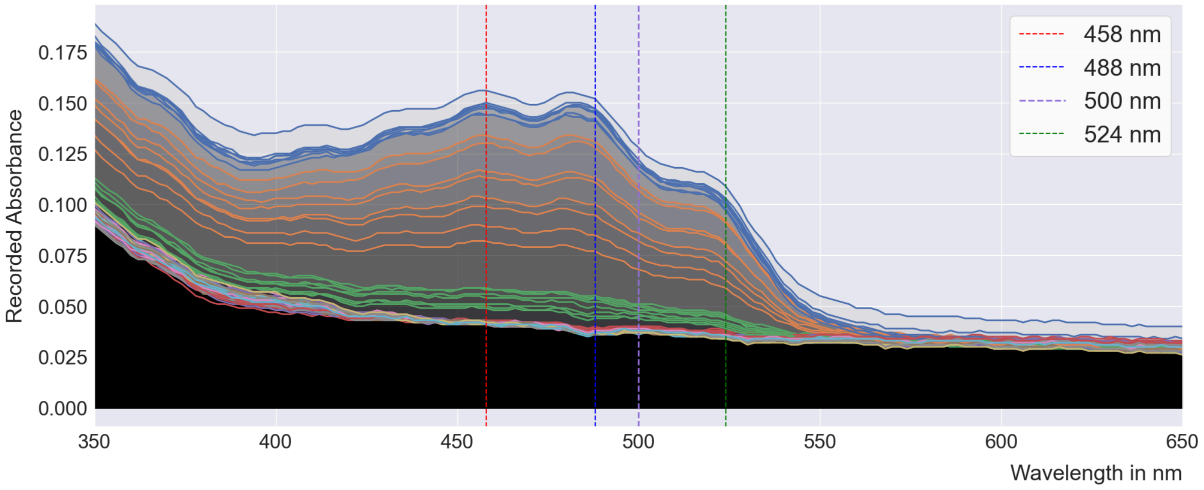

Figure S23 - Run 62 - Plate 01 - Absorbance Spectrum for All Samples.
Top: Measurements in Clariostar A - Bottom: Measurements in Clariostar B

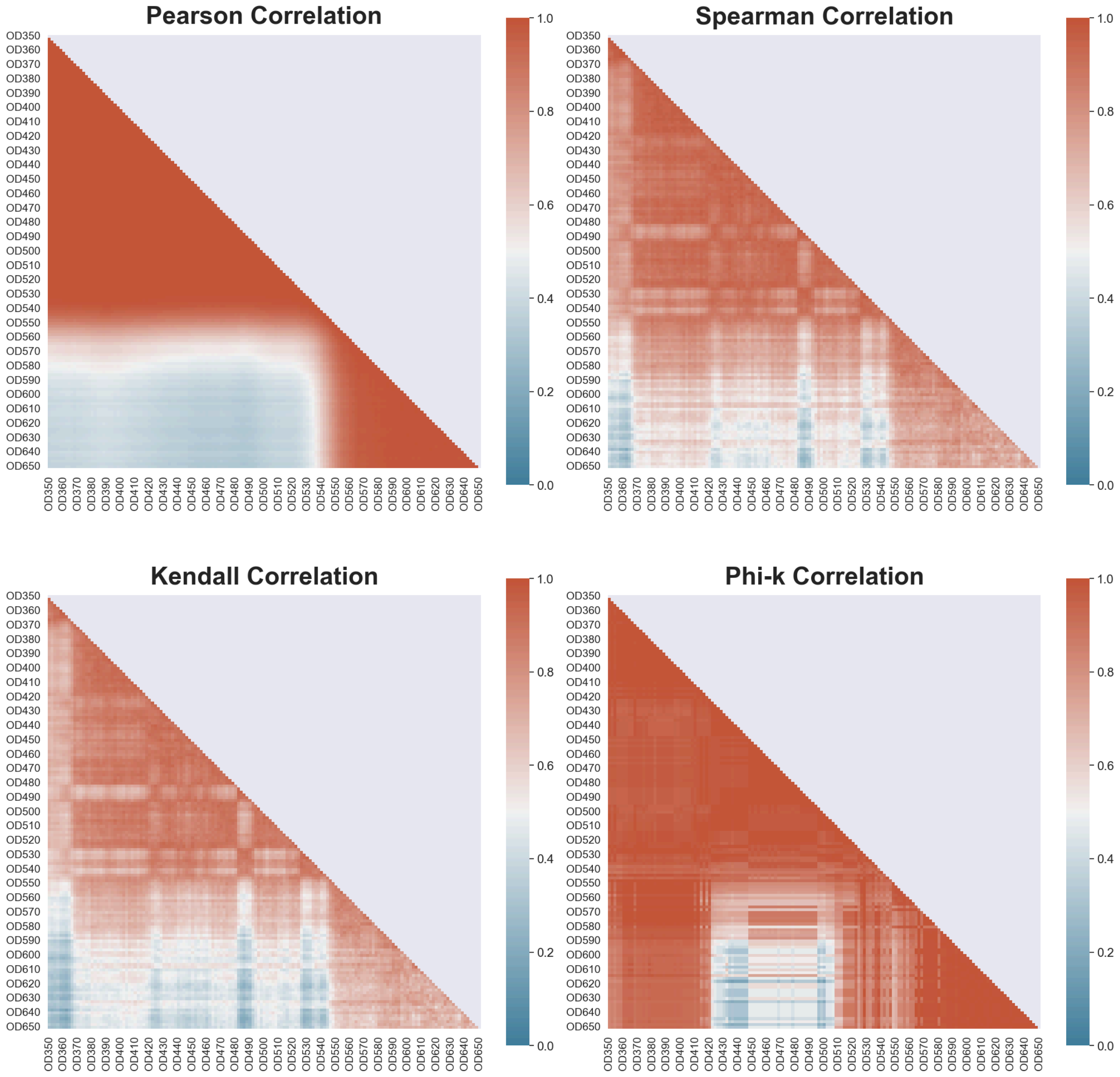

Figure S24 - Run 62 - Plate 01 - Correlation Matrices Between Measurements

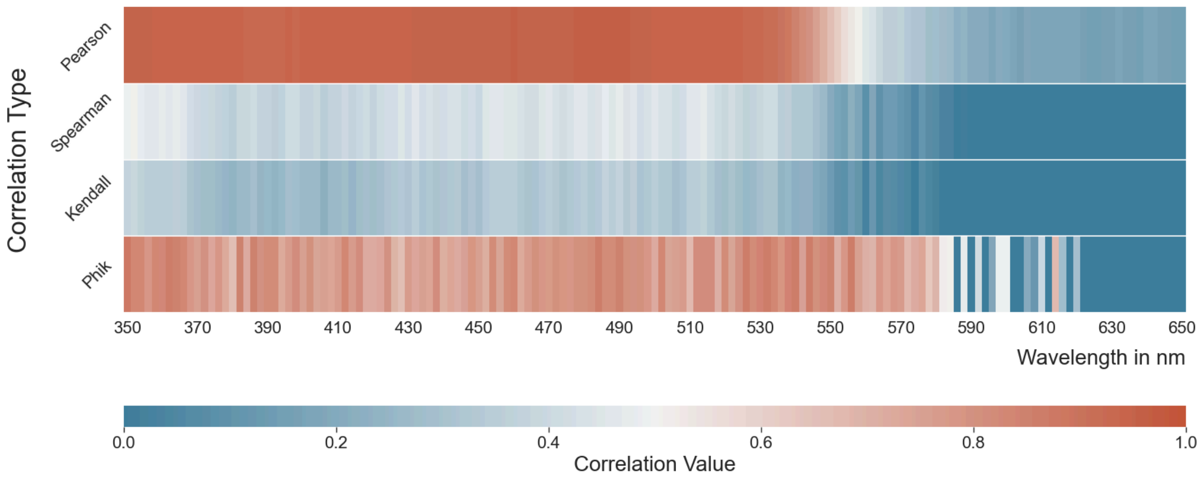

Figure S25 - Run 62 - Plate 01 - Correlations Between Dilution Ratio and Measurements

3.2 - Run 63 - Plate 01 - Manual

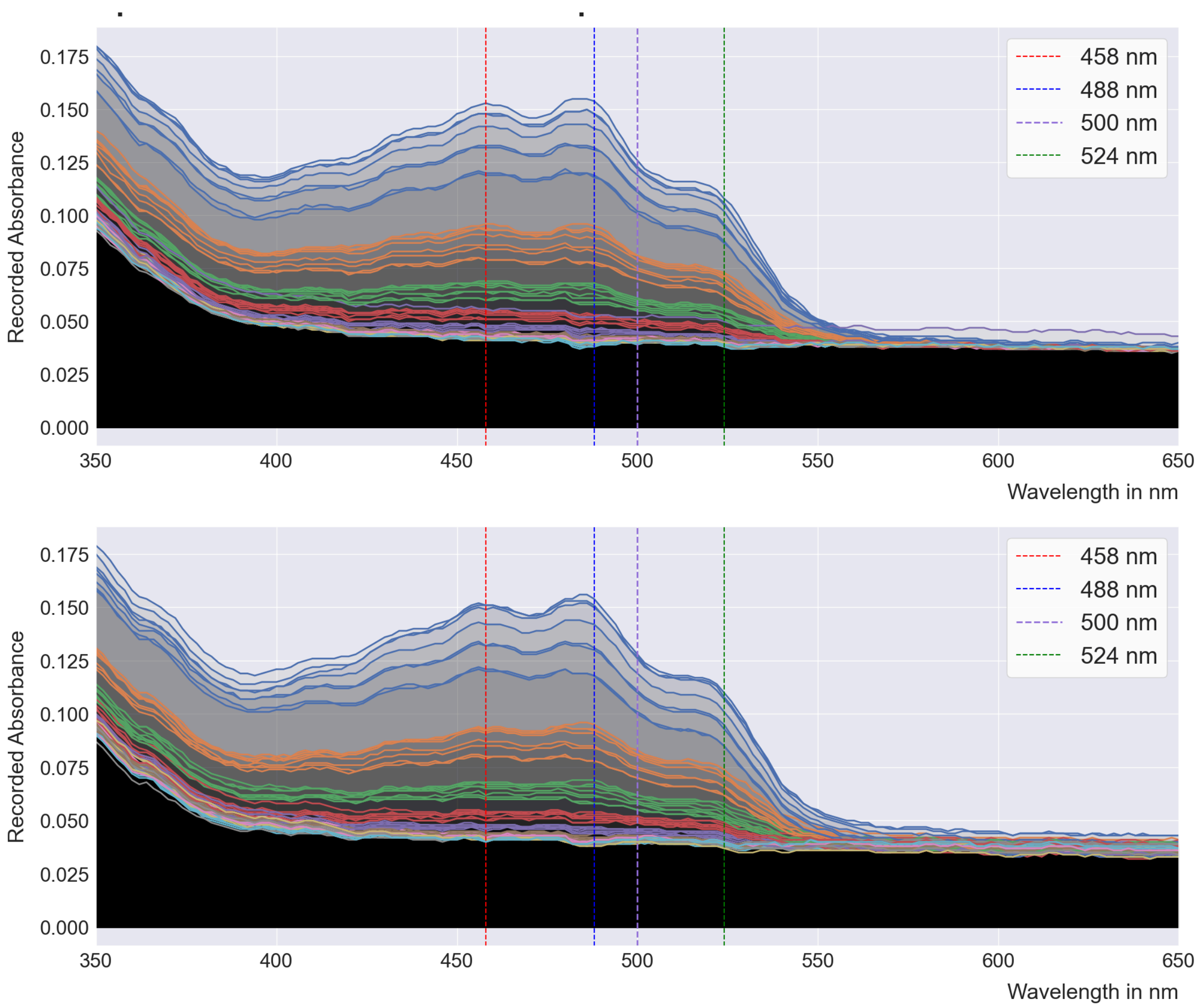

Figure S26 - Run 63 - Plate 01 - Absorbance Spectrum for All Samples.

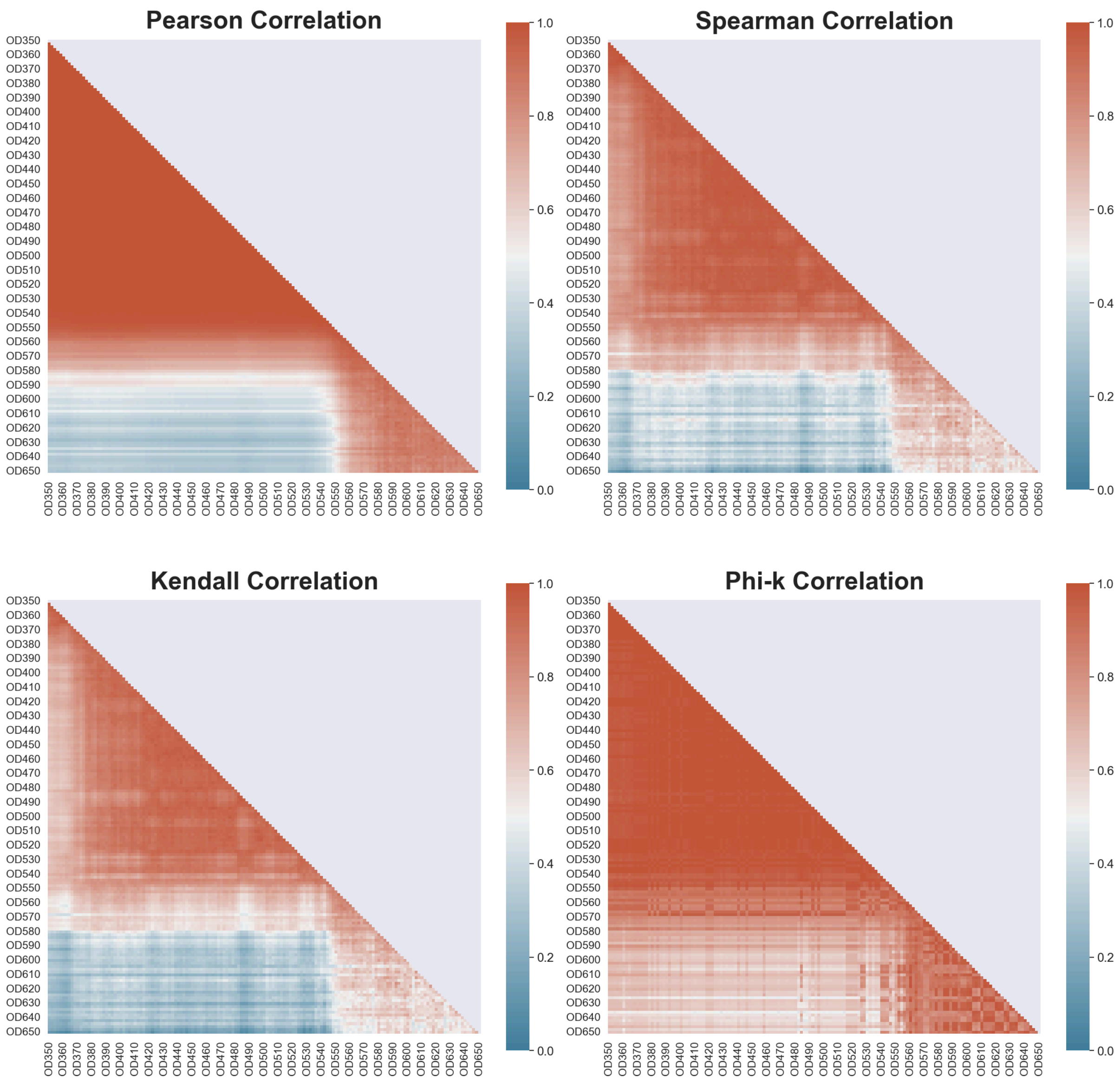

Figure S27 - Run 63 - Plate 01 - Correlation Matrices Between Measurements

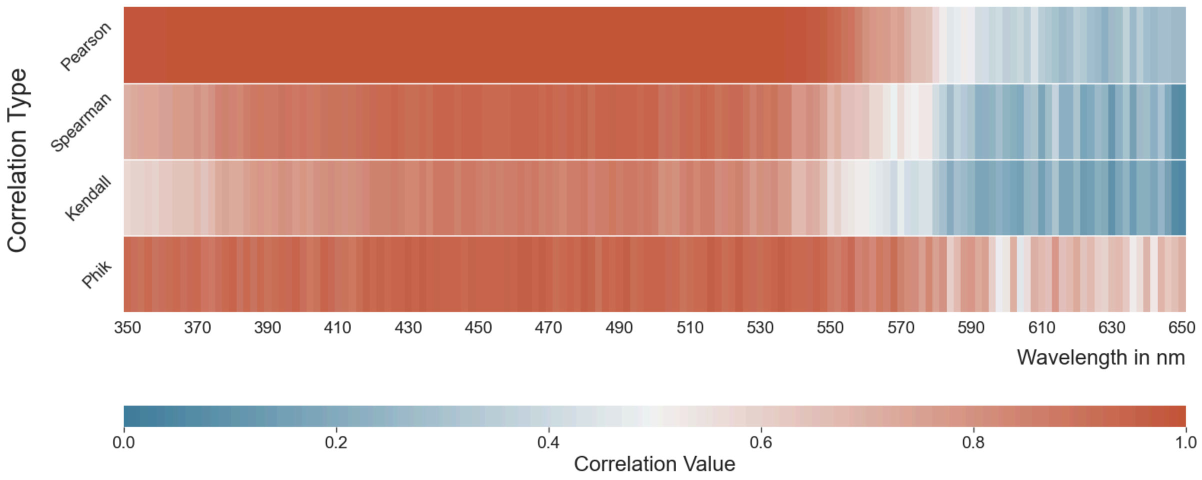

Figure S28 - Run 63 - Plate 01 - Correlations Between Dilution Ratio and Measurements

3.3 - Assessing the Effects of the Iterative Transfer

The stratified analysis was conducted on the data for DMSO and Lycopene. Results with the automated protocol varied greatly depending on the column – measurements were conducted in two Clariostar plate readers to be certain the effects were not caused by the measuring instrument. Results for the plate generated by manually were much smoother and closer to those with water and food colorant.

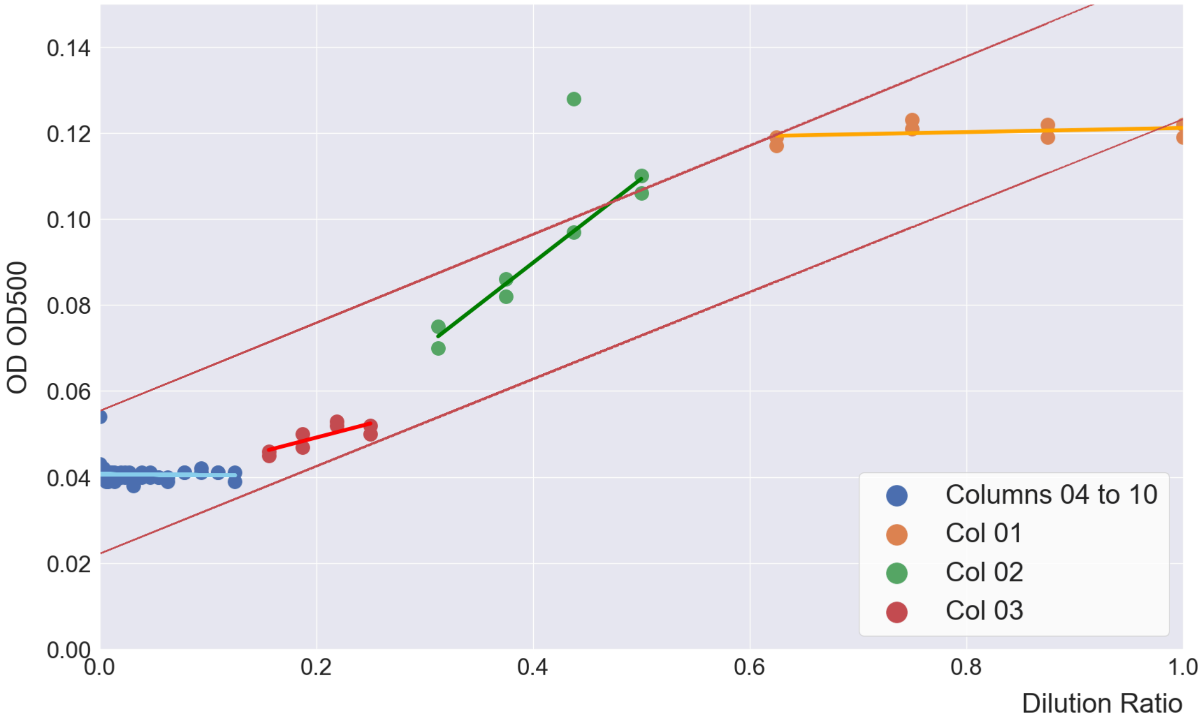

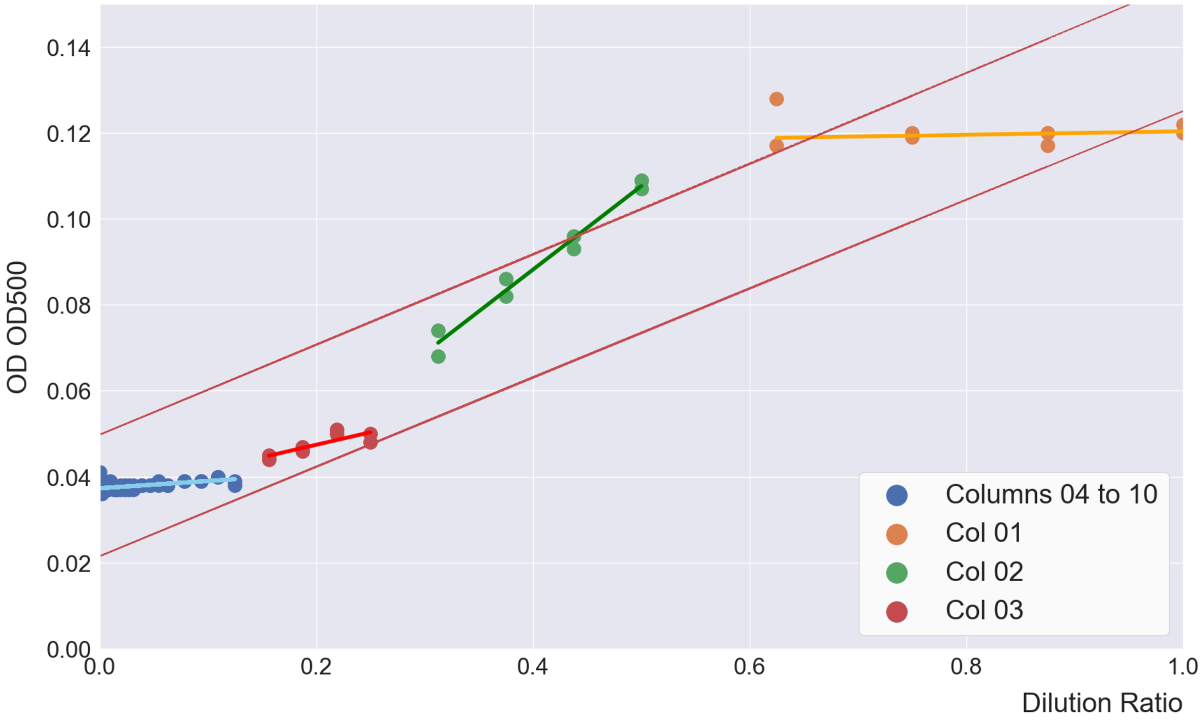

Figure S29 - Run 62 - Plate 01 (Automated) - Effects of the Iterative Dilutions at 500 nm
Top : Measurements in Clariostar A - Bottom in Clariostar B

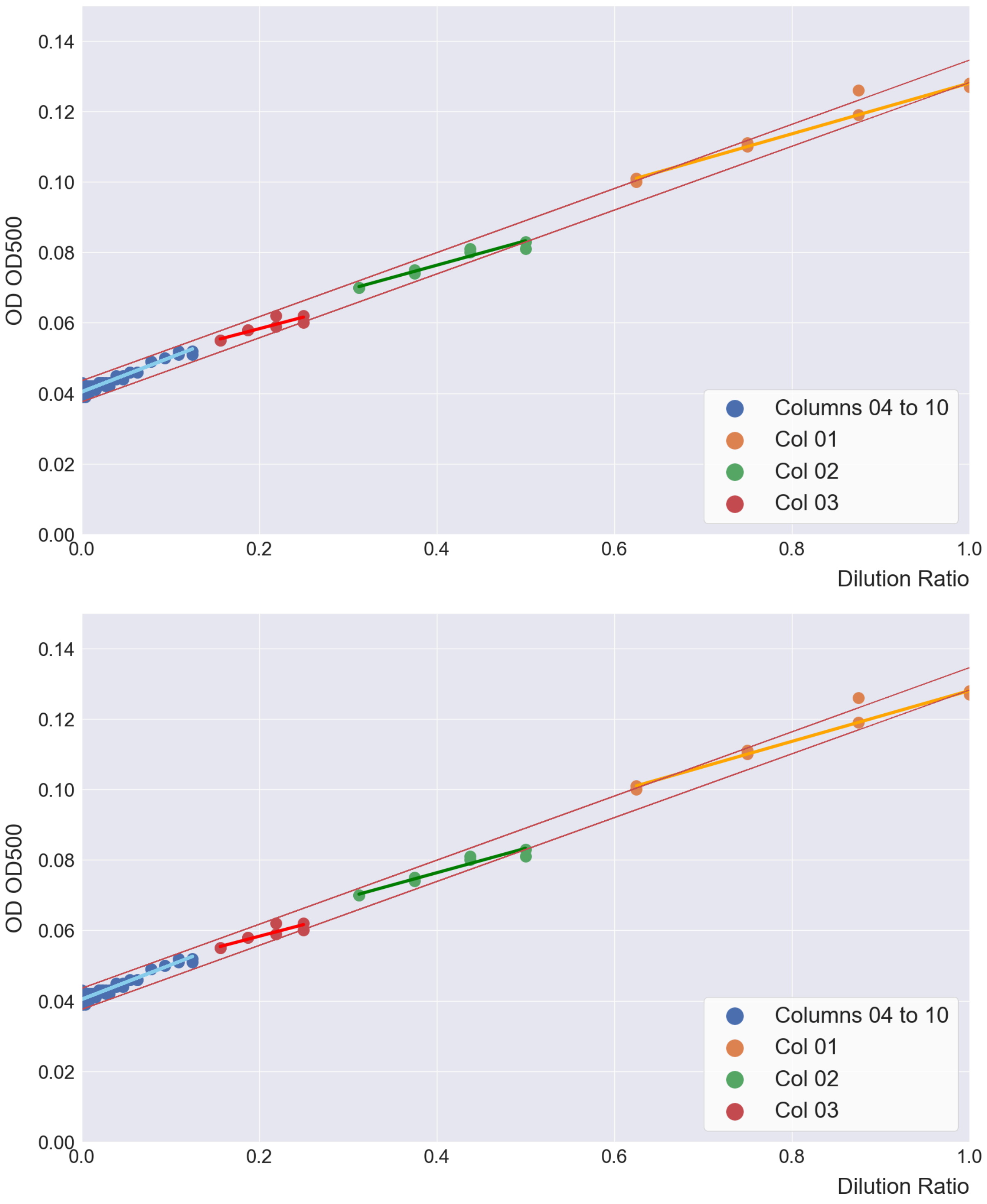

Figure S30 - Run 63 - Plate 01 (Manual) - Effects of the Iterative Dilutions at 500 nm
Top : Measurements in Clariostar A - Bottom: in Clariostar B

Other Low Concentration Mixes of Lycopene in DMSO

The same analysis was run on a set of runs using lycopene in DMSO at low concentration – the plates were only generally with the automated protocol. Again a great deal of variation could be observed from one column to the next, but also from one plate to another

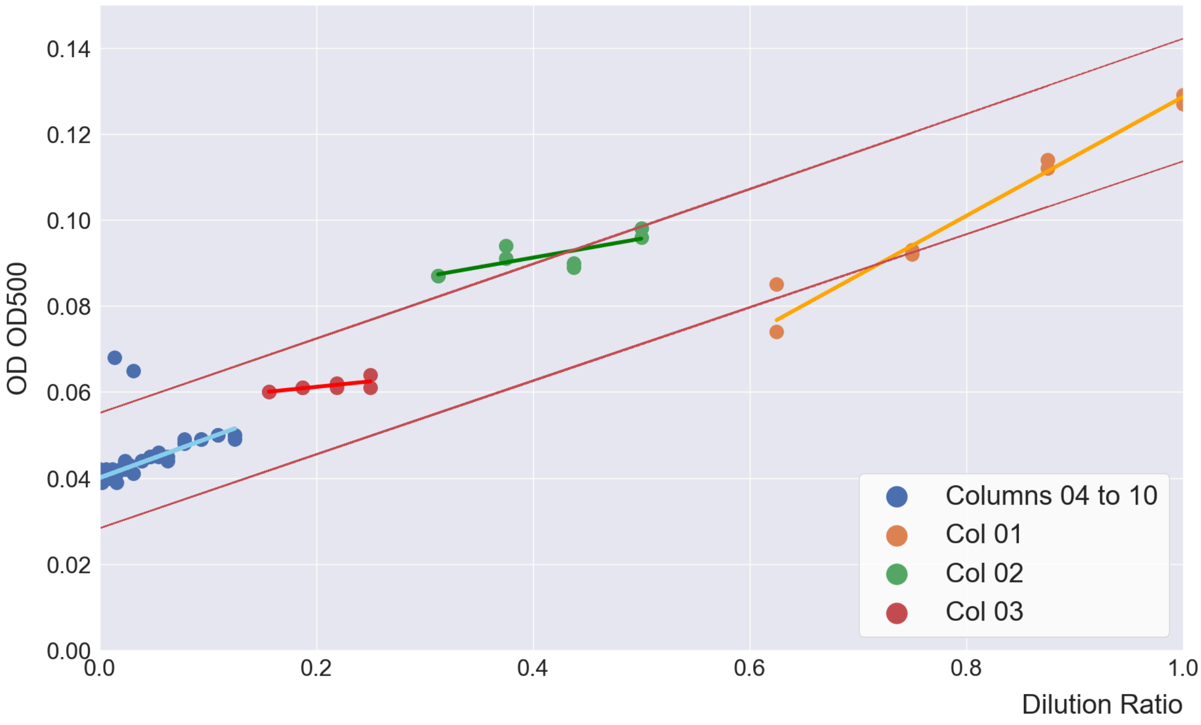

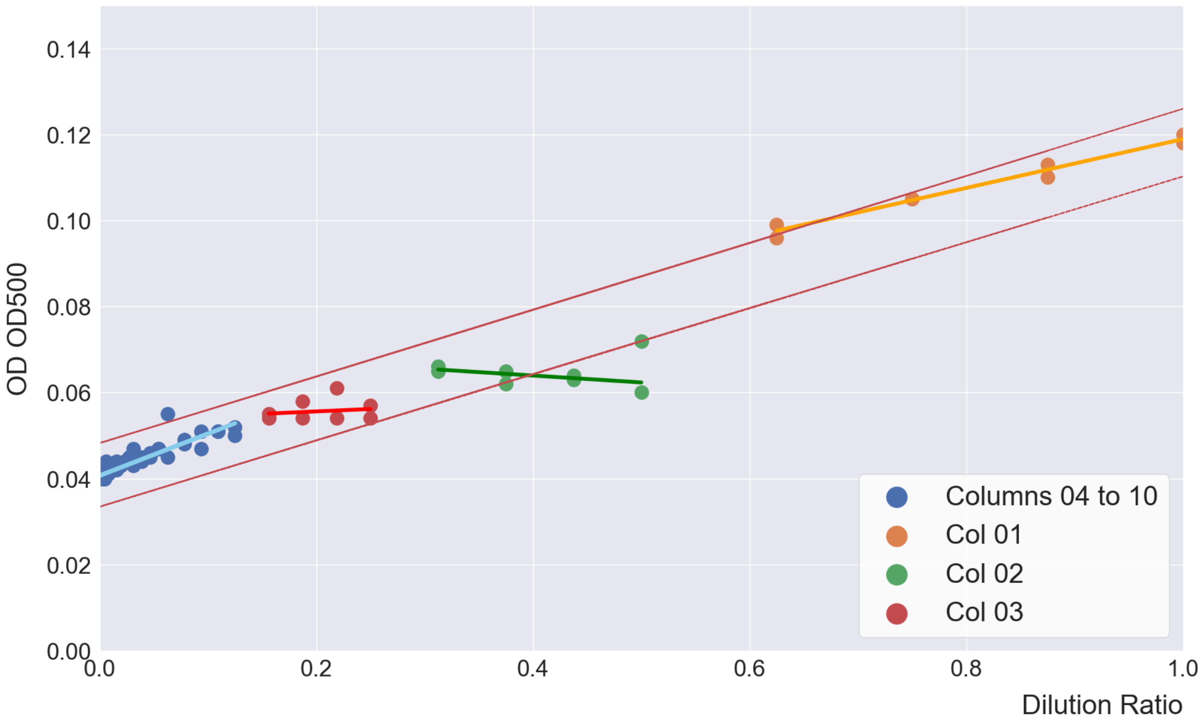

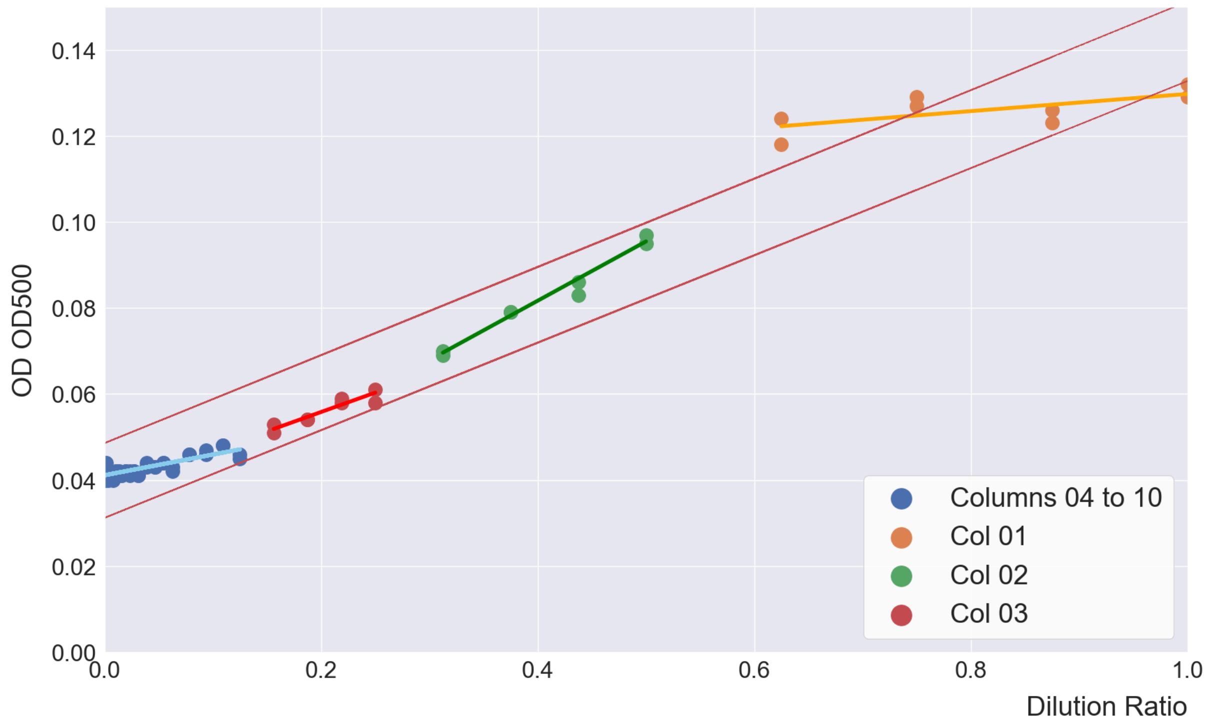

Figure S31 - Effects of the Iterative Dilutions at 500 nm for some Low Concentration Mixes. Top : Run 58 - Plate 01 - Middle : Run 64 - Plate 01 - Bottom : Run 61 - Plate 01

High Concentration Mixes of Lycopene in DMSO

Results for high concentration mixes (also for plates generated with automation) showed even more discrepancies from one column to the next (and wider confidence intervals).

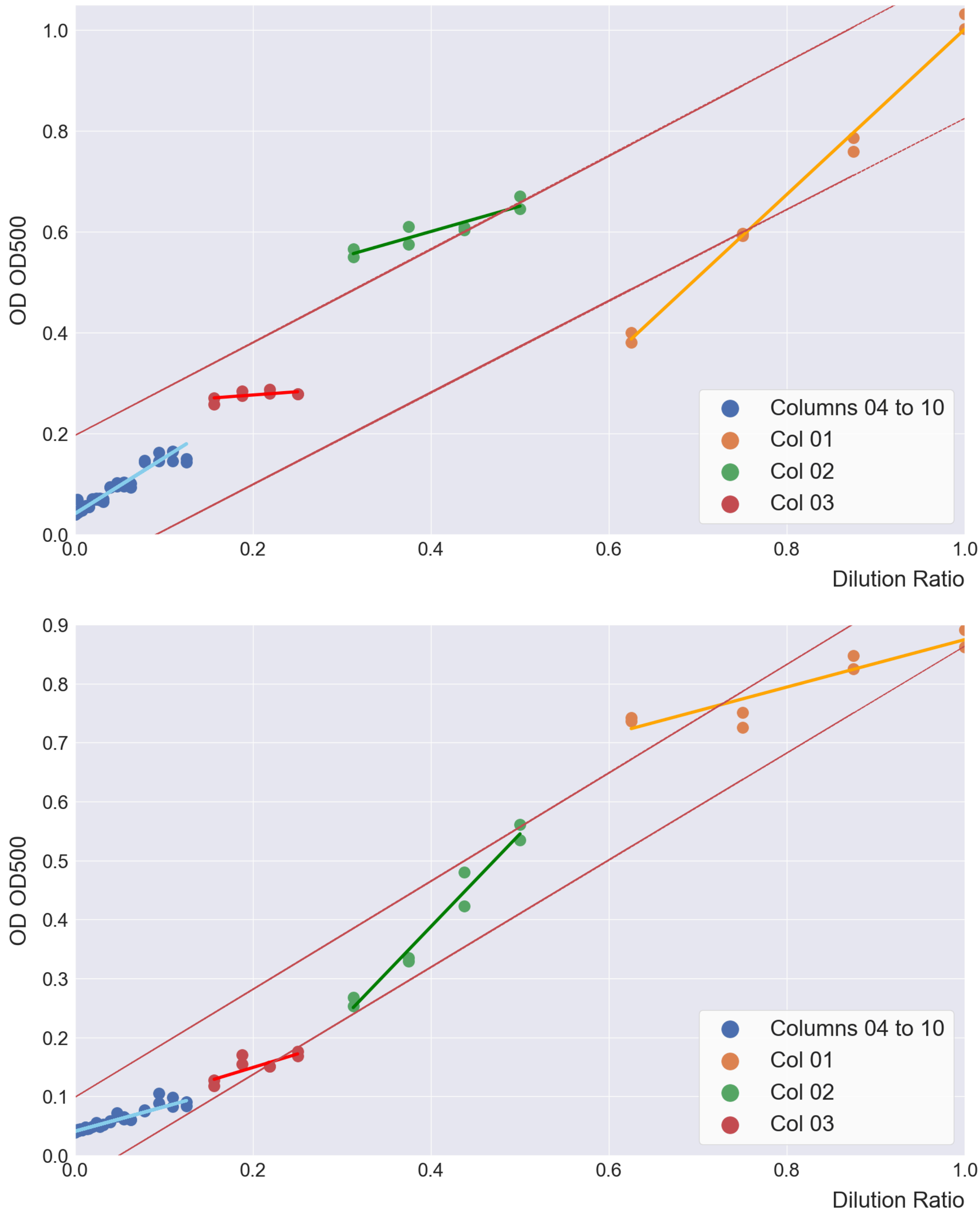

Figure S32 - Effects of the Iterative Dilutions at 500 nm (High Concentration Mixes).
Top : Run 59 - Plate 01 - Bottom : Run 60 - Plate 01

Stratification by Column - Variation of the Trend Lines

**Dataset**	**Trend** Whole	**Trend** Col 01	**Trend** Col 02	**Trend** Col 03
**55/01/A**	Y=1.0631X + 0.0383	Y=0.7272X + 0.3244	Y=0.8248X + 0.1236	Y=0.8256X + 0.0931
**55/02/A**	Y=1.0886X + 0.0380	Y=0.8448X + 0.2438	Y=0.8528X + 0.1320	Y=0.8672X + 0.08578
**57/01/A**	Y=1.032X + 0.0389	Y=0.6718X + 0.3406	Y=0.8109X + 0.1270	Y=0.952X + 0.0575
**57/02/A**	Y=1.0031X + 0.0397	Y=0.6784X + 0.3118	Y=0.88X + 0.0962	Y=0.987X + 0.0503

Table S3 - Food Colorant in Water Mixes - Column Trends Vs Dataset Trends. All parameters were rounded to the closer 4th decimal

**Dataset**	**Trend** Whole	**Trend** Col 01	**Trend** Col 02	**Trend** Col 03
**59/01/A**	Y=0.9173X + 0.0565	Y=1.6390X + -0.6371	Y=0.5024X + 0.3997	Y=0.1304X + 0.2504
**59/01/B**	Y=0.9585X + 0.0516	Y=1.4484X + -0.4397	Y=0.5617X + 0.3781	Y=0.1505X + 0.2392
**60/01/A**	Y=0.9133X + 0.0269	Y=0.4026X + 0.4721	Y=1.5712X + -0.2403	Y=0.4592X + 0.0574
**60/01/B**	Y=0.9161X + 0.0261	Y=0.320X + 0.5463	Y=1.4284X + -0.1954	Y=0.5086X + 0.0485

Table S4 - High Concentration Lycopene Mixes - Column Trends Vs Dataset Trends. All parameters were rounded to the closer 4th decimal

**Dataset**	**Trend** Whole	**Trend** Col 01	**Trend** Col 02	**Trend** Col 03
**62/01/A**	Y=0.1021X + 0.0388	Y=0.0048X + 0.1163	Y=0.1953X + 0.0117	Y=0.0656X + 0.0361
**62/01/B**	Y=0.1044X + 0.0356	Y=0.0040X + 0.1163	Y=0.194X + 0.0104	Y=0.0582X + 0.0358
**63/01/A**	Y=0.0899X + 0.0403	Y=0.0633X + 0.0630	Y=0.0605X + 0.0516	Y=0.0544X + 0.0472
**63/01/B**	Y=0.0908X + 0.04056	Y=0.0720X + 0.0560	Y=0.0693X + 0.0486	Y=0.0626X + 0.04507
**58/01/A**	Y=0.0861X + 0.0417	Y=0.1384X + -0.0098	Y=0.0443X + 0.0735	Y=0.0256X + 0.0561
**58/01/B**	Y=0.0867X + 0.0419	Y=0.1260X + 0.0025	Y=0.0490X + 0.0707	Y=0.0209X + 0.0575
**64/01/A**	Y=0.0772X + 0.0408	Y=0.057X + 0.0621	Y=-0.016X + 0.0703	Y=0.0113X + 0.0533
**64/01/B**	Y=0.0773X + 0.0408	Y=0.0568X + 0.0621	Y=-0.016X + 0.07033	Y=0.0113X + 0.05334
**61/01/A**	Y=0.1021X + 0.0399	Y=0.02X + 0.1097	Y=0.13783X + 0.0265	Y=0.09004X + 0.03781
**61/01/B**	Y=0.1032X + 0.0385	Y=0.0278X + 0.1026	Y=0.1493X + 0.0212	Y=0.0992X + 0.0353

Table S5 - Low Concentration Lycopene Mixes - Column Trends Vs Dataset Trends. All parameters were rounded to the closer 4th decimal

4 - Linear Dilution Scheme - Preliminary Studies

4.1 - Development Runs - Food Colorant and DMSO

We present the results for Run 74 - Protocol 2.0.3 (minor alteration of the speed of aspiration from DMSO reservoir; all automated implementations of the linear scheme are denoted protocol 2.X.X). Runs 70,74 and 75 were used for the development phase.

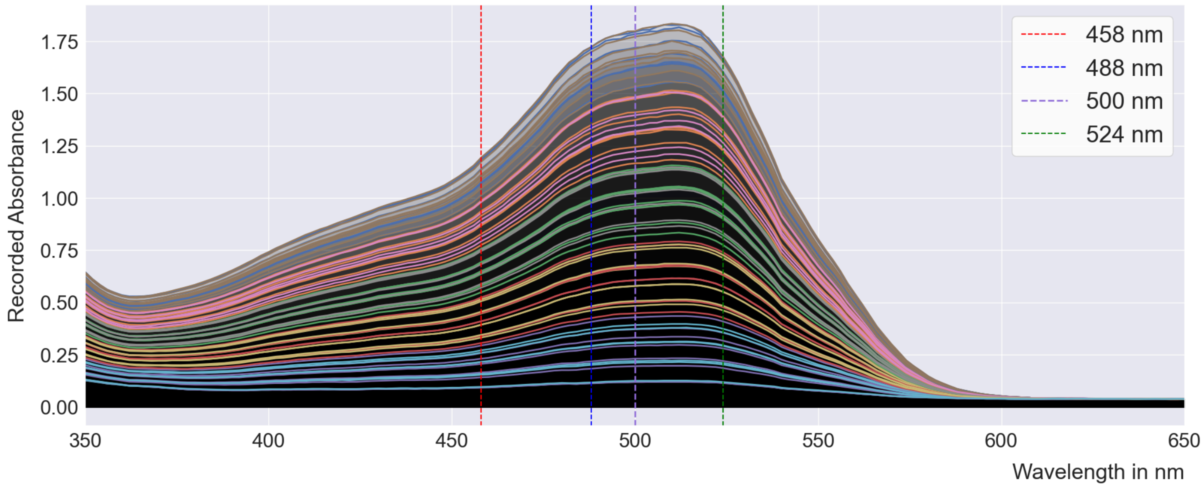

Figure S33 - Run 74 - Plate 01 - Absorbance Spectrum for All Samples

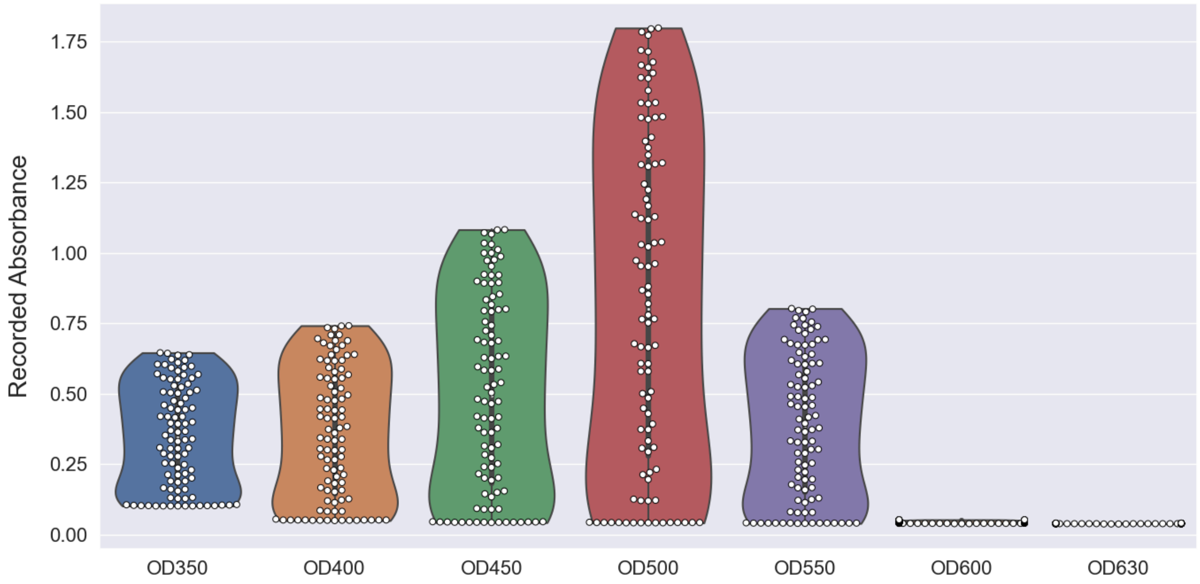

Figure S34 - Run 74 - Plate 01 - Corresponding Violin Plot at Selected Wavelengths
The violin plot for the measurements is not as lopsided as for the geometric scheme - as expected from the linear distribution of the concentrations.

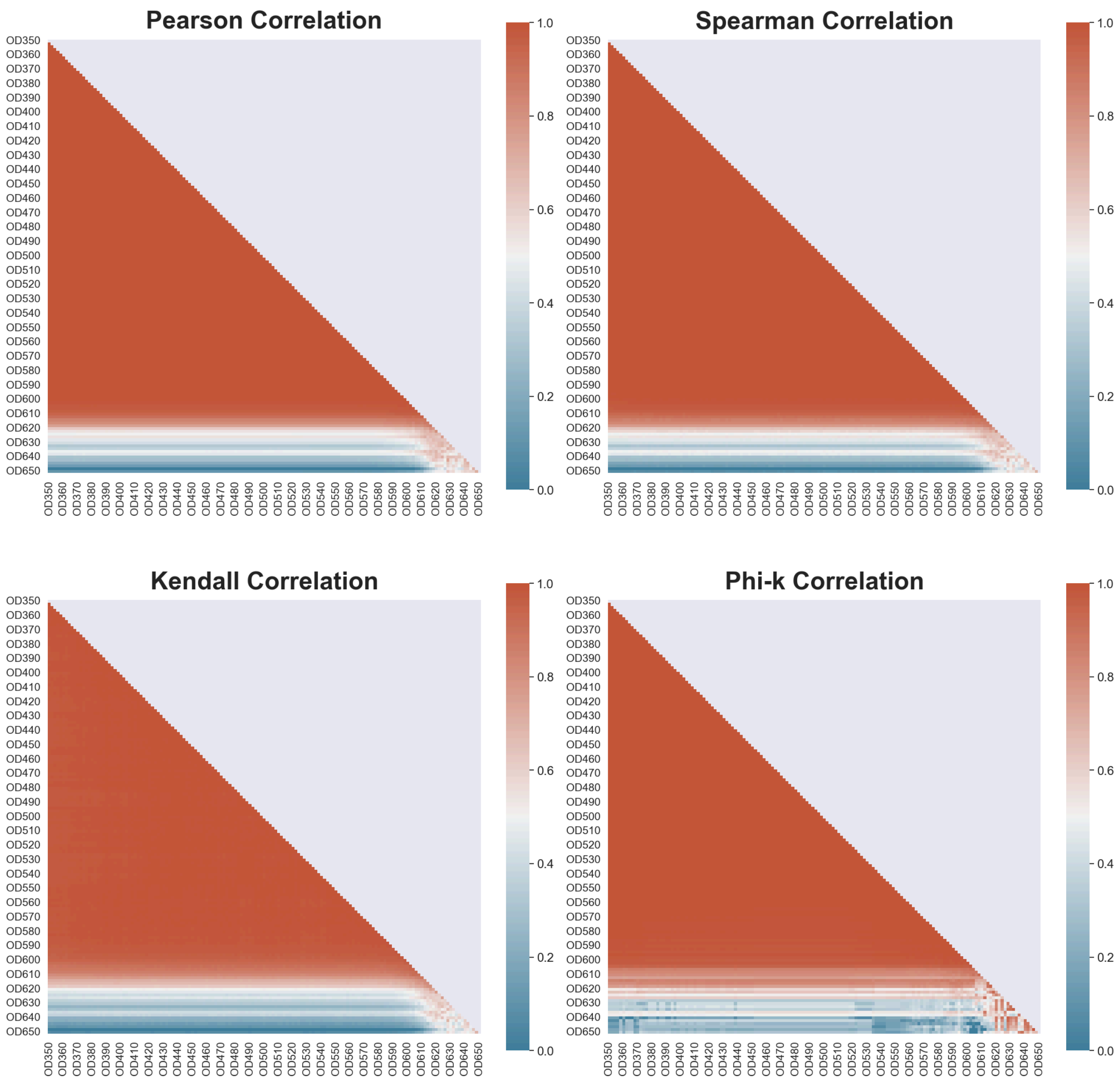

Figure S35 - Run 74 - Plate 01 - Correlation Matrices Between Measurements

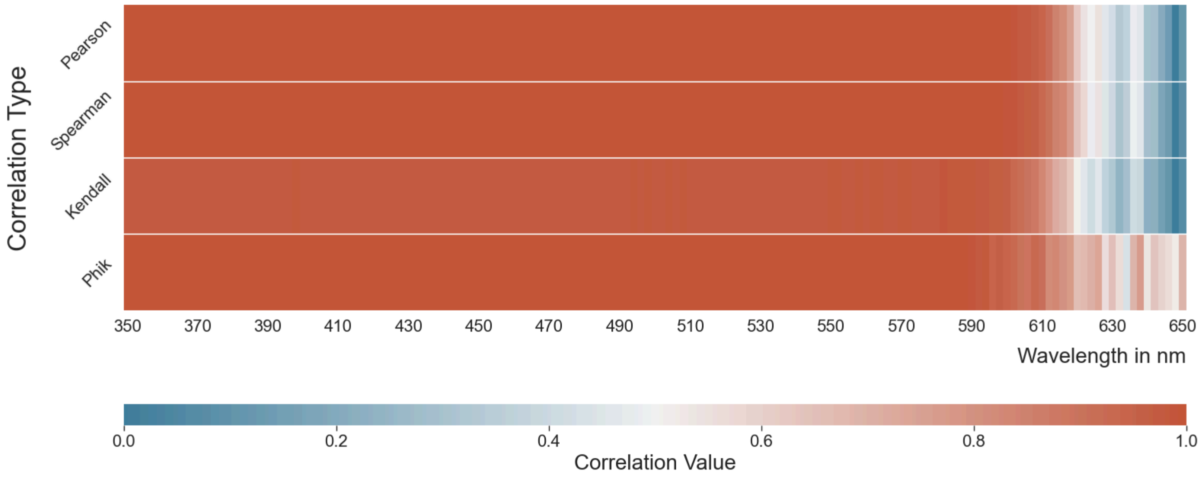

Figure S36 - Run 74 - Plate 01 - Correlations Between Dilution Ratio and Measurements

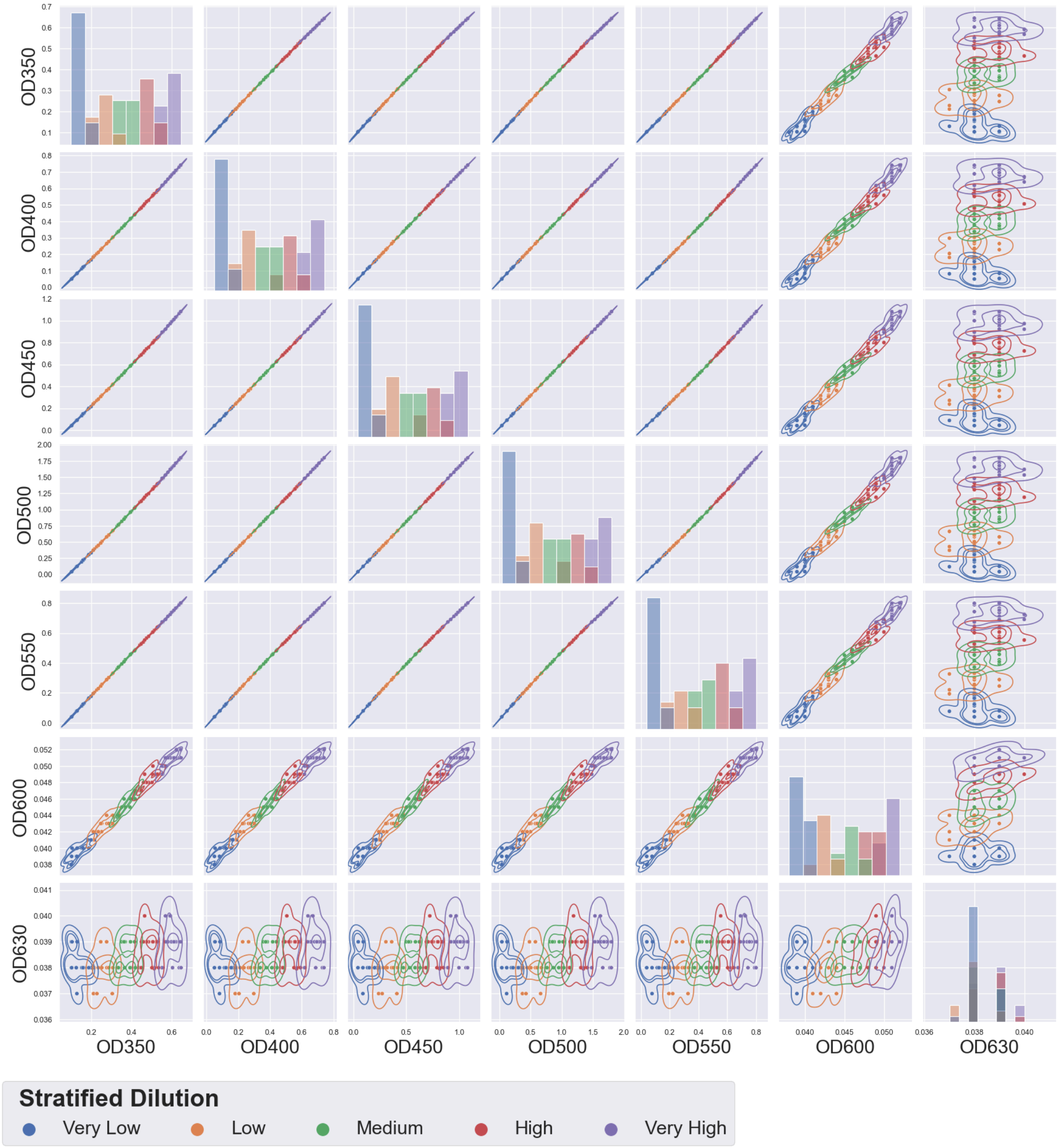

Figure S37 - Run 74 - Plate 01 - Pairplot Between Measurements at Selected Wavelengths

4.2 - Stratified Analysis

The measurement data were stratified by columns and by rows.

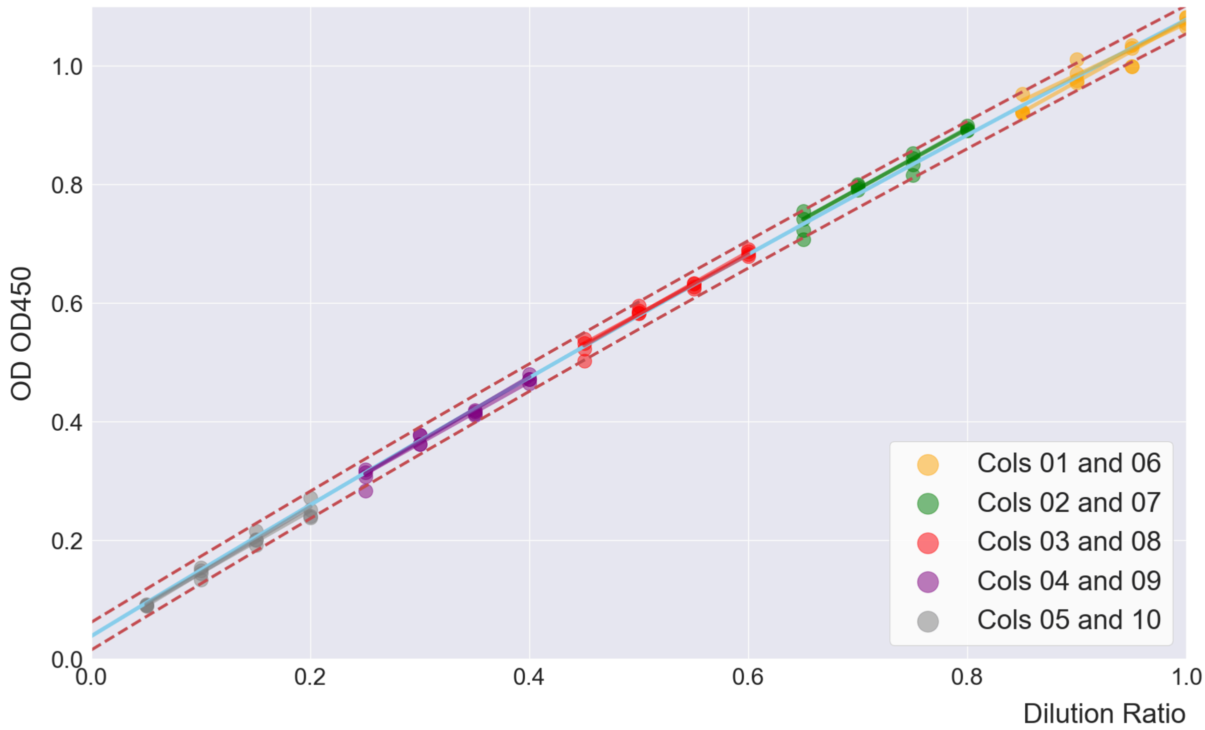

Figure S38 - Run 74 - Plate 01 – Stratification by Column of the Measurements at 450 nm

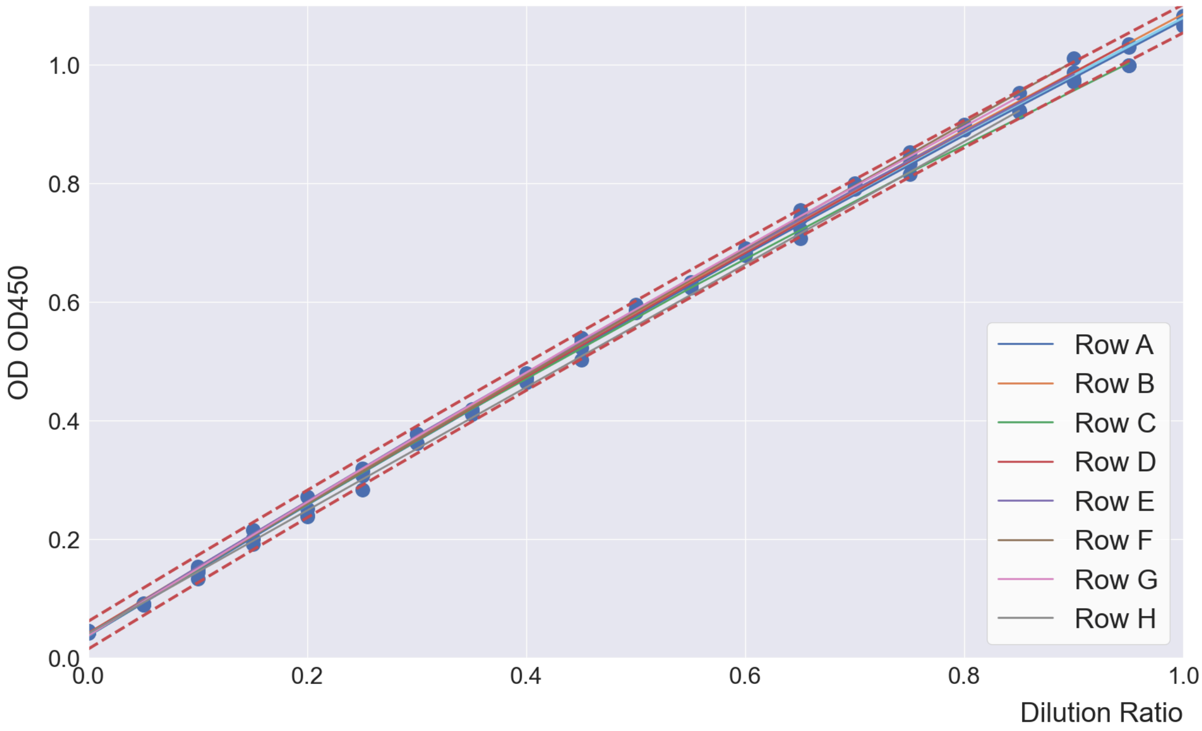

Figure S39 - Run 74 - Plate 01 – Stratification by Row of the Measurements at 450 nm

5 - Main Analytical Methods

5.1 - Main Analytical Approach

Take data at a given wavelength (here 450 nm) and apply the following three steps

Step 1 - Two models were used for the regression: a liner model and a quadratic model to capture what curvature is in the data. A linear regression was conducted on the dataset (in blue, confidence interval is displayed in red), and a robust linear model approach (in green) was also run – weighing the data points so the outliers are less prominent.

Step 2 - After inversion of the regression models, the real dilution ratios are estimated for all the samples from their measurement. When plotted against the targeted dilution, they should fall on the Y=X line. Global (quality) metrics are ten computed that quantify how well they fall on said line – in practice we use the Pearson correlation (to quantify the linear relationship) and the mean square error (or equivalent in our case the square root of it) to quantify the magnitude of the residuals (how far the points fall off the line).

	**Model** Order 1	**Model** Order 2
**Pearson Corr**	0.9992	0.9994
**Spearman Corr**	0.9974	0.9974
**RMSE**	0.01295	0.0111

Table S6 - Global Quality Metrics for Run 74. All values rounded to the fourth decimal

These metrics can be used as global quality metrics - the higher the correlation, the better the model is at explaining the data. Deviations from the trend

For all samples, The residual errors from the predictions can be turned into a relative error (residual/expected dilution value), and displayed on a plate map.

Results for the training runs with Food Colorant in DMSO yielded very tight regression and relative errors of a few percent except for the lowest dilution ratios/concentrations. The quadratic model yielded minor improvements – mostly visible at these low dilutions

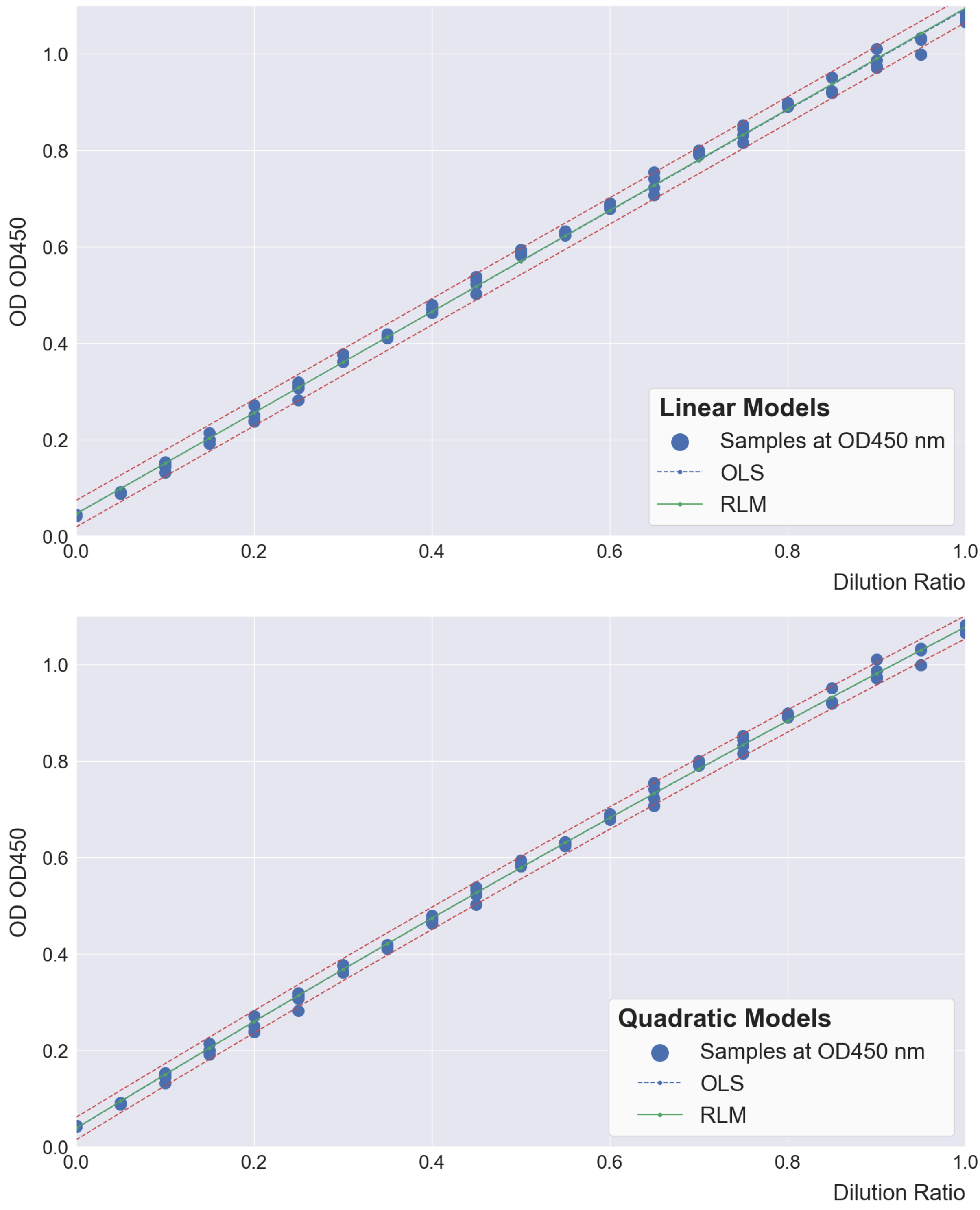

Figure S40 - Regression on the Measurements at 450 nm (Top = Linear Model, Bottom= Quadratic Model). Note the slight bend for the highest dilution ratios indicative of some saturation.

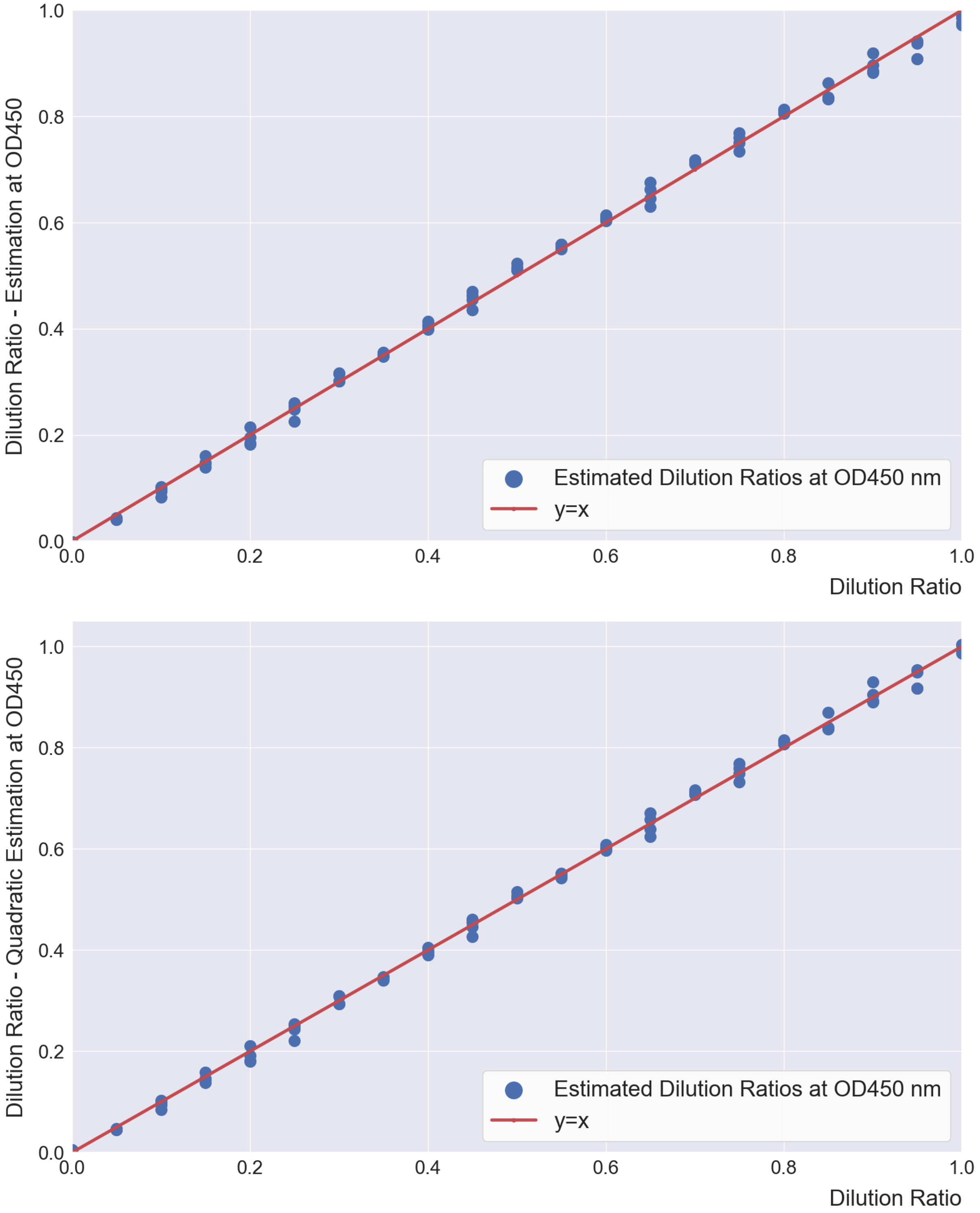

Figure S41 - Inversion of the Regression Models and Estimation of the Real Dilution Ratio (Top = Linear Model, Bottom= Quadratic Model)

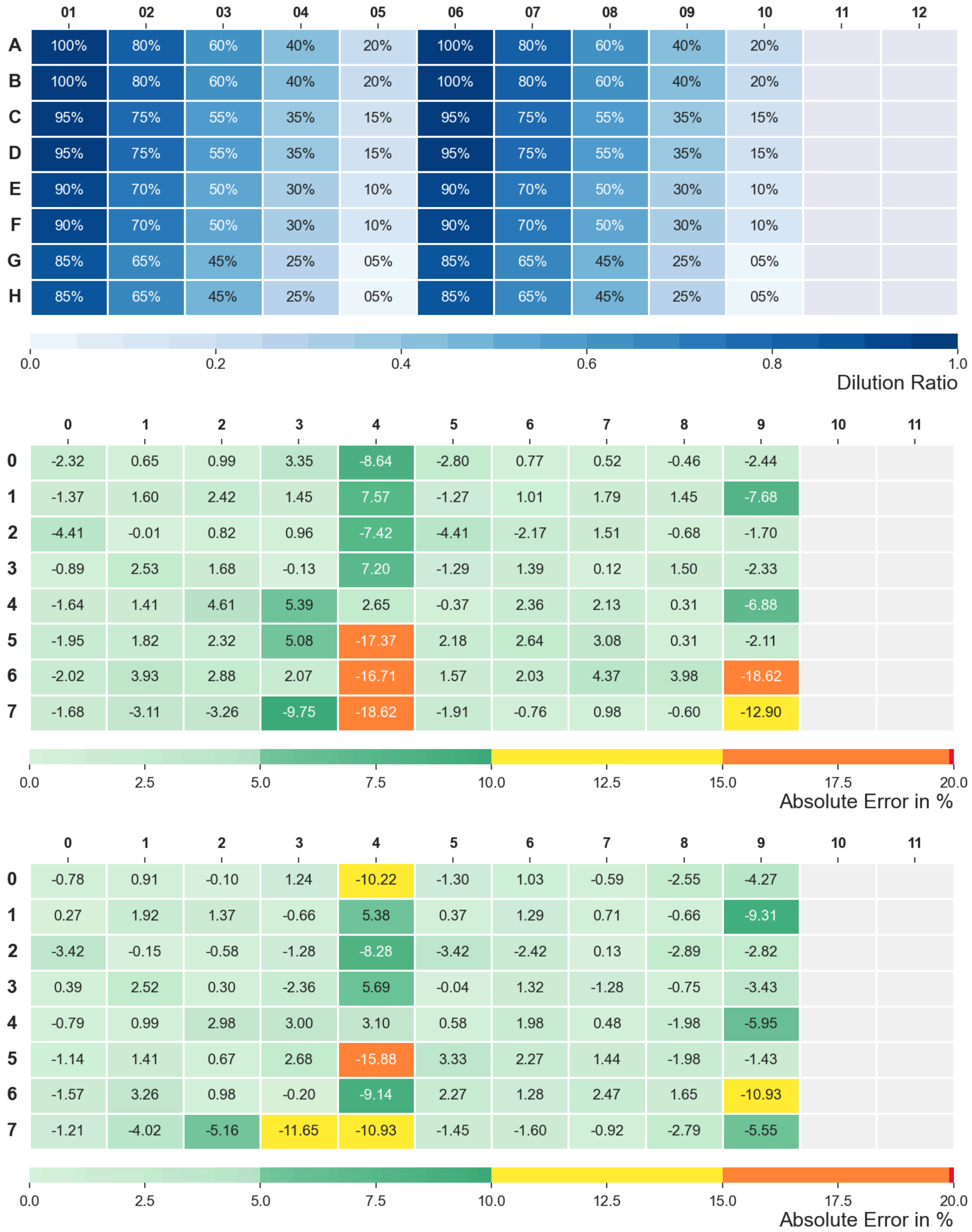

Figure S42 – Relative Errors Mapped onto the Plate (Run 74, Measurements at 450 nm). Top: Dilution ratios targeted by the linear dilution scheme; Middle: Estimated relative errors (linear model); Bottom: Estimated relative errors (quadratic model)

5.2 - Spectrum-Based Approach

The high degree of linear correlation between measurements at different wavelengths led to the construction of a model (called SM, for spectrum model) which binds measurements for any well over a range of wavelengths to a vector S - the spectrum of the solute - and a baseline B - the background measurement of the plate over the same range. The coefficient αi is well dependent and indicates the quantity of signal producing-solute in the well.

(SM) $Y_i = \alpha_i \times S + B$

The parameters B,S (αi not shown) are estimated at once from the plate data (under constraints that all components of S and B and coefficients αi.be positive and for penalty function that the baseline B stay close to blank values). The baseline B was plotted in red - and matches the absorbance spectrums of the blanks on the plate. The spectrum S , was plotted in amber; and for legibility purposes its amplitude was normalized so its value is 1 at wl= 400 nm. For all wells, the spectrum is reconstructed from the estimated parameters and plotted in black. As can be seen on Figure S43, the match between data and reconstruction is very tight.
The previous regression-inversion-error estimation workflow can then be applied to the coefficients αi

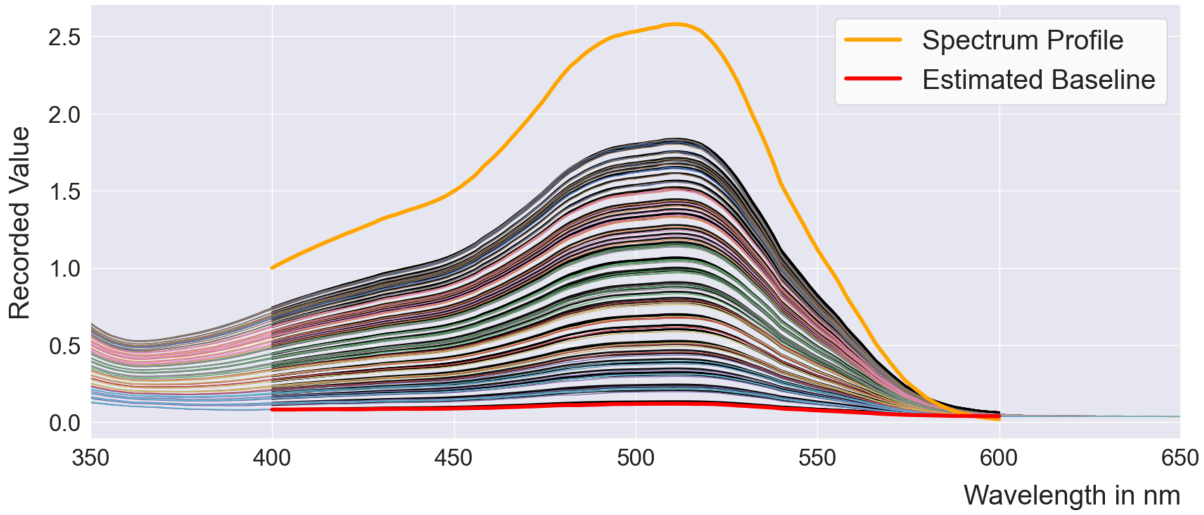

Figure S43 - Run 74 - Reconstructed Spectrum, Baseline and Absorbance Spectra

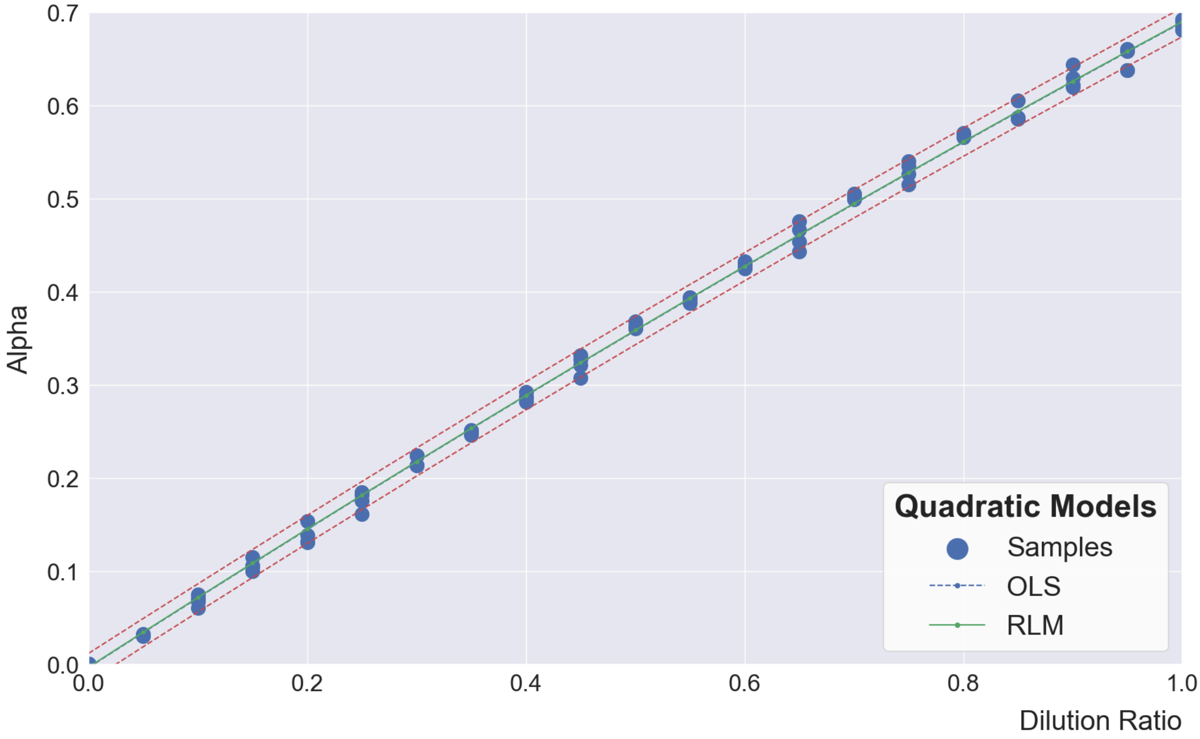

	0	1	2	3	4	5	6	7	8	9	10	11
0	-0.73	0.87	-0.11	1.30	-9.80	-1.39	1.16	-0.55	-2.62	-4.40		
1	0.13	1.79	1.34	-0.81	5.74	0.36	1.32	0.62	-0.62	-9.18		
2	-3.31	-0.34	-0.51	-1.39	-8.12	-3.36	-2.54	0.11	-2.81	-2.49		
3	0.40	2.44	0.23	-2.44	5.88	0.03	1.28	-1.37	-0.71	-3.39		
4	-0.77	0.90	2.82	2.97	3.94	0.63	1.87	0.52	-2.01	-5.69		
5	-1.06	1.41	0.65	2.91	-15.14	3.13	2.16	1.52	-1.93	-1.58		
6	-1.48	3.23	1.02	0.06	-8.89	2.14	1.20	2.55	1.63	-9.91		
7	-1.20	-4.08	-5.09	-11.39	-10.57	-1.39	-1.66	-0.95	-3.09	-4.87		

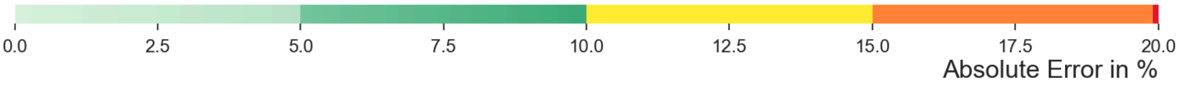

Figure S44 - Run 74 - Error Estimation Workflow for the Spectrum Method
Top: Regression of the coefficients αi (quadratic model) – Bottom: Relative errors (quadratic model)

6 - Linear Dilution Scheme - Lycopene in DMSO

6.1 – Development Runs Lycopene-DMSO

Several development runs for the linear dilution scheme were conducted - to tune up the protocol and test the error assessment framework.

Run 81 - Low concentration

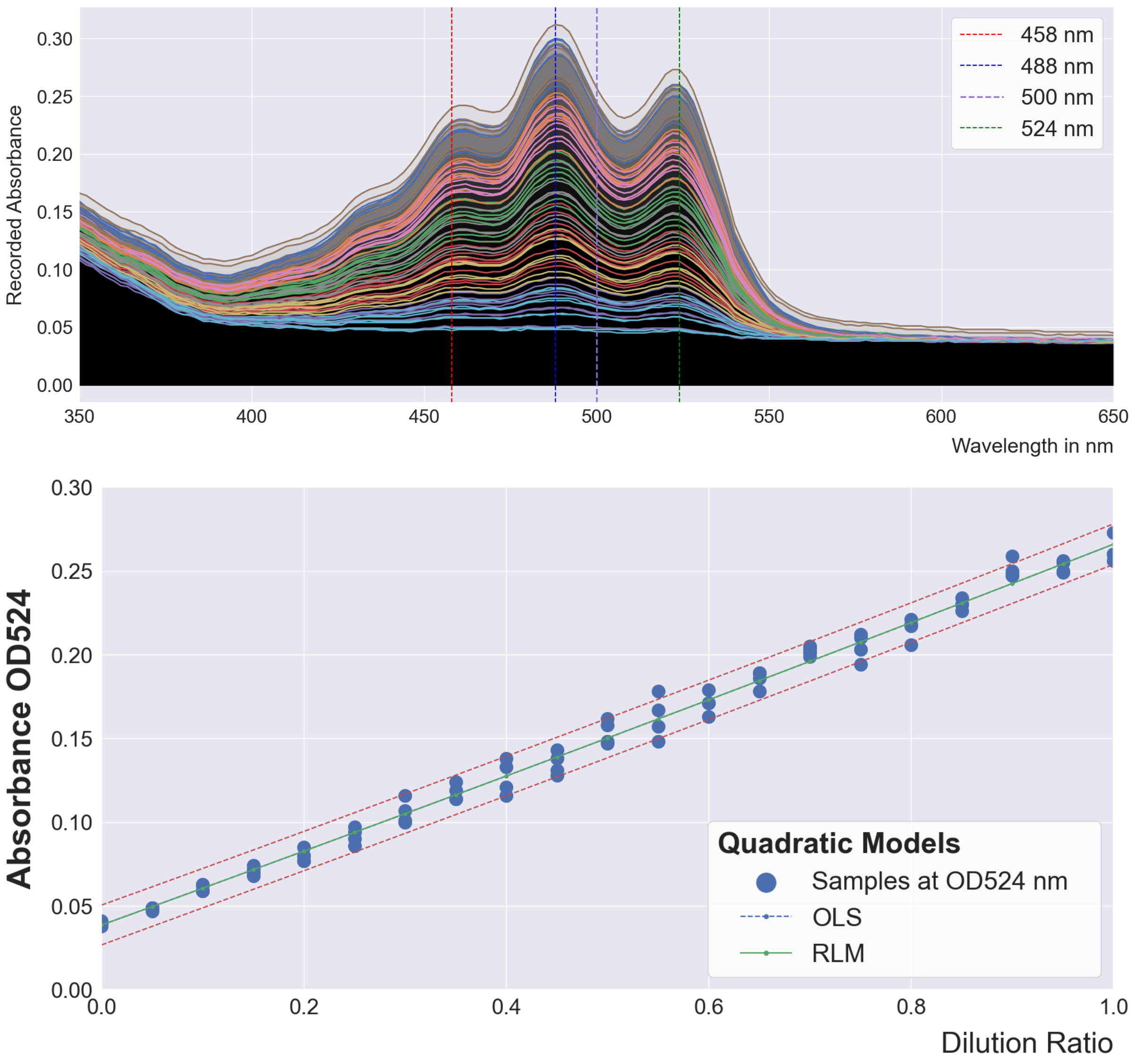

Figure S45 - Run 81 – Absorbance Spectrum and Regression at 524 nm

Run 80 - Low concentration

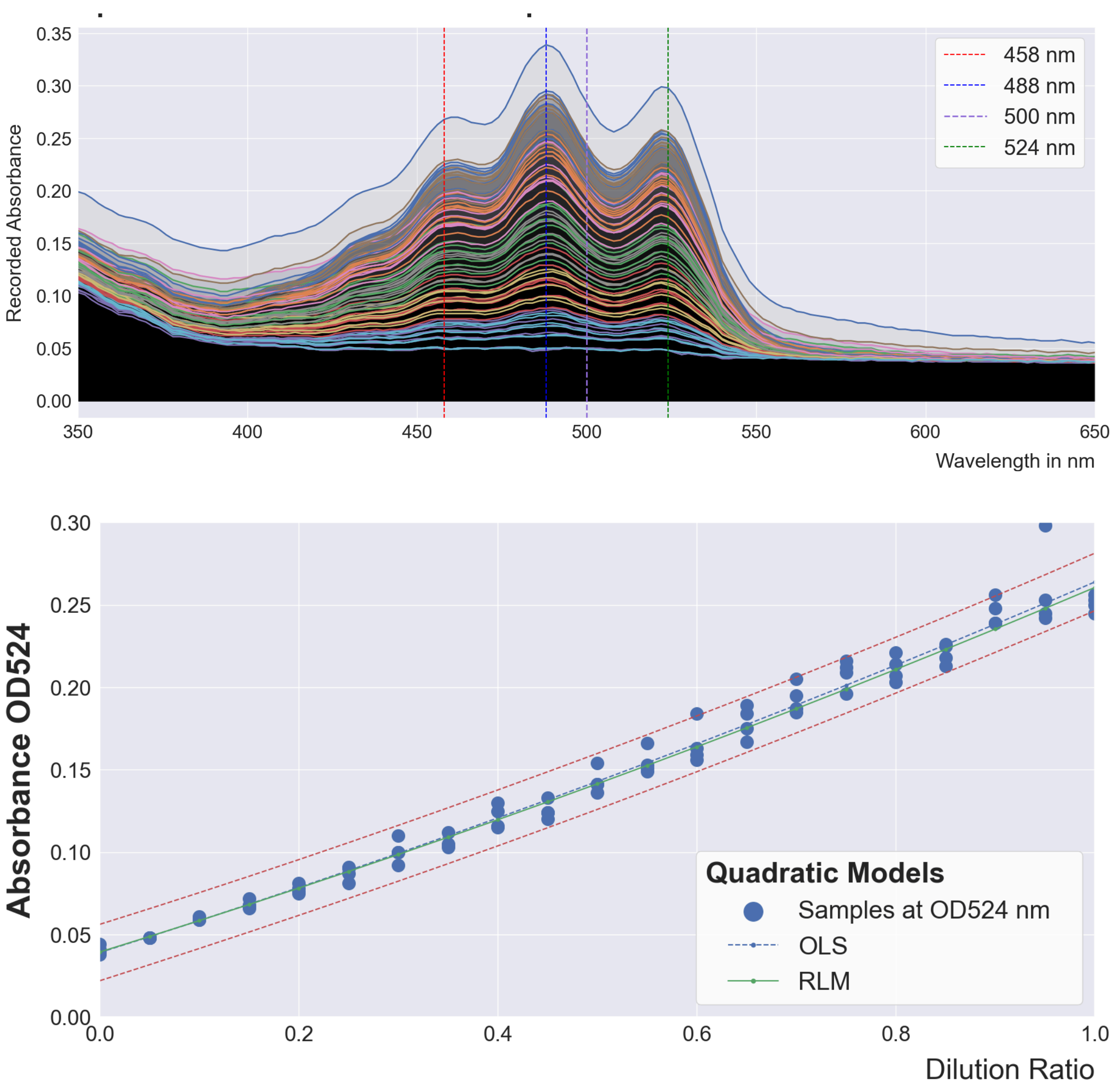

Figure S46 - Run 80 – Absorbance Spectrum and Regression at 524 nm

Run 82 - Low concentration

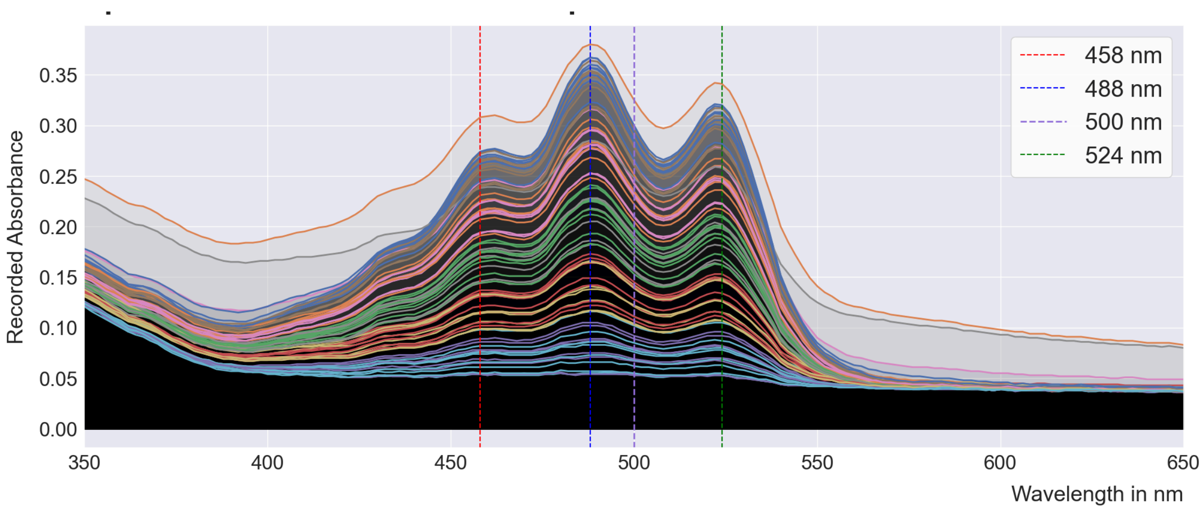

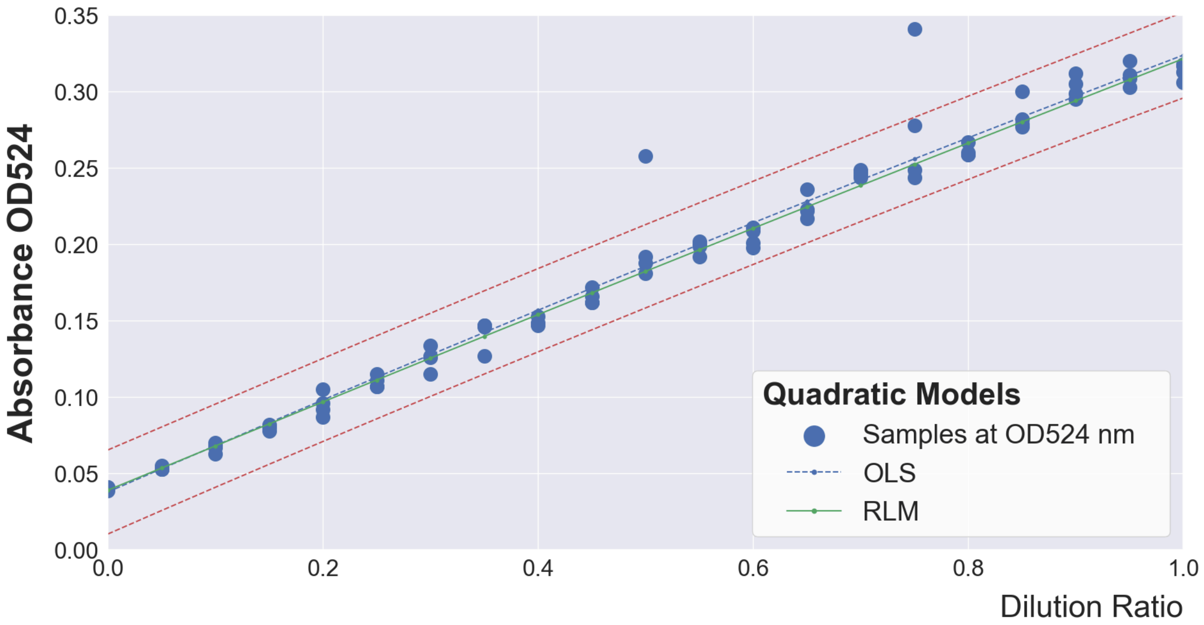

Figure S47 - Run 82 – Absorbance Spectrum and Regression at 524 nm. Note the outliers

Run 76 – High Concentration Mix

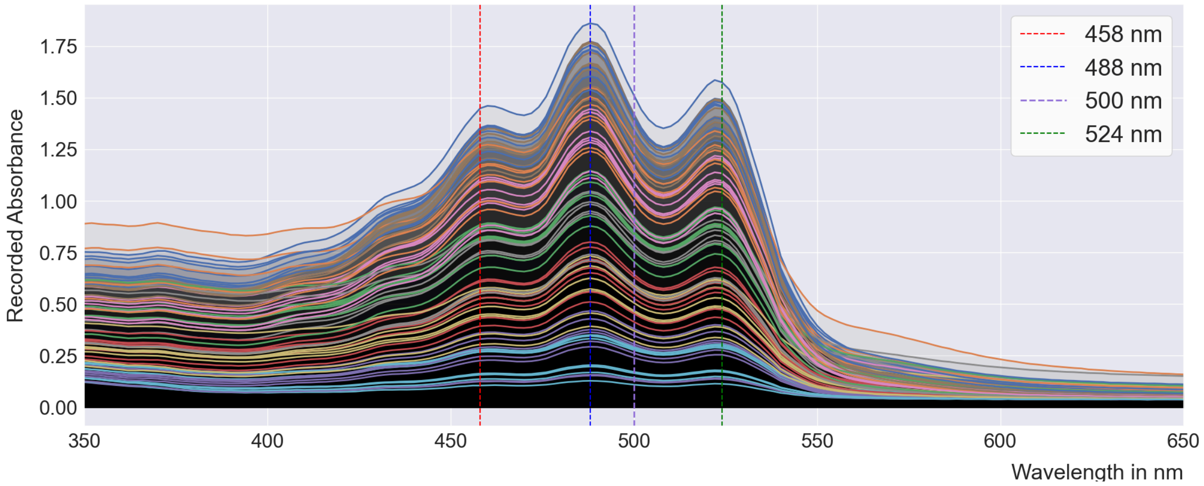

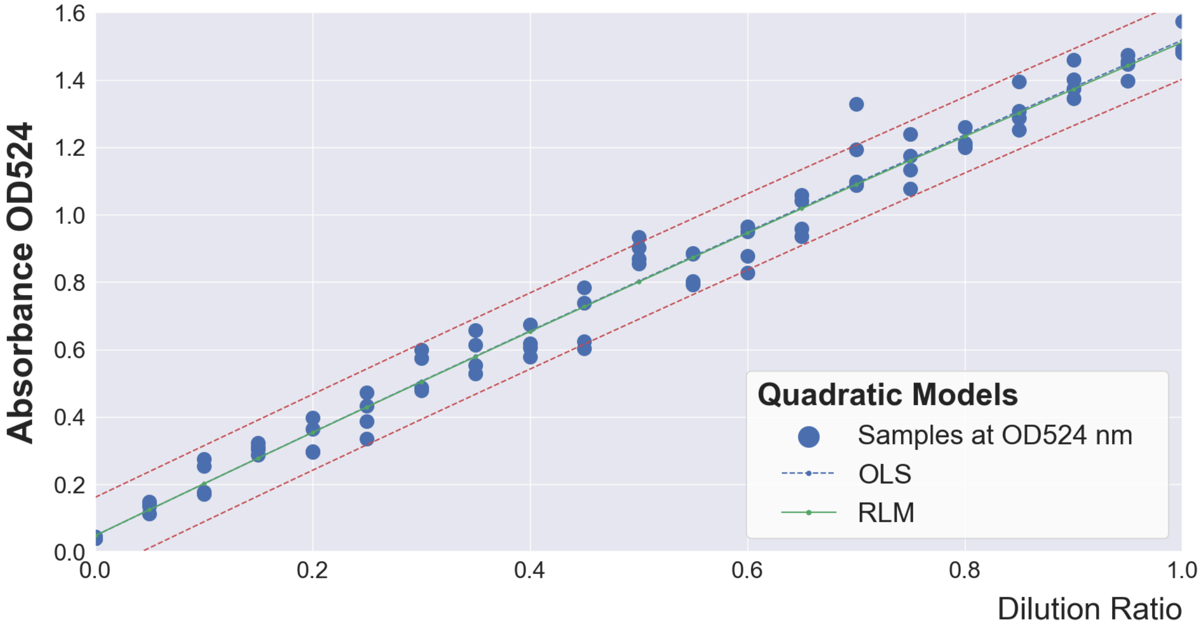

Figure S48 - Run 76 – Absorbance Spectrum and Regression at 524 nm

Run 79 – High Concentration Mix

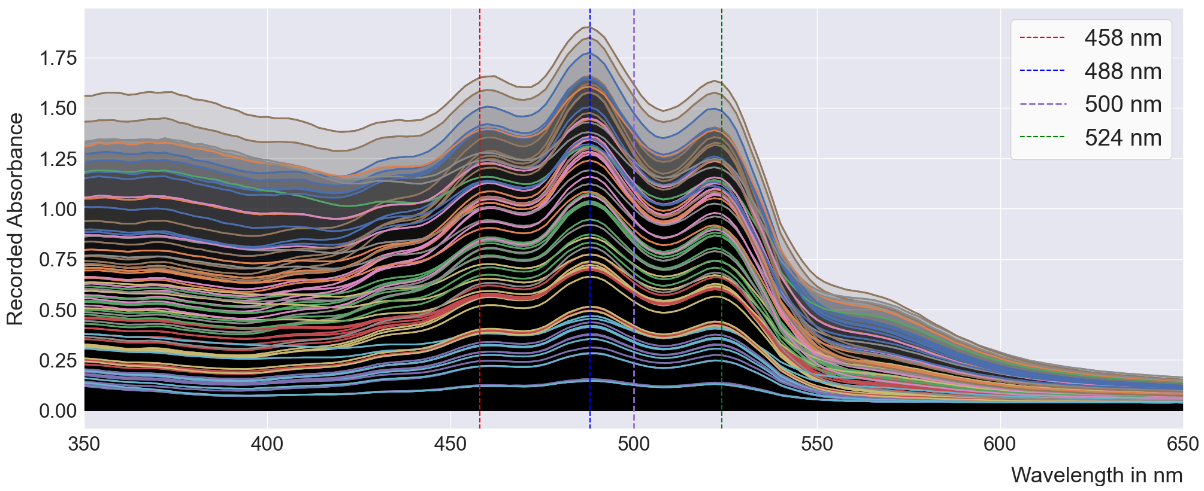

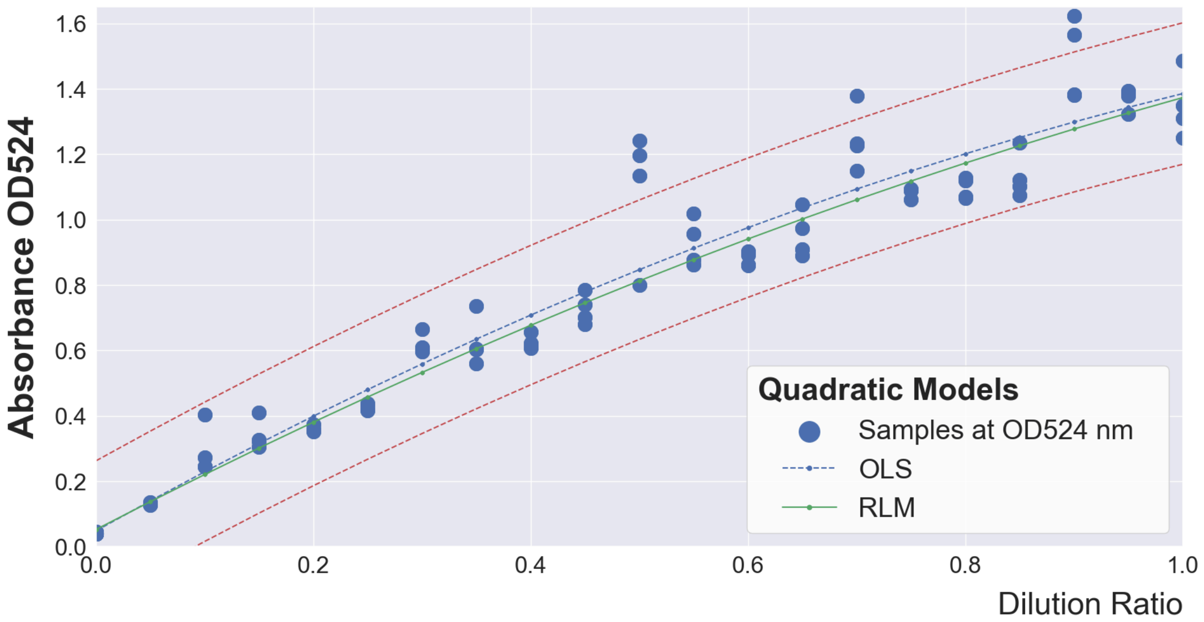

Figure S49 - Run 79 – Absorbance Spectrum and Regression at 524 nm

6.2 – Degradation Study

Mix Types: the degradation study was conducted on several plates with both low concentration mixes and high concentration mixes
Mix Preparation: all assays used fresh preparations - less than an hour old to remove all prior aging effects
Data Acquisition: the study consisted of monitoring a plate over 12 hours and measuring every hour the entire absorbance spectrum (350 n m to 650 nm).
Environmental Conditions: all assays were run at room temperature.

Figure S53 shows the comparative results for Run 84 - Plate 02 in Clariostar B (Low concentration mix Figure S50-A) and Run 79 - Plate 02 (also in Clariostar B - Figure S50-B). In all cases only the samples with the highest dilution ratios (80% and above) are displayed for legibility purposes, as they had the highest intensities and best showed the variations.
The low concentration mix (S50-A) exhibited at the start the distinctive three-peak shape of carotenoids, and showed little variation (a few percent at most) over the first two hours. The high concentration mix (S50-B) exhibited, from the start, a much flatter profile and degraded comparatively faster than the low concentration mix - retaining its appearance for less than an hour and its peaks all but disappearing after 4 hours.

We postulate that the main driver for the transformation is an aggregation process where molecules of lycopene bind to other molecules of lycopene. Such a process would be concentration-dependent (the more concentrated, the more likely molecules of lycopene are to find other molecules to bind to) and would be compatible with the mentions of micro-crystals found in our literature review. Furthermore, the older the mix, the bigger the clumps of lycopene are - and the more denatured the spectrum. Some degradation by oxidation - mentioned in several publications - could not be ruled out as a secondary driver, but failed to explain the concentration-dependence.

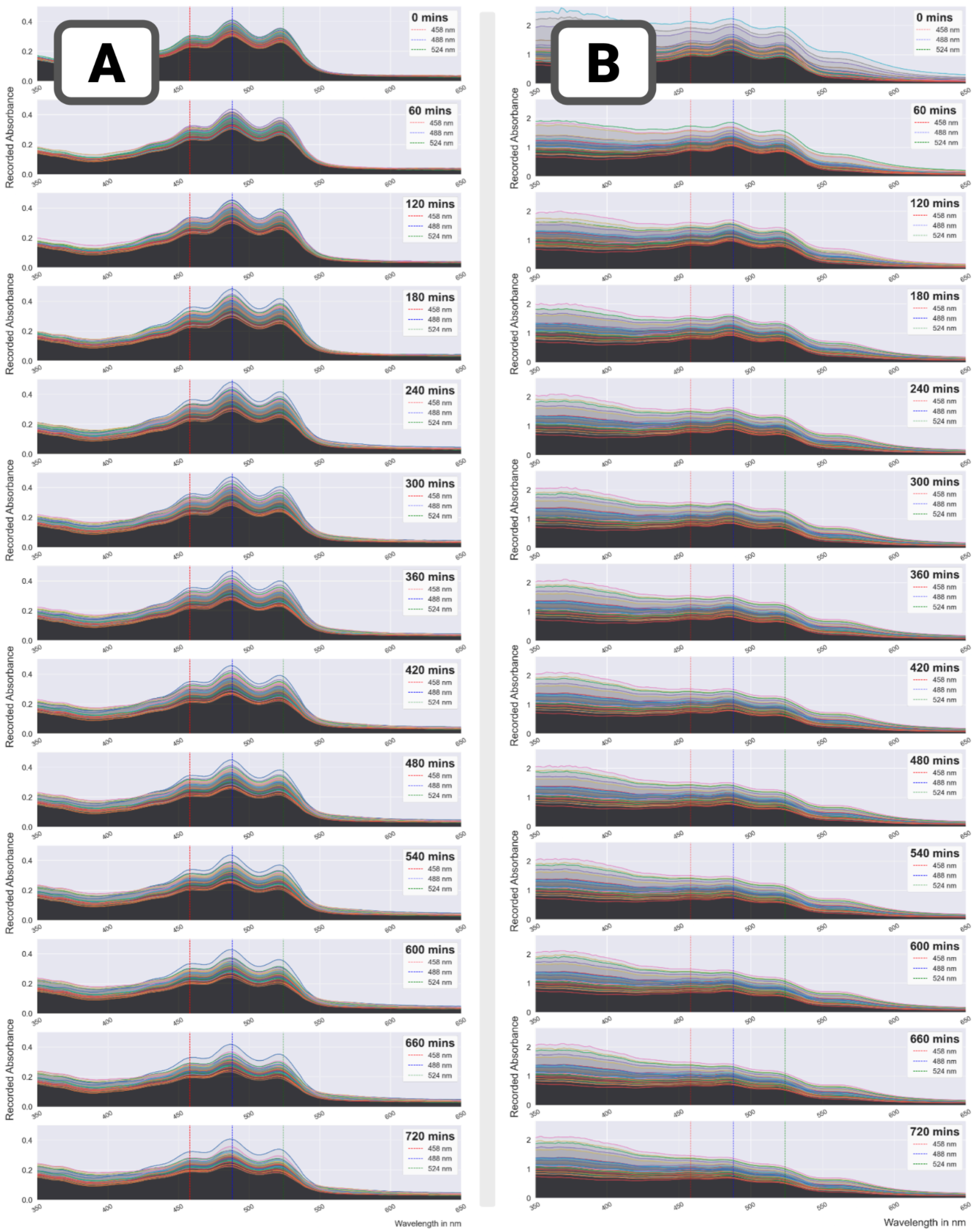

Figure S50 - Degradation Study of Lycopene in DMSO - Low Concentration Mix and High Concentration Mix. (A): Low concentration Mix - Run 84 - Plate 02 in Clariostar B. (B): High concentration Mix Run 79 - Plate 02 also in Clariostar B

6.3 – Measurement Study

Findings from all the previous phases of the study were incorporated into the following measurement protocol. The protocol comprises three steps - each designed to account for an identified source of variability in the measurements. When collected with this protocol, all measurements can then be considered equivalent (either technical repeats or carried out on similar objects), and processed accordingly.

<table>
<tr><th colspan="2">Final Measurement Protocol</th></tr>
<tr><td>Step 1</td><td>Minimizing the variation between batches</td></tr>
<tr><td colspan="2">Only use lycopene mixes (high and low concentration) that were prepared according to the protocol specified in the Methods section.
Only use fresh mixes (assumed free of all aging effects).
Check absorbance spectra a posteriori for sameness.</td></tr>
<tr><td>Step 2</td><td>Applying identical treatment to all plates from a batch</td></tr>
<tr><td colspan="2">Process plates in parallel (same platform, at the same time, and using the same version of the protocol)
Ensure processing is conducted in a time short enough for all degradation effects to be negligible</td></tr>
<tr><td>Step 3</td><td>Minimizing the variation between measurements</td></tr>
<tr><td colspan="2">Since degradation was proven to be minimal over a 30-40 minute time scale:
Repeat measurements every 2 minutes 6 or 10 times in a row
Use two separate Clariostar platforms (Clariostar A and B) if possible, in order to account for the choice of instrument</td></tr>
</table>

Table S7 - The Final Study Protocol.

Results below correspond to Run 085 – a high concentration lycopene mix. All technical repeats (measurements 1,3,5,7 and 9) were taken in a time scale of less than 30 minutes. Some samples were going on a “journey” - many regressing towards the trend of the dataset from the outset, some deviating (over a few measurements before returning to the trend.

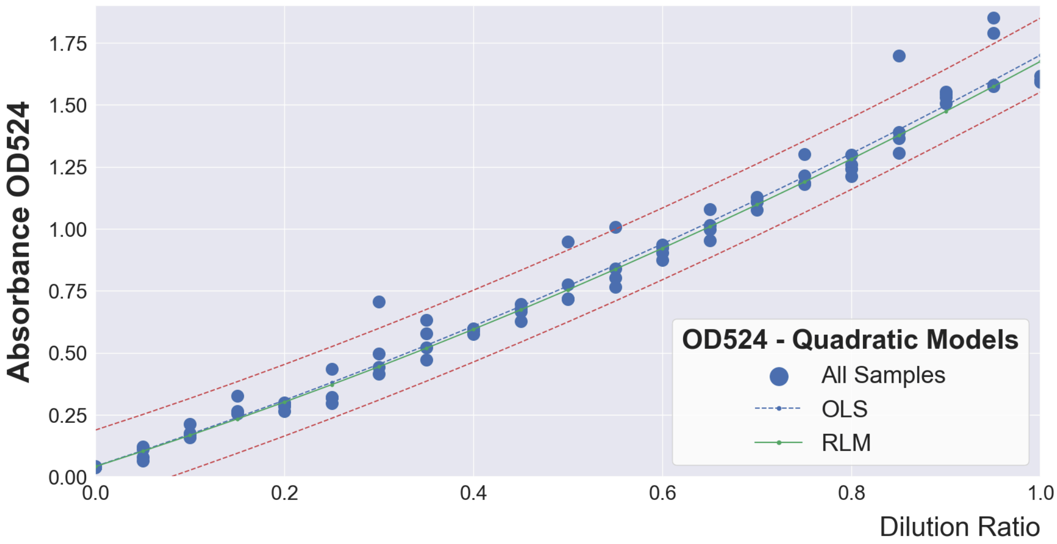

Absorbance OD524
Dilution Ratio
OD524 - Quadratic Models
All Samples
OLS
RLM

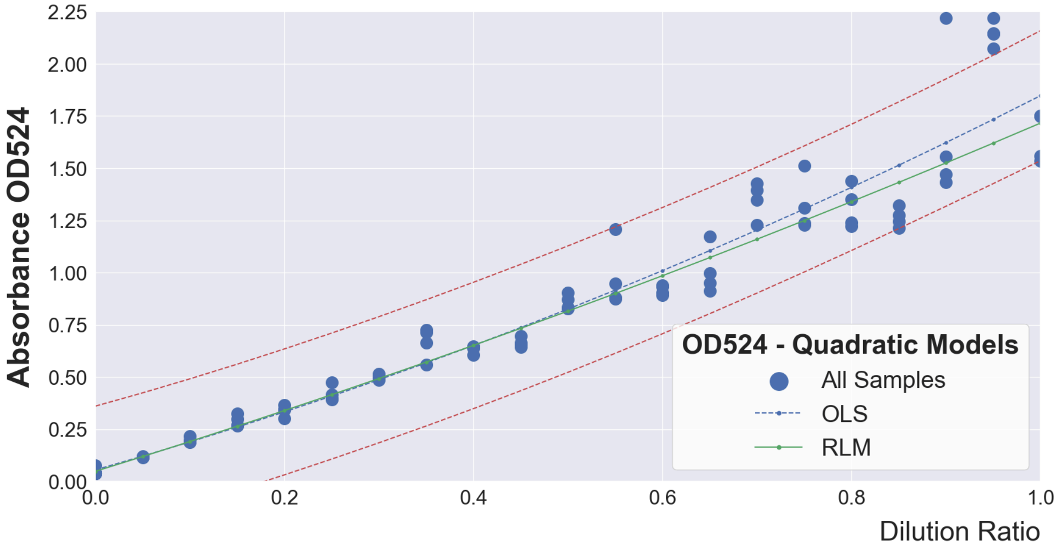

Absorbance OD524
Dilution Ratio
OD524 - Quadratic Models
All Samples
OLS
RLM

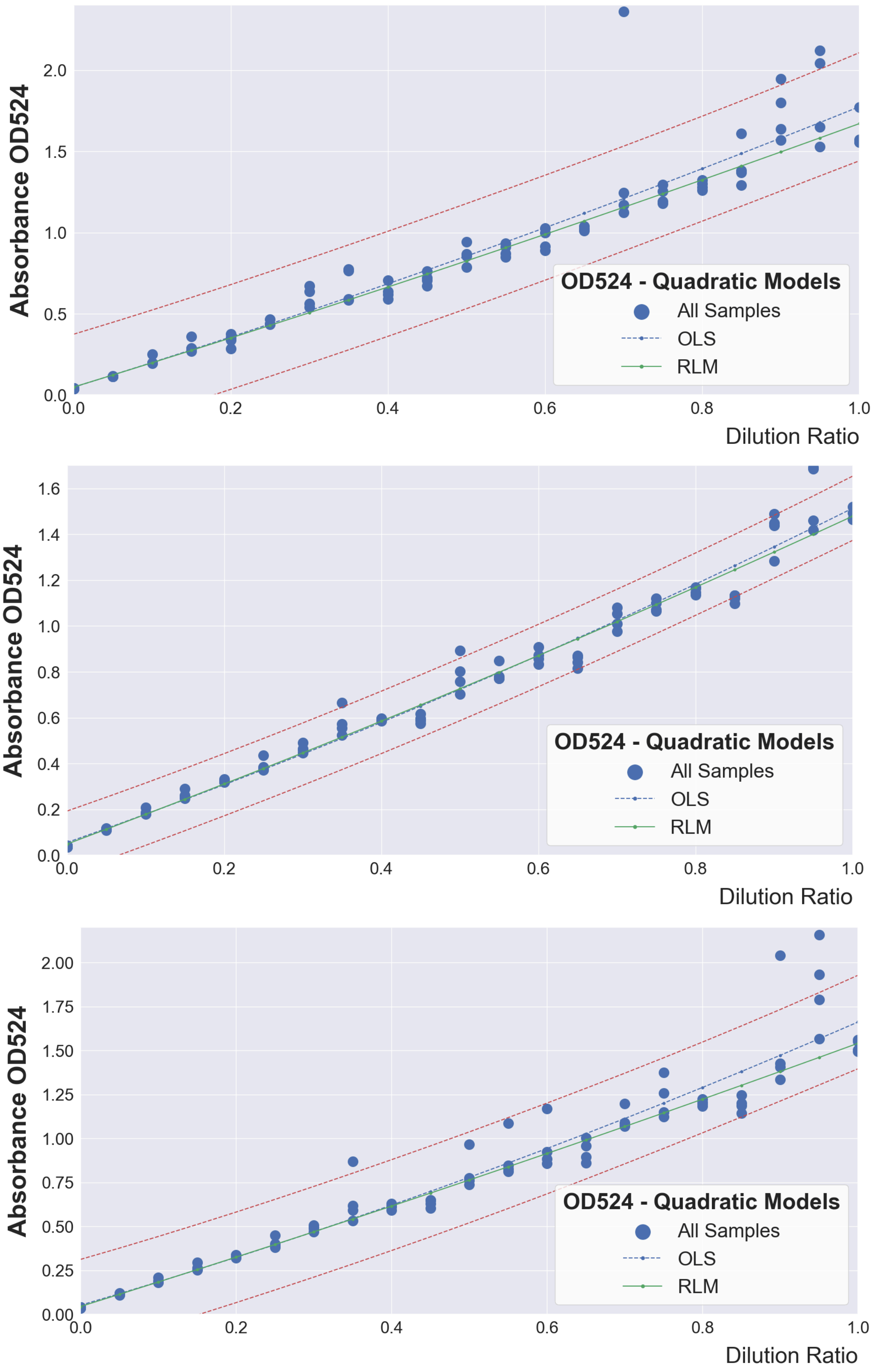

Figure S51 - Evolution of the Repeats of Run 085 - Repeats 1,3,5,7 and 9 at 524 nm

7 - Automated Protocols

Section 7 describes the automated protocols and their implementations. All protocols were run on a Cybio-Felix platform (https://shorturl.at/2RLaC).
Section 7.1 explains the nomenclature (based on semantic versioning) used throughout the study, lists the changes from one version to the next, and places the development history in context. Section 7.2 lists the values for the most important parameters for all the protocol versions. Finally, section 7.3 shows the CyBio-FeliX deck layout for both geometric and linear protocols, as well as a step-by-step visual representation of the protocols.

As a complement, a zip archive (LycopeneCalibration_Protocols.zip) containing the execution logs for the various protocol versions has also been shared alongside the manuscript and the present document.

7.1 - Protocol Nomenclature and History

All versions of the automated protocols were named according to a nomenclature in common use in software-engineering (semantic versioning) : V (i.e version) X.Y.Z, where:

- The first digit (X) corresponds to the general approach to dilution: 1 was used for the sequential dilution approach (column with column), whereas 2 was used for the linear dilution approach.
- The second digit (Y) was incremented when a major revision was introduced, while the final digit (Z) was incremented for every minor revision.

It is worth stressing that the development of the protocols V 1.X.Y (geometric scheme) and V 2.X.Y (linear scheme) did not proceed independently, but incrementally. The input of the Analytik-Jena engineer was key.

- **Protocols V 1.X.Y (geometric scheme).**
    - The protocols for the geometric scheme were developed first (they were simpler to implement due to the repeated column-to-column transfers).
    - The aspiration and dispensation speeds parameters were adjusted for both reservoirs; a blowout was introduced as well on the advice of our support engineer (these are common steps to deal with complex liquids).
    - The adjustments were not enough - so the development of the protocol was aborted
- **Protocols V 2.X.Y (linear scheme).**
    - When switching to the linear scheme, the parameters assigned to the last protocols for the geometric scheme (V 1.3.0 and V 1.3.1) were carried over to the development of the protocols V 2.X.Y. This explains why they are identical to the parameters for the initial protocol V 2.0.0.
    - On the advice of the support engineer, the drive speeds were reduced to a minimum. The dispense speed of the mix was also increased to prevent clogging.
    - Further minor modifications were attempted, with no visible benefits.

Geometric (Sequential) Dilution Scheme V 1.Y.Z		
**Version**	**Platform**	**Notes**
**V 1.0.0**	Felix	**Development Runs**<br>Original Version
**V 1.1.0**	Felix	N/A
**V 1.2.0**	Felix	**Acquisition Runs**<br>Pipetting column 1 in flatbottom at the end with col (2-10)<br>Pause after aspirate and dispense<br>Mixing dab-off and blow-out at higher positions above liquid in the well
**V 1.3.0**	Felix	**Acquisition Runs - Modification**<br>Dispense and aspirate speeds adjusted to 10 and 50 for solvent and mix respectively<br>No improvement (Data not shown)
**V 1.3.1**	Felix	**Acquisition Runs - Modification**<br>Using new tips for serial dilution<br>No improvement (Data not shown)

Table S8 - The Automated Protocol V 1.Y.Z and its Revisions

Linear Dilution Scheme V 2.Y.Z		
**Version**	**Platform**	**Notes**
**V 2.0.0**	Felix	**Development Runs**<br>All parameters set to match the values in 1.3.X
**V 2.0.3**	Felix	**Development Runs**<br>Changed Z drive speed to 10m/s before aspiration in DMSO reservoir<br><br>Mix dispense speed increased to prevent clogging
**V 2.1.0**	Felix	**Acquisition Runs**<br>Blowout adjusted down from 5 to 3ul
**V 2.1.1**	Felix	**Acquisition Runs - Settled Version**<br>Changed Z drive speed to 10m/s everywhere after dispensing in DMSO reservoir
**V 2.1.2**	Felix	**Acquisition Runs - Modification 1**<br>5x mix up/down for mixing in flatbottom plate, instead of 3x<br><br>No improvement (Data not shown)
**V 2.2.0**	Felix	**Acquisition Runs - Modification 2**<br>Added pre-wet and 20ul-asp/15uL-dispense before aspirations (as per Analytik-Jena engineer Chris Hirst's suggestion)<br><br>No improvement (Data not shown)

Table S9 - The Automated Protocol V 2.Y.Z and its Revisions

7.2 - Corresponding Parameters

Geometric (Sequential) Dilution Scheme V 1.Y.Z					
**Parameter**	**V 1.0.0**	**V 1.1.0**	**V 1.2.0**	**V 1.3.0**	**V 1.3.1**
**Head Used**	SELECT - 8 Channel (250 $\mu\ell$)				
**Tips Used**	Non Filter Tips (250 $\mu\ell$)				
**Air Gap**	Not Needed on Felix Platforms				
**Speed Aspirate ($\mu\ell$/s)**	105 (Default)	105 (Default)	105 (Default)	10	10
**Speed Dispense ($\mu\ell$/s)**	105 (Default)	105 (Default)	105 (Default)	10	10
**Speed Aspirate Mix ($\mu\ell$/s)**	105 (Default)	105 (Default)	105 (Default)	50	50
**Speed Dispense Mix ($\mu\ell$/s)**	105 (Default)	105 (Default)	105 (Default)	50	50
**Drive Speed Z Up (mm/s)**	130 (Default)	130 (Default)	130 (Default)	130 (Default)	130 (Default)
**Drive Speed Z Down (mm/s)**	130 (Default)	130 (Default)	130 (Default)	130 (Default)	130 (Default)
**Rinse/ Mix**	x3	x3	x3	x3	x3
**Wait after Aspirate / Dispense (s)**	N/A	N/A	1	N/A	N/A
**Blowout ($\mu\ell$)**	N/A	N/A	N/A	5	5
**Pre-Wet**	False	False	False	False	False

Table S10 - The Automated Protocol V 1.Y.Z parameters

**Linear Dilution Scheme V 2.Y.Z**						
**Parameter**	**V 2.0.0**	**V 2.0.3**	**V 2.1.0**	**V 2.1.1**	**V 2.1.2**	**V 2.2.0**
**Head Used**	SELECT - 8 Channel (250 $\mu\ell$)					
**Tips Used**	Non Filter Tips (250 $\mu\ell$)					
**Air Gap**	Not Needed on Felix Platforms					
**Speed Aspirate ($\mu\ell$/s)**	10	10	50	50	50	50
**Speed Dispense ($\mu\ell$/s)**	10	10	50	50	50	50
**Speed Aspirate Mix ($\mu\ell$/s)**	50	50	50	50	50	50
**Speed Dispense Mix ($\mu\ell$/s)**	50	80	80	80	80	80
**Drive Speed Z Up (mm/s)**	130 (Default)	10	10	10	10	10
**Drive Speed Z Down (mm/s)**	130 (Default)	130 (Default)	130 (Default)	130 (Default)	130 (Default)	130 (Default)
**Rinse/ Mix**	x3	x3	x3	x3	x3	x3
**Wait after Aspirate / Dispense (s)**	N/A	N/A	N/A	N/A	N/A	0.2
**Blowout ($\mu\ell$)**	5	5	3	3	3	3
**Pre-Wet**	False	False	False	False	False	True

Table S11 - The Automated Protocol V 2.Y.Z parameters

7.3 - Automated Serial Dilution Protocol

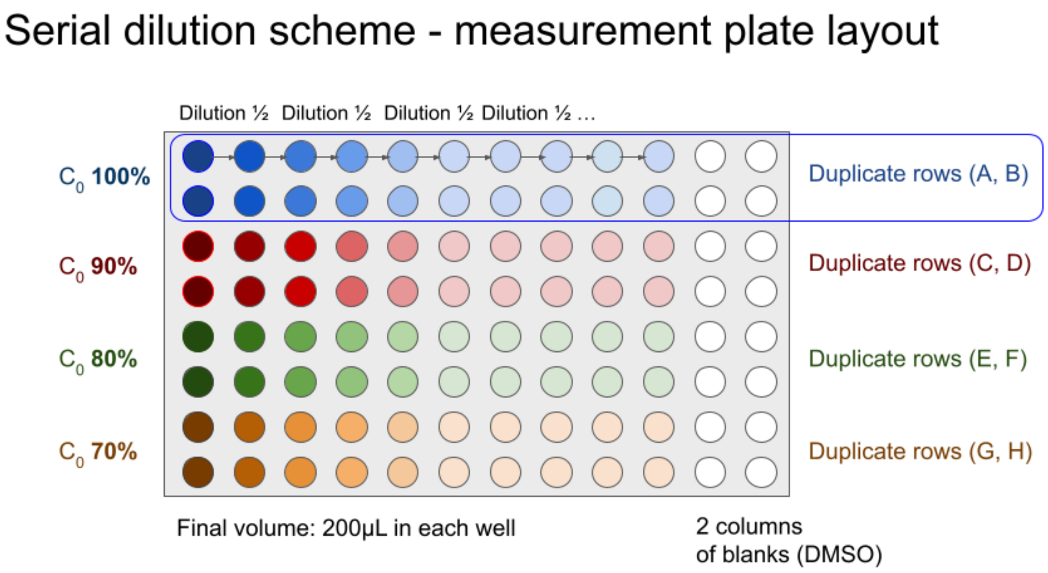

Figure S52 - Automated Serial Dilution Protocol - Plate Map

CyBio FeliX deck layout

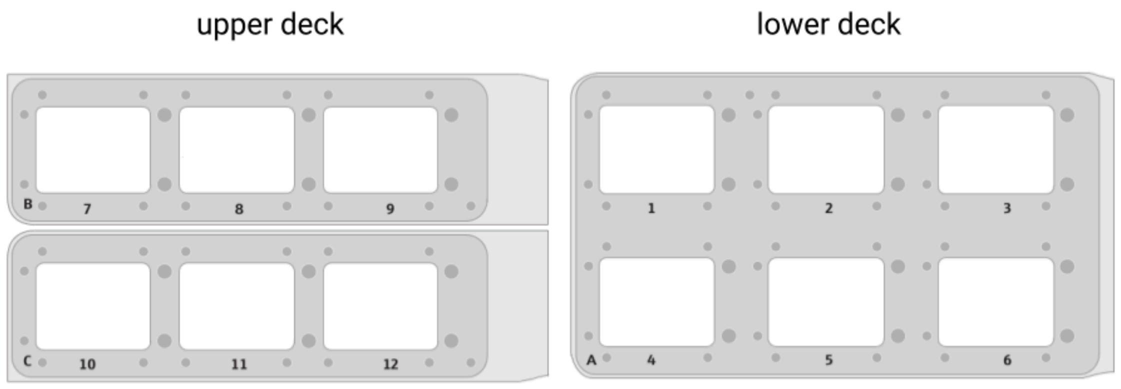

Serial dilution

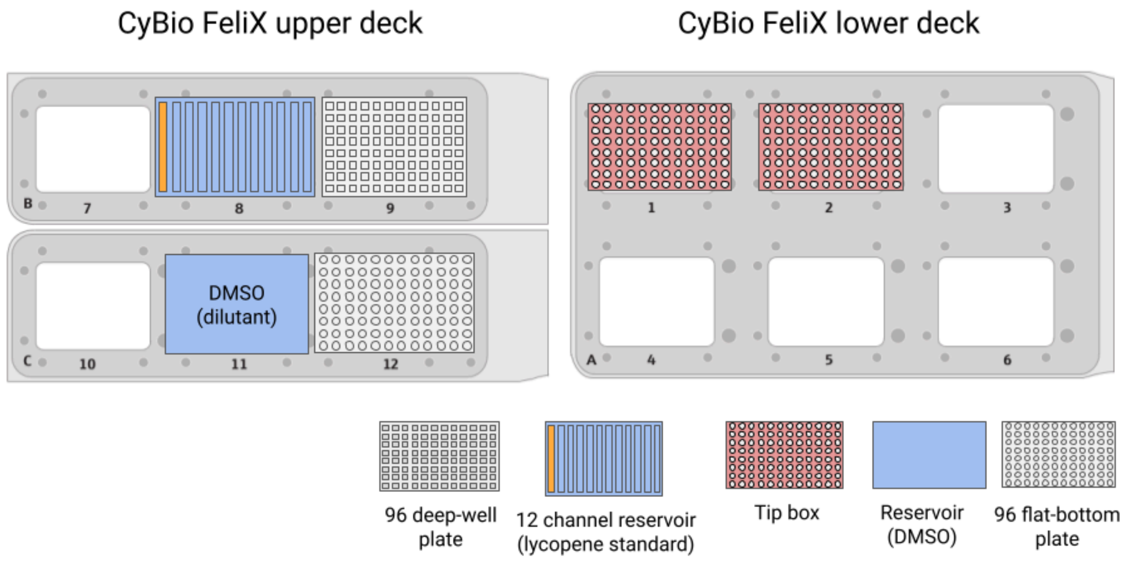

Figure S53 - Automated Serial Dilution Protocol - Robotic Liquid Handler (Felix) Deck Setup

Step 1

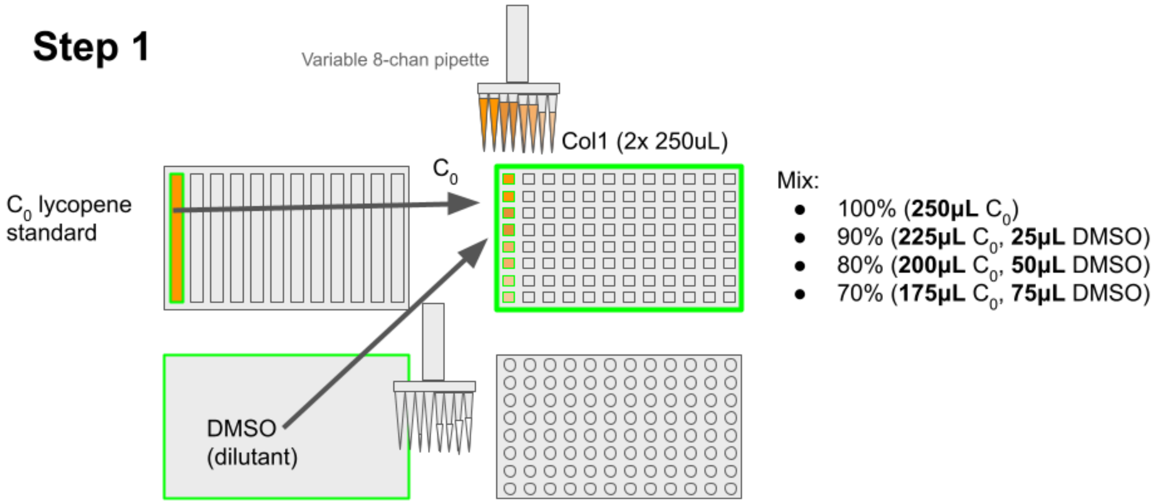

Variable 8-chan pipette
Col1 (2x 250uL)
C0
C0 lycopene standard
DMSO (dilutant)
Mix:
100% (250µL C0)
90% (225µL C0, 25µL DMSO)
80% (200µL C0, 50µL DMSO)
70% (175µL C0, 75µL DMSO)

Step 2

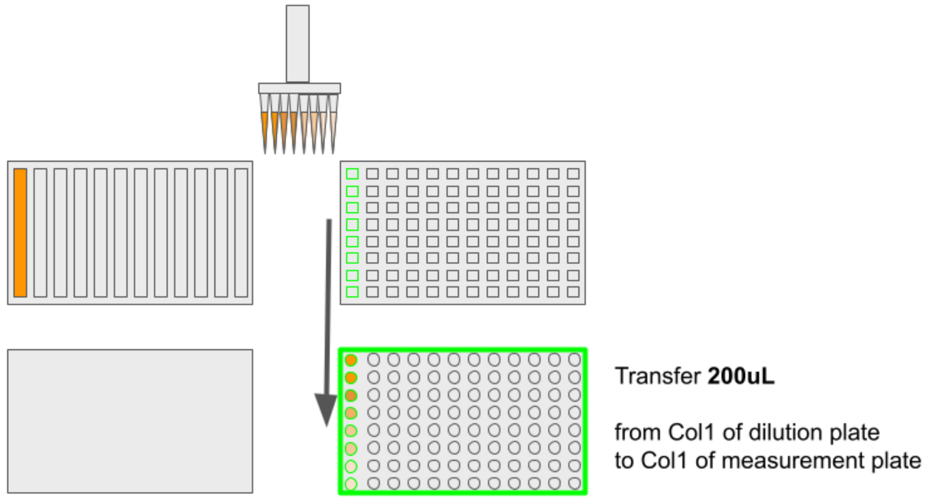

Transfer 200uL
from Col1 of dilution plate
to Col1 of measurement plate

Step 3

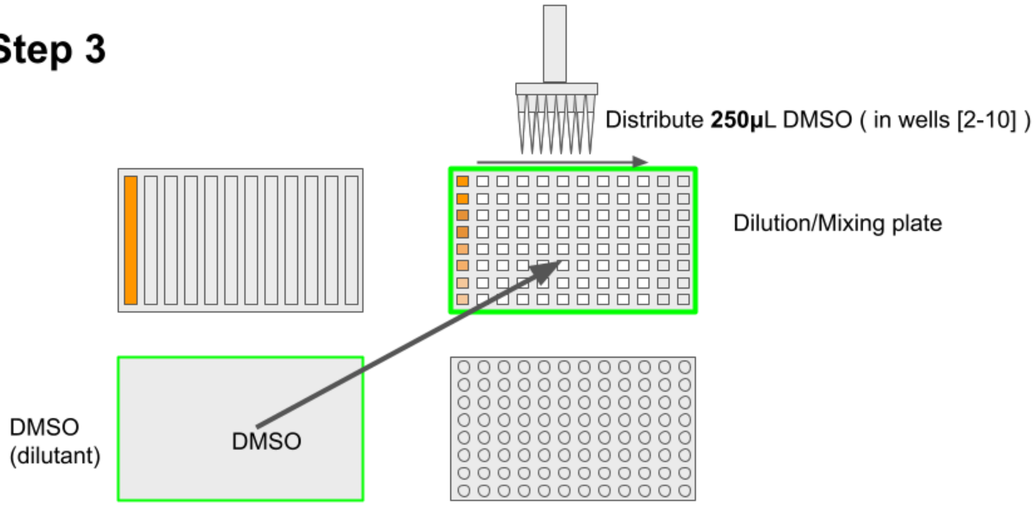

Distribute **250µL** DMSO (in wells [2-10])
Dilution/Mixing plate
DMSO
(dilutant)
DMSO

Step 4

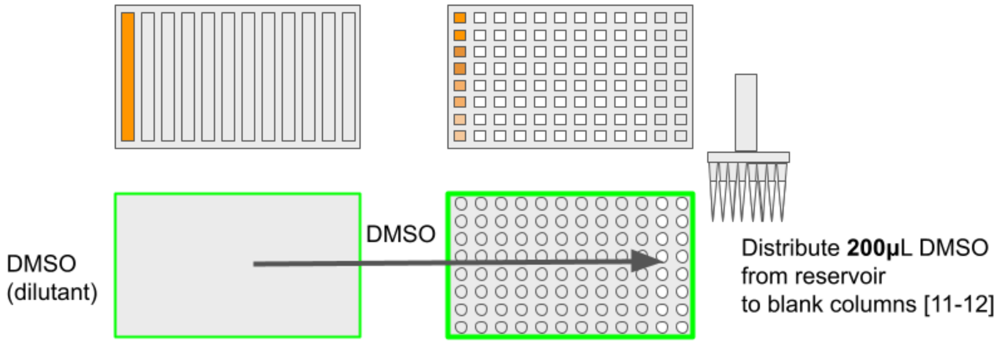

DMSO
(dilutant)
DMSO
Distribute **200µL** DMSO
from reservoir
to blank columns [11-12]

Step 5

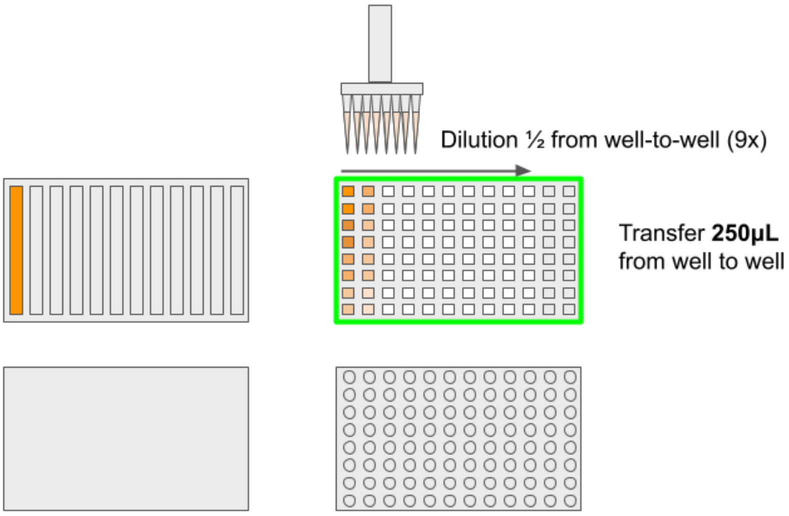

i

Step 6

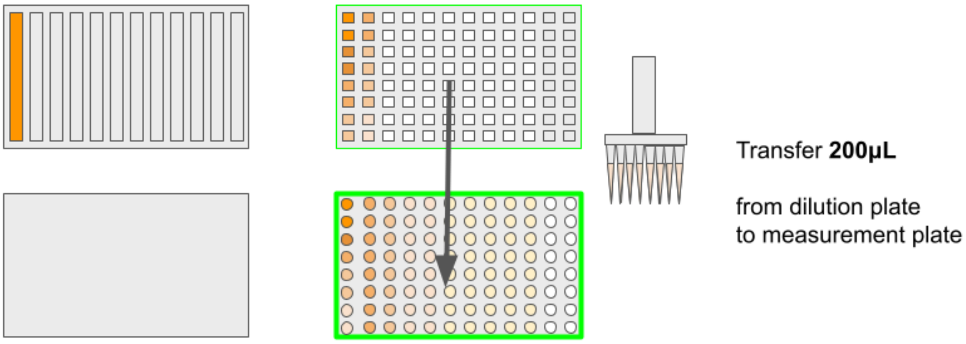

Figure S54 - Automated Serial Dilution Protocol Steps

7.4 - Automated Linear Dilution Protocol

Linear dilution scheme - measurement plate layout

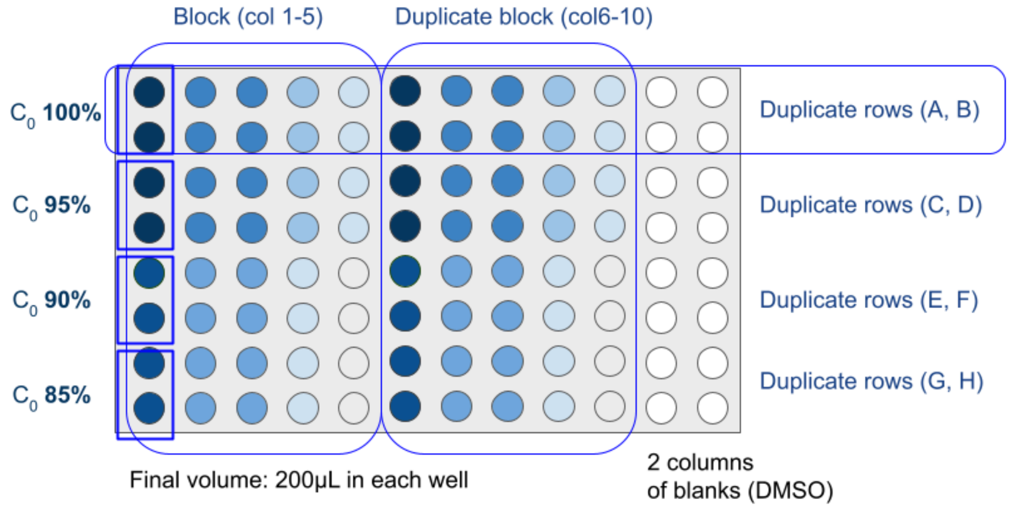

Dilution from 100% to 5%	Dilution (repeat)	Blanks

A1

100 %	80 %	60 %	40 %	20 %	100%	80 %	60 %	40 %	20 %	B	B
100 %	80 %	60 %	40 %	20 %	100%	80 %	60 %	40 %	20 %	B	B
95 %	75 %	55 %	35 %	15 %	95 %	75 %	55 %	35 %	15 %	B	B
95 %	75 %	55 %	35 %	15 %	95 %	75 %	55 %	35 %	15 %	B	B
90 %	70 %	50 %	30 %	10 %	90 %	70 %	50 %	30 %	10 %	B	B
90 %	70 %	50 %	30 %	10 %	90 %	70 %	50 %	30 %	10 %	B	B
85 %	65 %	45 %	25 %	5 %	85 %	65 %	45 %	25 %	5 %	B	B
85 %	65 %	45 %	25 %	5 %	85 %	65 %	45 %	25 %	5 %	B	B

Figure S55 - Automated LinearDilution Protocol - Plate Map

CyBio FeliX deck layout

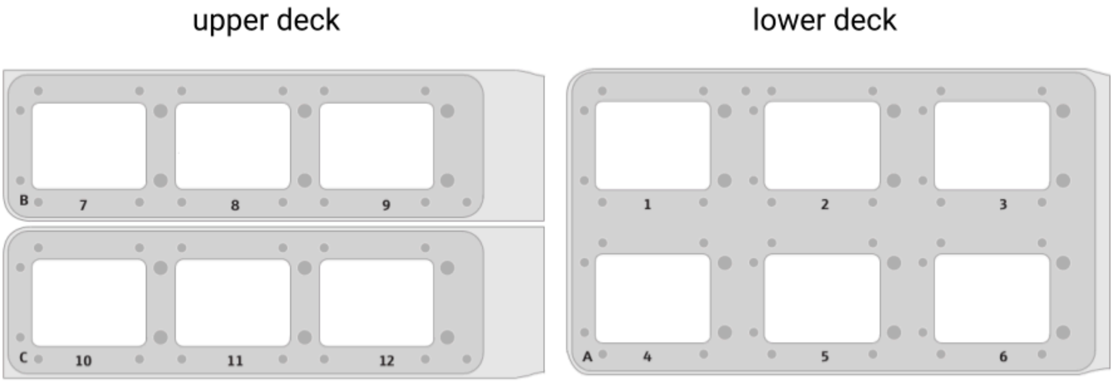

Linear dilution

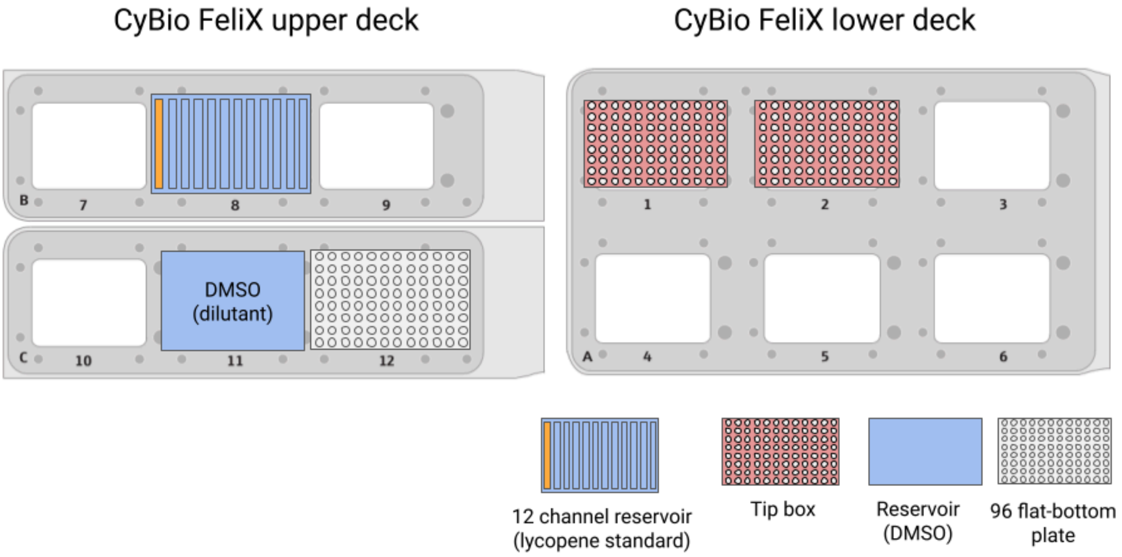

Figure S56 - Automated Linear Dilution Protocol - Robotic Liquid Handler (Felix) Deck Setup

Step 1

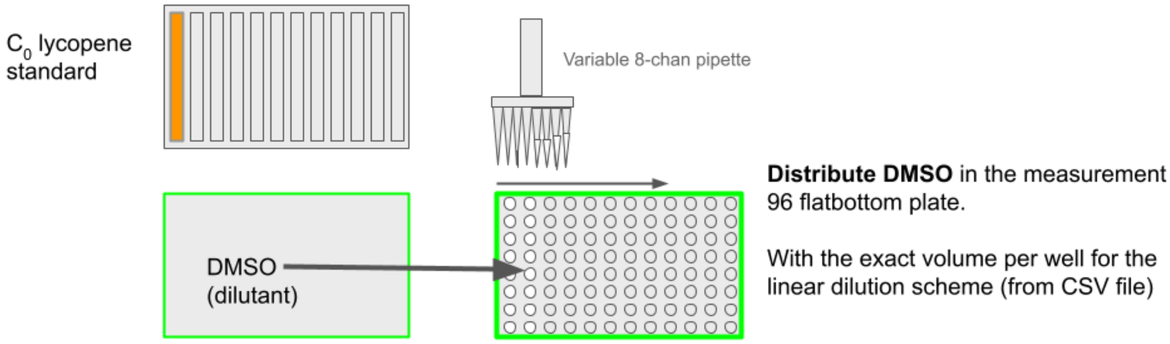

Step 2

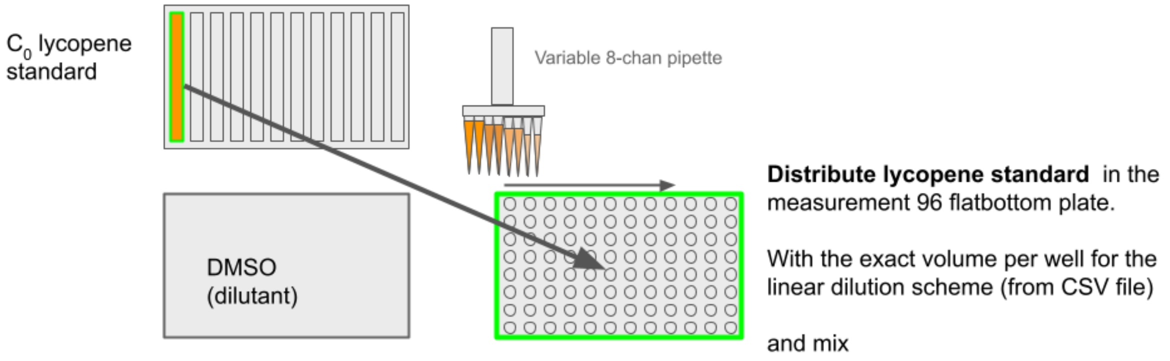

Figure S57 - Automated Serial Dilution Protocol Steps
